# Spatial Dynamics of Human-Origin H1 Influenza A Virus in North American Swine

**DOI:** 10.1371/journal.ppat.1002077

**Published:** 2011-06-09

**Authors:** Martha I. Nelson, Philippe Lemey, Yi Tan, Amy Vincent, Tommy Tsan-Yuk Lam, Susan Detmer, Cécile Viboud, Marc A. Suchard, Andrew Rambaut, Edward C. Holmes, Marie Gramer

**Affiliations:** 1 Division of International Epidemiology and Population Studies, Fogarty International Center, National Institutes of Health, Bethesda, Maryland, United States of America; 2 Department of Microbiology and Immunology, Katholieke Universiteit Leuven, Leuven, Belgium; 3 Virus and Prion Diseases of Livestock Research Unit, National Animal Disease Center, USDA-ARS, Ames, Iowa, United States of America; 4 Department of Biology, The Pennsylvania State University, University Park, Pennsylvania, United States of America; 5 The University of Minnesota Veterinary Diagnostic Laboratory, St. Paul, Minnesota, United States of America; 6 Departments of Biomathematics and Human Genetics, David Geffen School of Medicine at UCLA, and Department of Biostatistics, UCLA School of Public Health, Los Angeles, California, United States of America; 7 Institute of Evolutionary Biology, University of Edinburgh, Ashworth Laboratories, Edinburgh, United Kingdom; University of Texas at Austin, United States of America

## Abstract

The emergence and rapid global spread of the swine-origin H1N1/09 pandemic influenza A virus in humans underscores the importance of swine populations as reservoirs for genetically diverse influenza viruses with the potential to infect humans. However, despite their significance for animal and human health, relatively little is known about the phylogeography of swine influenza viruses in the United States. This study utilizes an expansive data set of hemagglutinin (HA1) sequences (n = 1516) from swine influenza viruses collected in North America during the period 2003–2010. With these data we investigate the spatial dissemination of a novel influenza virus of the H1 subtype that was introduced into the North American swine population via two separate human-to-swine transmission events around 2003. Bayesian phylogeographic analysis reveals that the spatial dissemination of this influenza virus in the US swine population follows long-distance swine movements from the Southern US to the Midwest, a corn-rich commercial center that imports millions of swine annually. Hence, multiple genetically diverse influenza viruses are introduced and co-circulate in the Midwest, providing the opportunity for genomic reassortment. Overall, the Midwest serves primarily as an ecological sink for swine influenza in the US, with sources of virus genetic diversity instead located in the Southeast (mainly North Carolina) and South-central (mainly Oklahoma) regions. Understanding the importance of long-distance pig transportation in the evolution and spatial dissemination of the influenza virus in swine may inform future strategies for the surveillance and control of influenza, and perhaps other swine pathogens.

## Author Summary

Since 1998, genetically and antigenically diverse influenza A viruses have circulated in North American swine due to continuous cross-species transmission and reassortment with avian and human influenza viruses, presenting a pandemic threat to humans. Millions of swine are transported year-round from the southern United States into the corn-rich Midwest, but the importance of these movements in the spatial dissemination and evolution of the influenza virus in swine is unknown. Using a large data set of influenza virus sequences collected in North American swine during 2003–2010, we investigated the spatial dynamics of two influenza viruses of the H1 subtype that were introduced into swine from humans around 2003. Employing recently developed Bayesian phylogeography methods, we find that the spread of this influenza virus follows the large-scale transport of swine from the South to the Midwest. Based on this pattern of viral migration, we suggest that the genetic diversity of swine influenza viruses in the Midwest is continually augmented by the importation of viruses from source populations located in the South. Understanding the importance of long-distance pig movements in the evolution and spatial dissemination of influenza virus in swine may inform future strategies for the surveillance and control of influenza, and perhaps other swine pathogens.

## Introduction

Swine influenza A viruses cause severe respiratory disease in pigs, similar to that which presents in humans, and constitute an important economic concern for the US swine industry and threat to public health. Influenza was first clinically recognized in pigs in the Midwestern US in conjunction with the severe 1918 ‘Spanish flu’ H1N1 pandemic in humans [1], although whether the pandemic originated in humans or pigs remains unresolved [2]. Periodic transmission of influenza viruses between humans and swine occurs in both directions, including such notable cases as the 1976 outbreak of swine A/H1N1 influenza virus in humans in Fort Dix, New Jersey [3] and the 2009 swine-origin A/H1N1 pandemic virus in humans [4], [5]. The 1918-origin ‘classical’ H1N1 swine influenza virus circulated in US swine for 80 years with relatively few antigenic changes [6], but in the last decade the antigenic diversity of swine influenza viruses in the US has multiplied, stimulating research, development, and uptake of influenza vaccines in the US swine industry.

**Figure 1:**
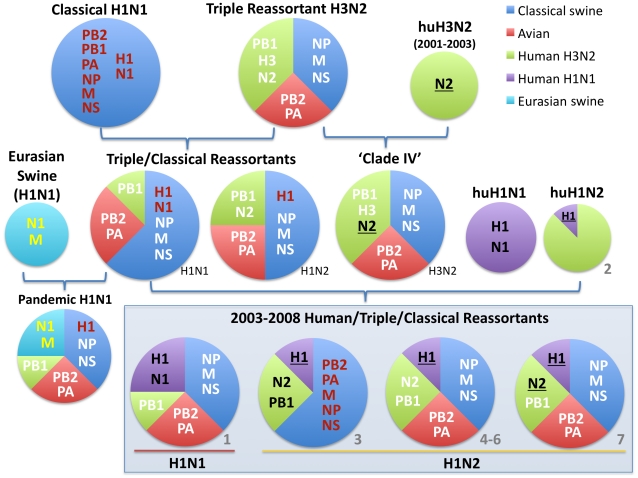
Evolutionary origins of H1 swine influenza viruses in North America. Circles are color-coded according to the evolutionary origins of the 8 segments that comprise the viral genome (PB2, PB1, PA, HA, NP, NA, M, and NS): blue  =  classical swine influenza virus, red  =  avian influenza virus, green  =  human H3N2 influenza virus, purple  =  human (seasonal) H1N1 influenza virus, and light blue  =  Eurasian avian-origin H1N1 swine influenza virus. The shading and underlining of letters provides additional indication of whether a particular segment is descended from the 1998 triple reassortant swine viruses (white), classical swine influenza viruses (red), or Eurasian swine influenza viruses (yellow). The ‘2003–2008 Human/Triple/Classical Reassortant’ viruses represent those characterized in this study (Table 1, Figs. 3 and 4). The numbers in gray (1–7) and all color codes correspond to those used in Table 1 and Figs. 3 and 4, with additional bold black shading and underlining of H1 and N2 segments included to show evolutionary linkages in cases where evolutionary origins may otherwise be ambiguous. Asterisks represent novel reassortment events identified in this study.

Currently, influenza A viruses of the H1N1, H1N2, and H3N2 subtypes all co-circulate in US swine. In 1998–1999, a triple reassortant H3N2 influenza virus emerged in US swine that possessed HA (H3), NA (N2), and PB1 segments of human H3N2 virus origin, PB2 and PA segments of avian virus origin, and NP, M1/2, and NS1/2 segments of classical swine virus origin [7] (Fig. 1). Over the next decade these H3N2 triple reassortant swine viruses further reassorted with human H3N2 viruses [8], [9], as well as with the co-circulating H1N1 classical swine viruses [10], [11]. Mainly these reassortment events involved the HA and NA segments, preserving what has been termed the ‘triple reassortant internal genes’ (TRIG) constellation (avian-origin PB2 and PA, human H3N2-origin PB1, and classical swine-origin NP, M1/2, and NS1/2).

In 2003 influenza A virus of entirely human H1N2 origin was identified in Canadian swine [12], and in 2005 H1N1 viruses with human-origin H1 and N1 segments were identified in the United States, representing two separate introductions of human H1 virus into swine that were referred to as ‘δ-1’ (H1N2) and ‘δ -2’ (H1N1) lineages based on the order of identification [13]. These human-H1 origin swine viruses also acquired novel genome segments via reassortment with other swine and human influenza viruses [12], [13].

Globally, the swine influenza virus population is spatially separated into the North American and Eurasian lineages, although both lineages co-circulate in Asia, which imports swine from North America and Europe. In the US the traditional center of swine production is located in the ‘Corn Belt’ of the Midwest, including Iowa, Illinois, Indiana, and Minnesota [14]. Beginning in the 1970's, swine production expanded into large new facilities located in the Southeastern US, mainly North Carolina, and more recently into Oklahoma in the South-central US [15]. Due to the lower cost of transporting swine versus the required amount of feed, the majority of swine born in the South-central and Southeastern regions are transported by road to the Midwestern Corn Belt to be fattened and slaughtered, resulting in continuous large-scale movements of swine (‘swine-flows’) into the Midwest [14]. However, the role of local, regional, and global swine-flows in the ecology and evolution of swine influenza viruses remains unclear.

The aim of our study was to investigate the role of inter-regional swine-flows in the spatial dissemination of newly introduced swine viruses in the US, using the human-origin A/H1 influenza virus as a case study. We utilize HA1 sequence data from a large data set of swine influenza virus isolates (n = 1,516 sequences) collected from 23 US states during 2003–2010 and apply recently developed methods of Bayesian phylogeography. The strength of the Bayesian approach is that the diffusion process among discrete location states is integrated with time-scaled phylogenies that incorporate phylogenetic uncertainty. This approach provides a formal framework to test hypotheses about viral diffusion processes driven by known population distributions and movements.

## Results

### Phylogenetic analysis

**Figure 2:**
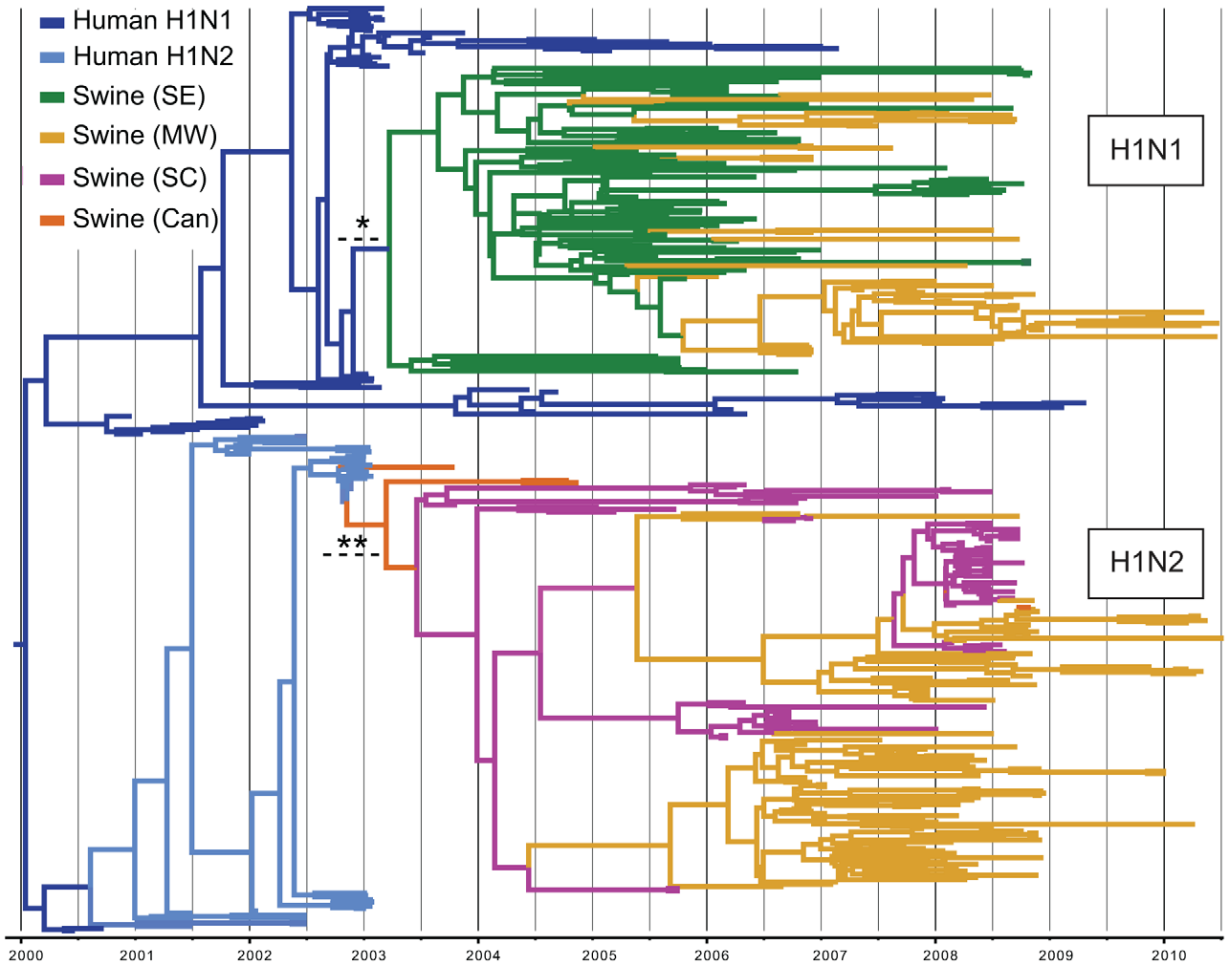
Phylogenetic relationships of 325 human H1-origin swine influenza viruses. Time-scaled Bayesian MCC tree of 325 HA1 (H1) sequences from human H1-origin influenza viruses collected from North American swine during 2003–2010, and 57 and 35 human H1N1 and H1N2 sequences, respectively, collected during 2000–2010 that are included as background. Tip labels have been removed (available in Fig. S10) and branches are colored by host species, subtype, and most probable location state: dark blue  =  human H1N1, light blue  =  human H1N2, green  =  swine influenza viruses collected in the Southeastern US (SE), yellow - swine influenza viruses collected in the Midwestern US (MW), magenta  =  swine influenza viruses collected in the South-central US (SC), and orange  =  swine influenza viruses collected in Canada (Can). Dotted lines with asterisks represent the estimated time period for human-to-swine transmission of H1N1 (*) and H1N2 (**) viruses, and posterior probabilities >0.90 for key nodes are included.

Of the 1,516 HA1 (H1) influenza virus sequences collected from swine in the United States and Canada from 2003–2010 that were included in this study, 41 were related to the human pandemic H1N1/09 virus, all of which were collected in 2009–2010 and appear to result from multiple human-to-swine transmission events. These pandemic viruses have been described previously and thus are not the focus of the present study [16]. Of the remaining 1,475 swine viruses, 327 were phylogenetically related to seasonal human H1 viruses (Fig. S1), which constitute two phylogenetically distinct clusters, representing two contemporaneous, but independent introductions of different human influenza viruses into swine (Fig. 2), consistent with previous findings [13]. Both of these clusters are phylogenetically most closely related to human H1 influenza viruses collected in early 2003. One cluster (n = 138 sequences) is related to widespread human seasonal A/H1N1 virus, while the other cluster (n = 187 sequences) is related to a less common human reassortant A/H1N2 virus that circulated globally in humans from 2001–2003. The A/H1N2 reassortant virus contains an HA derived from human seasonal H1N1 viruses and 7 segments of human H3N2 influenza virus origin [17].

We estimated the Time to the Most Recent Common Ancestor (TMRCA) for the nodes adjoining the branch that represents the human-to-swine transmission events of the H1N1 and H1N2 viruses. Accordingly, the cross-species transmission of H1N1 from humans into swine is estimated to have occurred during the period October 2002–March 2003, which coincides with the timing of the A/H1N1-dominant 2002–2003 winter influenza epidemic in humans in North America [18] (Fig. 2, Table S1). Similarly, the timeframe for the cross-species transmission of the H1N2 virus into swine is estimated to be August 2002–February 2003, which overlaps with the time period when A/H1N2 viruses circulated in humans in North America (Table S1).

**Figure 3:**
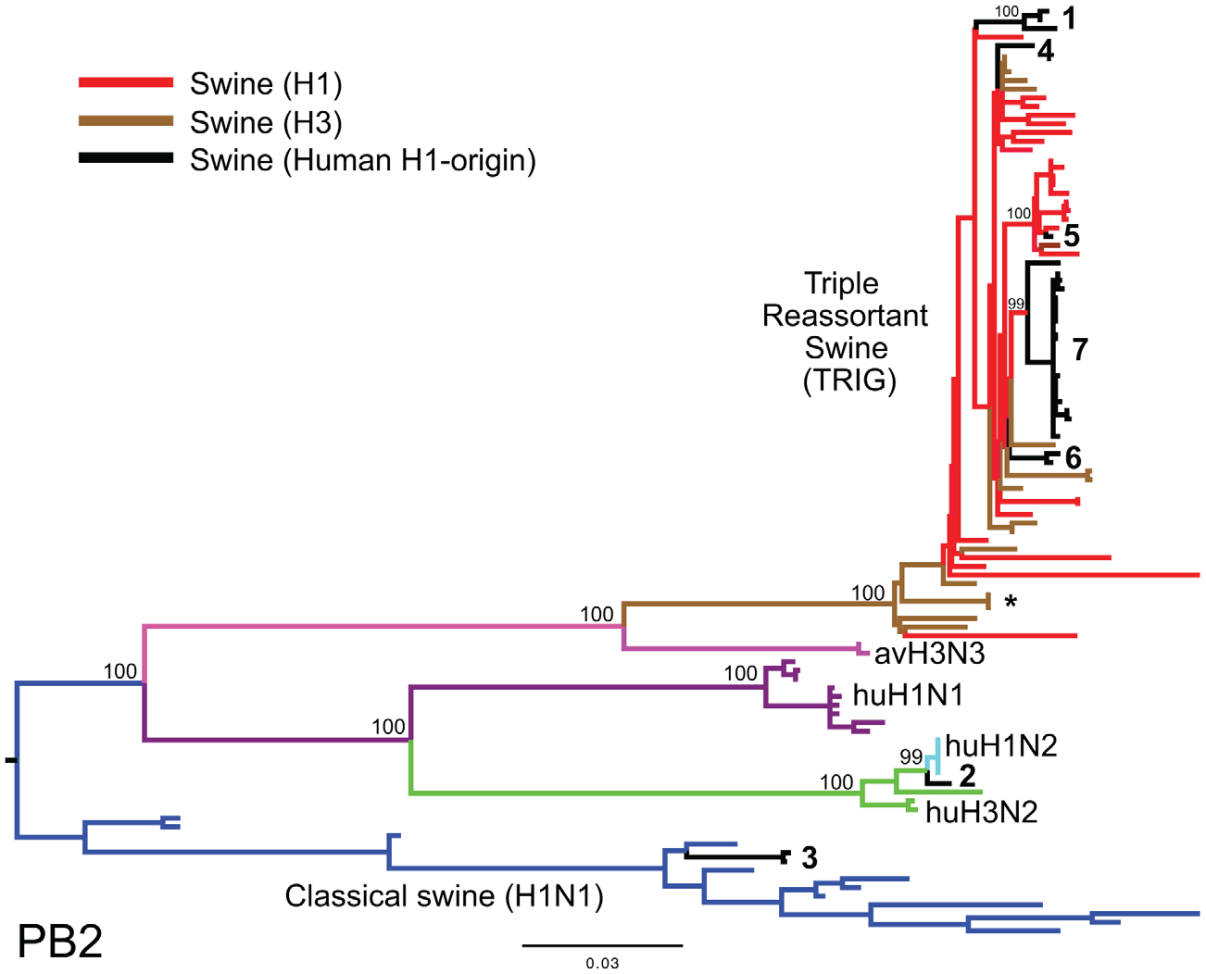
Phylogenetic relationships of the PB2 segment. Phylogenetic relationships of the PB2 segment (2,277 nt) of the 31 human H1-origin swine influenza viruses for which whole-genome sequences are available (2003–2008) and, as background, 12 classical swine isolates (1931–2004), triple reassortant swine viruses of H3 (n = 18) and H1 (n = 26) subtypes (1998–2009), 2 avian-H3N3 origin swine isolates, and 15 representative H3N2, H1N2, and H1N1 human influenza isolates. The maximum likelihood tree is mid-point rooted for clarity, and all branch lengths are drawn to scale. Bootstrap values >70% are included for key nodes. Shading of branches, according to evolutionary origins of tip isolate, is similar to those used in Table 1: black  =  human H1-origin swine influenza viruses, purple  =  human H1N1, green  =  human H3N2, light blue  =  human H1N2, blue  =  classical swine influenza viruses, pink  =  avian H3N3-origin swine isolates, red  =  triple reassortant swine influenza viruses (H1), and brown  =  triple reassortant swine influenza viruses (H3). Numbers associated with human-origin swine influenza viruses also correspond to those listed in Table 1: 1 =  A/sw/IL/00685/2005(H1N1), A/sw/NC/00573/2005(H1N1), and A/sw/Minnesota/07002083/2007(H1N1); 2 =  A/sw/Ontario/52156/2003(H1N2); 3 =  A/sw/Ontario/48235/2004(H1N2) and A/sw/Ontario/55383/2004(H1N2); 4 =  A/sw/Minnesota/SG-00239/2007(H1N2); 5 =  A/sw/IL/07003243/2007(H1N2); 6 =  A/sw/Texas/008648/2008(H1N2) and A/sw/Texas/01976/2008(H1N2); and 7 =  a cluster of 21 human H1N2-origin influenza viruses, represented by A/sw/Oklahoma/010226-16/2008(H1N2) (listed in Table S6). The asterisk denotes the first triple reassortant swine influenza viruses identified in the United States in 1998–1999 (e.g., A/sw/Nebraska/209/1998(H3N2)).

**Figure 4:**
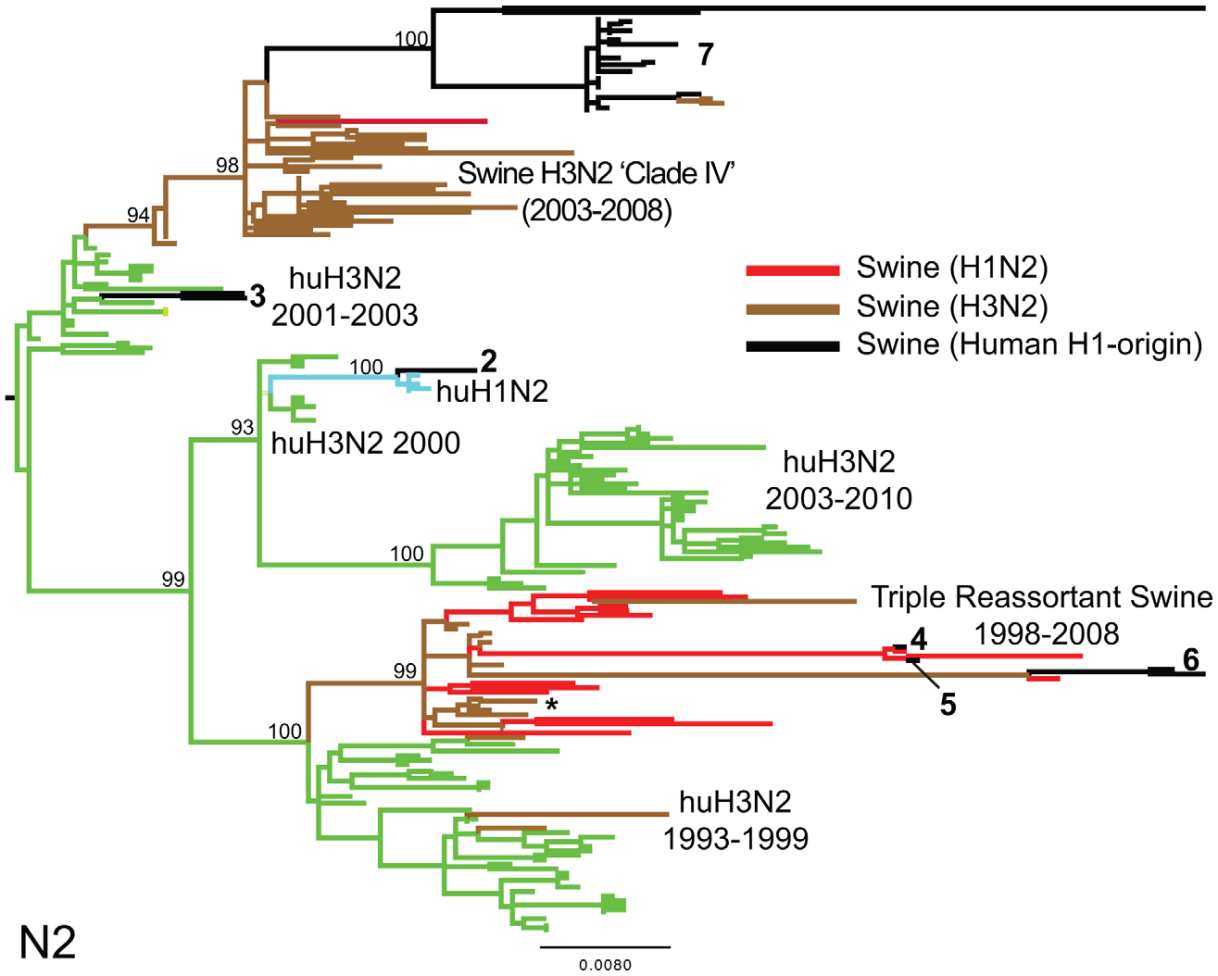
Phylogenetic relationships of the NA (N2) segment. Phylogenetic relationships of the NA (N2) segment (1,407 nt) of 28 human-origin H1 swine influenza viruses, 5 representative human H1N2 influenza viruses, 99 representative H3N2 human influenza viruses, and 60 classical and triple reassortant swine influenza viruses, collected in 1998–2009. Rooting, scaling, labels, and color-coding are identical to Fig. 3, with the addition of ‘Swine H3N2 Clade IV’. Phylogenetic relationships of the N1 sequences (n = 3) contained in cluster 1 are depicted in Fig. S5.

To explore the whole-genome evolution of these human-origin swine influenza viruses, maximum likelihood trees were inferred for the subset (n = 31) of the human-origin swine influenza virus HA1 sequences for which the NA and internal gene sequences were publicly available at GenBank [19]. Major reassortment events are summarized in Table 1 and Fig. 1, including the H1N1 and 2003–2004 H1N2 reassortment events (#1 and #2/3 respectively, Table 1) that have been described previously [12], [13]. The PB2 phylogeny is depicted in Fig. 3, the NA (N2) phylogeny is depicted in Fig. 4, and the phylogenies of other 5 segments and N1 are available in the Supporting Information (Figs. S2, S3, S4, S5, S6, and S7). Notably, all H1N1 and H1N2 isolates collected after 2004 have acquired the triple reassortant internal genes (TRIG) cassette, which were originally derived in 1998 from avian influenza viruses (PB2 and PA), human influenza viruses (PB1), and classical swine influenza viruses (NP, M, and NS). The topology of these trees suggests that the human H1N2-origin lineage may have acquired components of the TRIG cassette approximately 3–4 times over the course of 2007–2008 via multiple reassortment events (Fig. 3, Fig. S2, S3, S4, S5, S6, and S). The largest clade (n = 21) of 2008 human H1N2-origin swine isolates (#7, Table 1) contains the TRIG, but also has acquired via reassortment a human H3N2-origin NA (N2) segment that had circulated in swine at least since 2003, when human H3N2 viruses appear to have reassorted with a lineage of swine A/H3N2 triple reassortant swine viruses that is referred to ‘clade IV’ in the nomenclature for the HA segment [9] (Fig. 4).

**Table 1:** Whole-genome reassortment of human-origin swine influenza viruses.

	PB2	PB1	PA	HA	NP	NA	M	NS
1 Human H1N1-origin swine*	**TRIG**	**TRIG**	**TRIG**	**huH1N1**	**TRIG**	**huH1N1**	**TRIG**	**TRIG**
2 A/sw/Ontario/52156/2003	**huH1N2**	**huH1N2**	**huH1N2**	**huH1N2**	**huH1N2**	**huH1N2**	**huH1N2**	**huH1N2**
3 A/sw/Ontario/48235/2004A/sw/Ontario/55383/2004	**Classical**	**huH3N2**	**Classical**	**huH1N2**	**Classical**	**huH3N2**	**Classical**	**Classical**
4 A/sw/Minnesota/SG-00239/2007	**TRIG**	**TRIG**	**TRIG**	**huH1N2**	**TRIG**	**Triple Reassort**	**TRIG**	**TRIG**
5 A/sw/IL/07003243/2007	**TRIG**	**TRIG**	**TRIG**	**huH1N2**	**TRIG**	**Triple Reassort**	**TRIG**	**TRIG**
6 A/sw/Texas/008648/2008A/sw/Texas/01976/2008	**TRIG**	**TRIG**	**TRIG**	**huH1N2**	**TRIG**	**Triple Reassort**	**TRIG**	**TRIG**
7 A/sw/Oklahoma/010226-16/2008**	**TRIG**	**TRIG**	**TRIG**	**huH1N2**	**TRIG**	**Clade IV**	**TRIG**	**TRIG**

Summary of reassortment events involving 31 human-like swine influenza virus isolates. The phylogenetic position of each isolate or cluster of related isolates is classified for each segment according to nomenclature and color-coding used in Figs. 3 and 4 and Figs. S2, S3, S4, S5, S6, and S7: human H1N1 (huH1N1), human H1N2 (huH1N2), human H3N2 (huH3N2), classical swine influenza viruses (classical), triple reassortant swine viruses (TRIG for internal genes or Triple Reassort for NA), or the clade of triple reassortant swine viruses that acquired a human H3N2-origin N2 (‘Clade IV’, Fig. 4). Reassortment events 1–3 have been described previously [12], [13], and 4–7 are novel in this study.

*represents A/sw/IL/00685/2005(H1N1), A/sw/NC/00573/2005(H1N1), and A/sw/Minnesota/07002083/2007(H1N1)

**represents a cluster of 21 human H1N2-origin influenza viruses with a similar reassortment pattern that are listed in Table S6.

### Spatial movements of human-origin H1 virus in swine

To investigate the spatial dissemination of these novel viruses within the US swine population, we inferred separate Bayesian phylogenies for the H1N1 and H1N2 data sets, considering the three discrete US regions that are well sampled in our data: the Midwest (IL, IN, IA, KS, MI, MN, MO, NE, OH, SD, WI), South-central (OK, TX), and Southeast (NC, SC), which are delineated broadly according to the US farm production regions defined by the USDA [20]. Distinct spatial patterns are clearly evident for both the H1N1 and H1N2 lineages that are depicted in the phylogeny presented in Fig. 2, as all of the H1N1 viruses are from the Southeast (83/138 isolates), mainly representing North Carolina, or the Midwest (55/138 isolates), whereas the H1N2 isolates are predominantly collected in the Midwest (97/169 isolates) and South-central (70/169 isolates) regions (Fig. 2). Both phylogenetic trees exhibit strong spatial structuring, and we observe a statistically significant correlation between phylogeny and location state for the Midwest (p<0.01), South-central (p<0.01), and Southeast (p<0.05) regions on both the H1N1 and H1N2 trees using the parsimony score (PS) and association index (AI) statistics [21].

The maximum clade credibility (MCC) trees annotated with most probable nodal locations indicate multiple introductions of both H1N1 and H1N2 viruses into the Midwest, with the H1N1 virus disseminating Southeast-to-Midwest, and the H1N2 virus disseminating South-central-to-Midwest. In contrast, there is little evidence of viral migration in the opposite directions, or between the South-central and Southeast regions (Fig. 2). ‘Markov jump’ counts [22] of the expected number of location state transitions along the phylogenetic branches provide a quantitative measure of gene flow between regions, representing successful viral introductions from one region to another (Fig. S8). Across the posterior distribution of trees inferred for both subtypes, the vast majority of inter-regional introductions occur in the directions of Southeast-to-Midwest (mean, 13.1) and South-central-to-Midwest (mean, 9.4), with less frequent viral migration also detected from Midwest-to-Southeast (mean, 3.3) (Table 2). Based on the number of swine transported from one region to another over the years of high sampling (2005–2008) (Table S2), we estimate that an introduction of a human-origin H1 swine influenza virus occurs roughly per million swine transported from one region to another (Table 2), although this provides only a lower boundary as the introductions are estimated based on our limited sampling, and we can only detect introductions with substantial onward transmission.

**Table 2:** Viral migration patterns.

	SC-to-SE	SC-to-MW	SE-to-MW	SE-to-SC	MW-to-SC	MW-to-SE
Mean (95% HPD)	0.14 (0, 1)	**9.4** (7, 12)	**13.1** (10, 16)	0 (0, 0)	0.39 (0, 2)	3.3 (2, 6)
Swine-flows	232,596	14,528,536	17,584,512	50,080	275,932	1,086,101
Swine-flows/intro	1,661,400	1,545,589	1,342,329	n/a	707,518	329,121

The number of viral introductions is represented by the Markov jump counts (posterior expected number of location state transitions, with 95% highest posterior density [HPD] intervals) between the three key US regions: South-central (SC), Southeast (SE), and Midwest (MW). The highest estimates of expected location state transitions are highlighted in bold. Using the estimated number of pigs transported between regions (‘swine-flows’) during years that are well sampled in our study (2005–2008) (Table S2), we estimate the number of pigs transported along each inter-regional route per viral introduction.

To quantitatively estimate the importance of known geographical swine population distributions and movements in the spatial dynamics of the virus, we encoded four potential predictors of viral dissemination between pairwise regions as phylogeographic models [23] and fitted these models individually to the sequence data: (i) the number of swine transported annually from one region to another (with directionality), (ii) the swine population size in the region of origin, (iii) the swine population size in the region of destination, and (iv) the product of the swine population sizes in the region of origin and the region of destination (Tables S2 and S3). Given that the South-central, Southeast, and Midwest regions are approximately equidistant from each other by road and geodesic distance, we did not consider geographical distances to be a potential predictor of viral movements in our inter-regional analysis. Bayes factor comparisons [24] via marginal likelihood estimates of the model fit for each potential predictor indicates that the spatial dynamics of the human-origin H1 virus in swine are best described by the number of swine transported annually from one region to another (Table 3). Fixing the rates relative to the swine population size of the region of destination also improved the marginal likelihood, reflecting the directionality of swine-flows from regions of relatively lower swine population size in the South-central and Southeast regions to the largest swine population found in the Midwest. The poorest marginal likelihood was obtained when rates were fixed relative to the swine population in the region of origin, indicating low rates of viral dissemination out of the large swine populations in the Midwest.

**Table 3:** Best-fit phylogeographic model.

Phylogeographic Model	Log marginal likelihood	Log BF
Rates fixed equally	−87.08	–
Rates fixed to population of destination	−83.24	3.8
Rates fixed to population of origin	−108.32	−21.2
Rates fixed to product (destination * origin)	−85.45	1.6
Rates fixed to swine-flows	**−80.99**	**6.1**

Log marginal likelihood estimates for four phylogeographic models [24]. The log Bayes factor (BF) comparison between each possible model and a model with rates fixed equally is provided, with the best-fit model highlighted in bold.

Finally, to ensure that the observed geographical patterns were not an artifact of sampling (Fig. S9), we repeated the phylogeographic analysis using a balanced data set that was randomly subsampled from the original data to obtain equal numbers of sequences from each region (n = 70). Using this balanced data set we find very similar patterns as those derived from the full data set, with substantial viral movement from South-central to Midwest and Southeast to Midwest and strongest support for the ‘swine-flows’ model (Tables S4 and S5). The numbers of viral introductions are somewhat lower than in the original analysis (Table S4) and there is weaker support for the ‘swine-flows’ model (Table S5), but this is expected given the smaller number of sequences used in the sensitivity analysis.

## Discussion

To capture the early spatial patterns of a newly emergent virus in swine populations prior to extensive geographical mixing, this study focused on an H1 influenza virus that was introduced twice from humans into swine around 2003. The fact that this human H1 virus was introduced into swine on two separate occasions (H1N1 and H1N2) allows, uniquely, a side-by-side comparison of the spatial dynamics of two similar emergent viruses. In our statistical analysis, we also take advantage of the independent nature of these two introductions through a model that simultaneously draws information from the H1N1 and H1N2 evolutionary histories to inform the rates of movement in an asymmetric diffusion model. The latter allows us to fully characterize the bidirectional movement between the three major sampling regions despite the fact that the independent lineages provide very different numbers of samples from these regions.

We find that the key source population of the human-origin H1N1 virus is likely to be swine in the Southeastern US, particularly North Carolina, whereas the source population of the H1N2 virus appears to be swine in the South-central US, including Oklahoma. Subsequently, both the H1N1 and H1N2 virus rapidly disseminated to the Midwestern US, apparently following the main swine transportation routes (‘swine-ways’) to the Midwest, the traditional center of American pig farming, to be fattened on the feed corn produced in the region prior to slaughter. Although the Midwest swine population is >4-fold larger than the Southeast swine population and >12-fold greater than the South-central population, the Midwest effectively serves as an ecological sink for the virus due to its commercial function as a final marketing destination and net importer of pigs. These results appear to be robust to sampling bias, as we found similar patterns of viral migration using a subsampled data set comprising 70 isolates that were randomly sampled from each of the three US regions (Tables S4 and S5).

It is certainly possible for novel lineages of influenza virus to begin their spread in the Midwest, and we have not considered farm density, climatic conditions, husbandry practices, biosecurity, vaccination status, or any other factors that would favor viral emergence in the South-central or Southeast versus the Midwest. The role of newer high-density swine production facilities in Oklahoma and North Carolina in viral evolution, in tandem with other immunological or environmental factors, clearly requires study at a finer spatial scale. Rather, our findings suggest that any viral lineage that originates in the Midwest would be less likely to spread to other US regions due to lower rates of regional exportation of Midwestern swine, whereas viruses that originate in the South-central or Southeast are likely to rapidly disseminate to the Midwest.

Although the Midwest does not appear to be a source population for swine influenza viruses, the region is likely to provide a reservoir for multiple genetically distinct variants to co-circulate and exchange segments via reassortment due to the continual importation of swine influenza viruses from other regions. Even a limited sampling (31 whole-genome sequences) revealed extensive reassortment between the human-origin swine viruses and other swine and human influenza viruses over a 7-year period. Both the human H1N1- and H1N2-origin swine viral genomes exhibit a pattern of HA and NA segments that are closely related to human viruses, but internal segments related to triple reassortant swine viruses (TRIG), suggesting that such genomic arrangements may be selectively favored (although this clearly requires further study).

Overall, our study captures the effects of at least a decade of large-scale structural changes in the US commercial swine industry on the evolution and spread of one of the most economically important pathogens in US swine. Further understanding of the role of long-distance pig transport in the ecology and evolution of swine influenza viruses may inform targeted surveillance and mitigation strategies in the future, including intensified surveillance in the less sampled Southern regions. While increased genetic and antigenic diversity observed in swine influenza viruses in recent years has stimulated ongoing research into the development of new influenza vaccines for swine, including live-virus and DNA-based approaches [25], identifying key geographical sources of the virus and reservoirs of genetic diversity may direct vaccination strategies in pigs of different age groups and specified localities. Although the patterns of viral dissemination we identify using the human-origin H1 influenza virus as a case study are striking, these findings invite further study into the phylogeography of swine influenza viruses at more precise spatial scales, including within our broadly defined Midwest region, as well as globally.

## Materials and Methods

### Data generation

For this study we newly generated a total of 1,412 HA1 sequences (889 nt) from H1 influenza A viruses collected from swine in the United States and Canada that exhibited respiratory disease during the period 2003–2008 [26] (Table S6). Two of the isolates were swine viruses that were isolated from turkeys: A/turkey/North Carolina/00533/2005 and A/turkey/North Carolina/00536/2005, but these were triple reassortant viruses and not included in the phylogeographic analysis. HA1 gene sequences were obtained either from virus isolates or directly from the originally submitted nasal swab or lung tissue material. To isolate viruses, the swab or tissue supernatant (in 400-µl amounts) was inoculated on monolayers of MDCK cells grown in 25-cm2 flasks with 5 ml of MEM+ media [27]. All cultures were incubated at 37°C under a 5% CO2 atmosphere. All flasks were examined daily for 7 days under an inverted light microscope to observe virus-induced cytopathic effects (CPE). Viral RNA was extracted from 50 µl of swab supernatant using a magnetic bead procedure (Ambion MagMAX AM1835 and AM1836, Applied Biosystems, Foster City, CA). Segment specific PCR fragments were obtained with One-Step RT-PCR (Qiagen, CA) using influenza A specific primers for HA as described previously [28].

These data were supplemented with 104 additional HA1 sequences from H1 North American swine influenza viruses sampled during 2003–2010 that were downloaded from the National Center for Biotechnology Information (NCBI) Influenza Virus Resource (http://www.ncbi.nlm.nih.gov/genomes/FLU/FLU.html) available at GenBank [19]. This overall total of 1,516 sequences were collected from 23 US states and Canada: Arkansas (AR), Colorado (CO), Georgia (GA), Illinois (IL), Indiana (IN), Iowa (IA), Kansas (KS), Kentucky (KY), Michigan (MI), Minnesota (MN), Missouri (MO), Nebraska (NE), North Carolina (NC), Ohio (OH), Oklahoma (OK), Oregon (OR), Pennsylvania (PA), South Carolina (SC), South Dakota (SD), Tennessee (TN), Texas (TX), Virginia (VA), and Wisconsin (WI). The majority of isolates were collected from the Midwest (n = 921), followed by Southeast (n = 426) and South-central (n = 139) regions (Table S6, Fig. S9). We excluded the possibility that the spatial patterns detected were simply an artifact of uneven sampling during early emergence of the human-like H1 influenza virus in swine (2003–2005) by observing no statistical difference between the number of isolates collected in each region during 2003–2005 compared to 2006 when the virus was widespread in the US (p-value  = 0.9055, Pearson's Chi-square test).

### Phylogenetic analysis

Nucleotide alignments were manually constructed for the HA1 region (889 nt) using the Se-Al program [29]. To infer the evolutionary relationships for the complete data set of 1,516 HA1 sequences, we employed maximum likelihood (ML) methods available through the PhyML program, incorporating a GTR model of nucleotide substitution with gamma-distributed rate variation among sites, and a heuristic SPR branch-swapping search [30]. This phylogenetic analysis identified a cluster of 327 sequences that were separated by a very high number of expected substitutions from the remaining 1,193 swine sequences. To explore the evolutionary origins of these highly divergent sequences in greater detail, a second tree was inferred for the 325 divergent swine sequences (two were excluded due to poor sequence quality) and 92 randomly selected human H1 (HA1) sequences: 3 H1N1 sequences selected from each of the following years: 2000, 2001, 2004, 2005, 2006, 2007, 2008, and 2009; 3 H1N2 sequences selected from 2001; plus an additional 33 H1N1 and 32 H1N2 sequences for the years 2002–2003 during which human-to-swine transmission occurred (the XML file is available in Supplemental Information, Text S1). For this data set, posterior distributions were estimated under a phylogenetic model using a Bayesian Markov chain Monte Carlo (MCMC) method implemented in the BEAST package (v1.6), incorporating the date of sampling [31]. Given the time span of our data set, sequences for which only the year of sampling was known were included and assigned a mid-year sampling date of June 1^st^. Only 30 of 325 isolates did not have an exact date of collection, mainly because collection dates were not available on GenBank [19]; the majority of isolates without exact dates were collected in 2008 in Oklahoma (Table S6). We employed a strict molecular clock, a flexible Bayesian skyline plot (BSP) prior (10 piece-wise constant groups), HKY85 +Γ_4_ model of nucleotide substitution, and the SRD06 codon position model with two partitions for codon positions (1^st^+2^nd^ positions, 3^rd^ position), with substitution model, rate heterogeneity model, and base frequencies unlinked across all codon positions. The MCMC chain was run for 100 million iterations, with sub-sampling every 50,000 iterations. All parameters reached convergence, as assessed visually using Tracer (v.1.5). The initial 10% of the chain was removed as burn-in, and maximum clade credibility (MCC) trees were summarized using TreeAnnotator (v.1.5.4).

A phylogenetic analysis also was conducted upon the 31 human-origin swine influenza viruses (3 H1N1, 28 H1N2) for which whole-genome sequences were available at the NCBI Influenza Virus Resource [19] at GenBank (http://www.ncbi.nlm.nih.gov/genomes/FLU/FLU.html) (Table S6). As the evolutionary relationships of the H1 already had been extensively analyzed (Fig. S1), we downloaded only the remaining 7 genome sequences from GenBank. Due to the divergence of the NA (N1) and NA (N2) sequences, two separate alignments were constructed. In each alignment, 15 representative human influenza viruses collected during 2001–2003 were included, representing the H3N2 (n = 3), H1N2 (n = 5), and H1N1 (n = 7) subtypes. Given the complexity of phylogenetic relationships on the NA (N2) tree arising from frequent reassortment, 99 additional human H3N2 NA sequences were included. Twenty-three swine triple reassortant H3N2 viruses collected during 1998–2009 were included as background. Varying numbers of swine H1N1 influenza virus sequences were available on GenBank for each segment as background: PB2 (n = 38), PB1 (n = 47), PA (n = 36), NP (n = 31), N1 (n = 35), N2 (n = 60), M1/2 (n = 47), NS1/2 (n = 67). Sequence alignments were manually constructed for the major coding regions of PB2 (2,277 nt), PB1 (2,271 nt), PA (2,148 nt), NP (1,494 nt), NA (1,407 nt), M1/2 (979 nt), and NS1/2 (835 nt). Regions of overlapping reading frame were deleted in the case of M1/2 and NS1/2. Here, phylogenetic trees were inferred using the maximum likelihood (ML) method under a GTR+I+Γ_4_ model available in PAUP* [32] for each of these 8 alignments. In all cases TBR branch-swapping was employed to determine the globally optimal tree. To assess the robustness of each node, a bootstrap re-sampling process (1,000 replications) using the neighbor-joining (NJ) method was used, incorporating the ML substitution model. Clades of related isolates were identified by high bootstrap values (>70%) and exceptionally long branch length estimates.

### Spatial analysis

Due to high sampling heterogeneity among US states, we categorized each isolate into three US regions: Midwestern (IL, IN, IA, KS, MI, MN, MO, NE, OH, SD, WI), South-central (OK, TX), and Southeastern (NC, SC). These regions generally correspond to the US farm production regions defined by the US Department of Agriculture (USDA) [20], with the Midwest region including the Corn Belt (IL, IN, IA, MO, OH), Lake States (MI, MN, and WI), and Northern Plains (KS, NE, ND, SD); the Southeast region including Appalachia (KY, TN, NC, VA, WV) and the Southeast (AL, FL, GA, SC); and the South-central region corresponding to the Southern Plains region (OK, TX). Sequences from the other geographic regions that were sampled at relatively low levels were excluded, as were highly phylogenetically divergent sequences that might represent possible sequencing error. This resulted in a final data set of 127 H1N1 and 169 H1N2 isolates that could be used in our detailed spatial analysis. Although we considered separate evolutionary histories for our 127 H1N1 and 169 H1N2 human-like swine HA1 sequences, we jointly inferred the asymmetric rates of movement under a single model of discrete diffusion among the three regions to perform spatial model testing (see below). Moreover, estimating the rates of a single diffusion matrix applied to independent phylogenies may also improve statistical efficiency [23]. Posterior distributions under the Bayesian phylogeographic model [23] were estimated using a MCMC method implemented in BEAST using BEAGLE [33] to improve computational performance. The model incorporated the date of sampling and used a strict molecular clock, BSP prior, and the SRD06 model of nucleotide substitution described. The MCMC chain was run for 100 million iterations, with sub-sampling every 10,000 iterations. All parameters reached convergence, as assessed visually using Tracer (v.1.5). The initial 10% of the chain was removed as burn-in, and MCC trees were summarized using TreeAnnotator (v.1.5.4). The expected number of location state transitions conditional on the observed data was obtained using Markov jump counts [22], [34] again implemented in BEAGLE [33], and summarized per branch and for the complete evolutionary history. *Ad hoc* measures of the extent of geographic structure in the MCC trees were determined for the H1N1 and H1N2 data sets using the parsimony score (PS) and association index (AI) tests as available in the Bayesian Tip-association Significance testing (BaTS) program [21].

### Swine-flows and swine population model-based spatial analysis

To test the importance of swine population sizes and movements in the US in the spatial patterns that were observed, we parameterized the discrete phylogeographic diffusion model in terms of four sources of state-level information on swine populations, aggregated to the regional level and normalized (mean of 1) (Tables S2 and S3). First, we used the number of swine transported annually between states in a pairwise manner for the year 2001, available through the United States Department of Agriculture (USDA) Economic Research Service (http://www.ers.usda.gov/Data/InterstateLivestockMovements/view.asp) (XML file available in the Supplemental Information, Text S2). Second, we obtained data from the USDA 2007 Census of Agriculture [35] to integrate as instantaneous diffusion rates (i) the swine population size of the region of origin (XML file, Text S3), (ii) the swine population size of the region of destination (XML file, Text S4), and (iii) the product of the swine population sizes from the region of origin and the region of destination (XML file, Text S5). Each of these predictors was incorporated into an asymmetric transition matrix that allows for separate directional rates between each pair of locations. A Bayes factor comparison [36] via the relative marginal model likelihoods was used to select the most appropriate model for the data, compared to equal migration rates (XML file, Text S6). Finally, the phylogeographic analysis was repeated using a balanced data set that was randomly subsampled from the original data to obtain equal numbers of sequences from each region (n = 70) (XML file, Text S7) and using independent rate matrices (XML file, Text S8).

### Accession numbers

All sequences were submitted to GenBank and given accession numbers CY040460 – CY082963 (Table S6).

## Supporting Information

Figure S1 Phylogenetic relationships of 1,516 HA1 sequences of H1 swine influenza viruses collected in North America during 2003–2010, inferred using maximum likelihood methods. Branches are color-coded according to the type of isolates at the tips of the tree as follows: green  =  human-origin swine influenza viruses, blue  =  triple reassortant swine influenza viruses, pink  =  pandemic H1N1/09 swine influenza viruses.
(TIF)

Figure S2 Phylogenetic relationships of the PB1 segment. Phylogenetic relationships of the PB1 segment (2,271 nt) of 31 human-origin H1 swine influenza viruses, 15 representative human influenza viruses, and 47 classical and triple reassortant swine influenza viruses, collected during 2000–2010. Rooting, scale, labels, and color-coding are identical to Fig. 3.
(TIF)

Figure S3 Phylogenetic relationships of the PA segment. Phylogenetic relationships of the PA segment (2,148 nt) of 31 human-origin H1 swine influenza viruses, 15 representative human influenza viruses, and 36 classical and triple reassortant swine influenza viruses, collected during 2000–2010. Rooting, scale, labels, and color-coding are identical to Fig. 3.
(TIF)

Figure S4 Phylogenetic relationships of the NP segment. Phylogenetic relationships of the NP segment (1,494 nt) of 31 human-origin H1 swine influenza viruses, 15 representative human influenza viruses, and 31 classical and triple reassortant swine influenza viruses, collected during 2000–2010. Rooting, scaling, labels, and color-coding are identical to Fig. 3.
(TIF)

Figure S5 Phylogenetic relationships of the NA (N1) segment. Phylogenetic relationships of the NA (N1) segment (1,407 nt) of 3 human-origin H1N1 swine influenza viruses (#1, Table 1), 7 representative human H1N1 influenza viruses, and 35 classical and triple reassortant H1N1 swine influenza viruses, collected during 2000–2010. Rooting, scaling, labels, and color-coding are identical to Fig. 3. Phylogenetic relationships of the N2 sequences (n = 28) (#s 2–7, Table 1) are depicted in Fig. 4.
(TIF)

Figure S6 Phylogenetic relationships of the M segment. Phylogenetic relationships of the M segment (979 nt) of 31 human-origin H1 swine influenza viruses, 15 representative human influenza viruses, and 47 classical and triple reassortant swine influenza viruses, collected during 2000–2010. Rooting, scale, labels, and color-coding are identical to Fig. 3.
(TIF)

Figure S7 Phylogenetic relationships of the NS segment. Phylogenetic relationships of the NS segment (835 nt) of 31 human-origin H1 swine influenza viruses, 15 representative human influenza viruses, and 67 classical and triple reassortant swine influenza viruses, collected in 2000–2010. Rooting, scale, labels, and color-coding are identical to Fig. 3.
(TIF)

Figure S8 Time-scaled Bayesian MCC tree of 127 HA1 sequences of human-origin A/H1N1 influenza viruses collected in swine between 2005–2009 (a). Branches are colored according to the most probable location (US region) inferred for the nodes, and the thickness of the branch is proportional to the ‘Markov jump’ counts of location state transitions in the Southeast-to-Midwest direction. Time-scaled Bayesian MCC tree of 169 HA1 sequences of human-origin A/H1N1 influenza viruses collected in swine between 2005–2010 (b). Branches are colored according to the most probable location (US region) inferred for the nodes, and the thickness of the branch is proportional to the ‘Markov jump’ counts of location state transitions in the South-central-to-Midwest direction.
(TIF)

Figure S9 The number of H1 swine influenza virus isolates collected from each of the three US regions – South-central (green), Southeast (red), and Midwest (blue) – and other localities in the US and Canada (purple) during the study period 2003–2010 for: (a) the entire data set of 1,516 isolates and (b) the 325 human-origin H1 swine influenza virus isolates.
(TIF)

Figure S10 Fig. 2, including isolate names.
(EPS)

Table S1 Time to the most recent common ancestor (TMRCA) of human-origin H1N1 and H1N2 influenza viruses in North American swine. Mean TMRCA estimates, with credible intervals, for parent and descendent nodes of the branch along which human-to-swine transmission occurred. Estimated date of emergence in swine is the difference between the TMRCA and the date of the most recently collected isolate for H1N1 (A/Swine/Illinois/03036/2010, June 24, 2010) and H1N2 (A/Swine/Minnesota/03043/2010, July 7, 2010).
(DOCX)

Table S2 ‘Swine-flows’ between US regions, 2001. Number of pigs transported between US regions in a pairwise manner during 2001, aggregated from the state level. Data compiled from State certificates of veterinary inspection for animals for feeding and breeding, and hence reflect general patterns but underestimate actual flows. Available through the United States Department of Agriculture (USDA) Economic Research Service (http://www.ers.usda.gov/Data/InterstateLivestockMovements/view.asp).
(DOCX)

Table S3 US swine populations, 2007. Number of hogs and pigs recorded in each region, aggregated from the state level, in 2007. Data based on inventory and sales, available from the USDA 2007 Census of Agriculture [35].
(DOCX)

Table S4 Comparison of viral migration patterns in the full data set and subset of data with equal sampling in 3 regions. The number of viral introductions is represented by the Markov jump counts (number of expected location state transitions) with 95% highest posterior density (HPD) intervals between the three key US regions: South-central (SC), Southeast (SE), and Midwest (MW), for three data sets: (a) the entire data set of human-origin H1 swine influenza viruses (n = 325 isolates) using combined rate matrices (see Table 2), (b) a subsampled data set including equal numbers (n = 70) of isolates randomly sampled from each region (MW, SC, and SE), and (c) the entire data set using separate rate matrices.
(DOCX)

Table S5 Best-fit phylogeographic model. Log marginal likelihood estimates for four phylogeographic models [24]. The log Bayes factor (BF) comparison between each possible model and a model with rates fixed equally is provided, with the best-fit model highlighted in bold, for both (a) all data (see Table 3) and (b) a subsampled data set including 70 isolates randomly sampled from each region (MW, SC, and SE).
(DOCX)

Table S6 Accession numbers for entire data set (n = 1,516) of swine influenza virus HA1 (H1) sequences used in this analysis, including the 1,412 sequences newly generated in this analysis. GenBank accession numbers (HA), isolate name, and collection date, when available, are listed. The 325 human-origin swine influenza viruses are identified as ‘human’, with the isolates that are associated with cluster 7 denoted in parentheses. The 31 isolates for which whole-genome sequences are available on GenBank are identified with the accession number for the PB2 segment.
(DOCX)

Text S1 Background XML. XML file used to infer Bayesian time-scaled phylogeny for the entire data set of 325 human-origin H1 swine influenza viruses and 92 background human H1N1 and H1N2 influenza viruses (Fig. 2).
(TXT)

Text S2 ‘Swineflows’ XML. XML file used to infer Bayesian time-scaled phylogeny for 127 human H1N1-origin swine influenza viruses and 169 H1N2 human-origin swine influenza viruses, parameterized with data on the number of swine transported annually between US states (Tables 3 and S2).
(TXT)

Text S3 Origins XML. XML file used to infer Bayesian time-scaled phylogeny for 127 human H1N1-origin swine influenza viruses and 169 H1N2 human-origin swine influenza viruses, parameterized with the swine population size of the US region of origin (Tables 3 and S3).
(TXT)

Text S4 Destinations XML. XML file used to infer Bayesian time-scaled phylogeny for 127 human H1N1-origin swine influenza viruses and 169 H1N2 human-origin swine influenza viruses, parameterized the swine population size of the US region of destination (Tables 3 and S3).
(TXT)

Text S5 Gravity XML. XML file used to infer Bayesian time-scaled phylogeny for 127 human H1N1-origin swine influenza viruses and 169 H1N2 human-origin swine influenza viruses, parameterized with the product of the swine population sizes from the region of origin and the region of destination (Tables 3 and S3).
(TXT)

Text S6 Equal rates XML. XML file used to infer Bayesian time-scaled phylogeny for 127 human H1N1-origin swine influenza viruses and 169 H1N2 human-origin swine influenza viruses, with equal diffusion rates (Table 3).
(TXT)

Text S7 Subsample XML. XML file used to infer Bayesian time-scaled phylogeny for a human H1-origin swine influenza virus data set that was randomly subsampled from the original data to obtain equal numbers of sequences from each region (n = 70) (Tables S4 and S5).
(TXT)

Text S8 Independent rates XML. XML file used to infer Bayesian time-scaled phylogeny for 127 human H1N1-origin swine influenza viruses and 169 H1N2 human-origin swine influenza viruses, allowing for independent rate matrices (Tables S4 and S5).
(TXT)
