# Supplementary figures and images for: Spatial Dynamics of Human-Origin H1 Influenza A Virus in North American Swine
Source: PLoS Pathog. 2011 Jun 9;7(6):e1002077. doi: 10.1371/journal.ppat.1002077 (PMC3111536; doi:10.1371/journal.ppat.1002077)

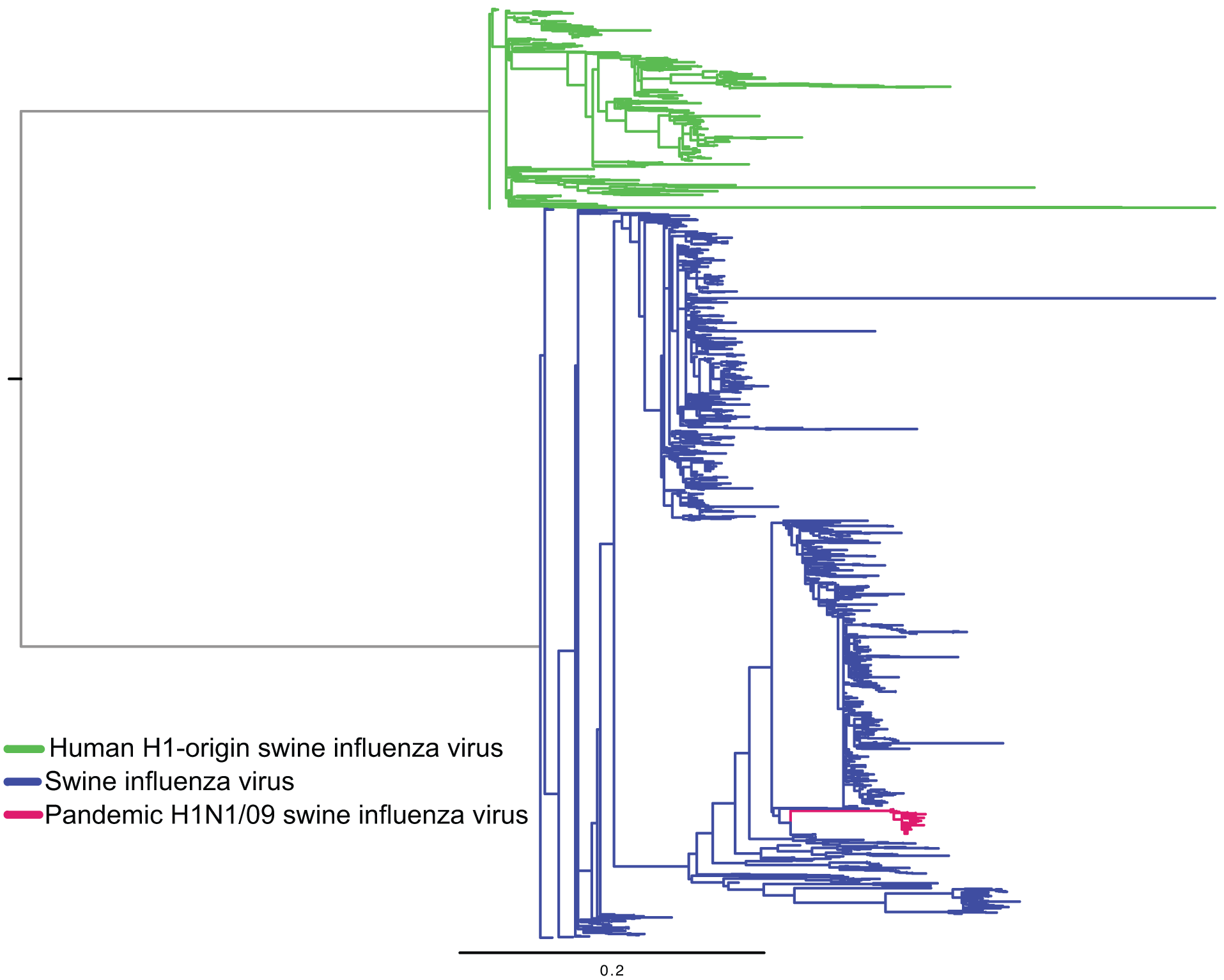

Supplement: Figure S1 — Phylogenetic relationships of 1,516 HA1 sequences of H1 swine influenza viruses collected in North America during 2003–2010, inferred using maximum likelihood methods. Branches are color-coded according to the type of isolates at the tips of the tree as follows: green = human-origin swine influenza viruses, blue = triple reassortant swine influenza viruses, pink = pandemic H1N1/09 swine influenza viruses. (TIF) [file ppat.1002077.s001.tif]

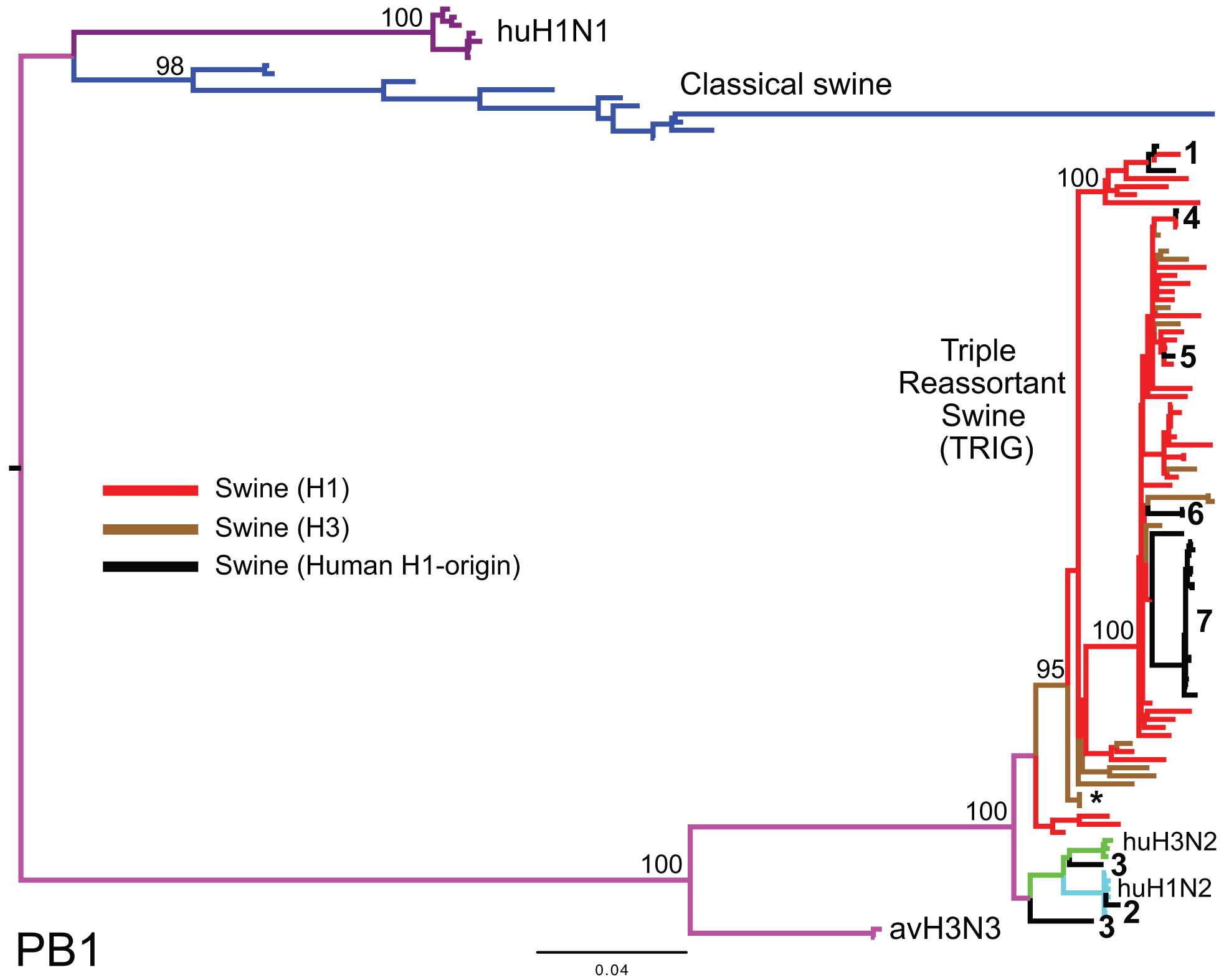

Supplement: Figure S2 — Phylogenetic relationships of the PB1 segment. Phylogenetic relationships of the PB1 segment (2,271 nt) of 31 human-origin H1 swine influenza viruses, 15 representative human influenza viruses, and 47 classical and triple reassortant swine influenza viruses, collected during 2000–2010. Rooting, scale, labels, and color-coding are identical to Fig. 3. (TIF) [file ppat.1002077.s002.tif]

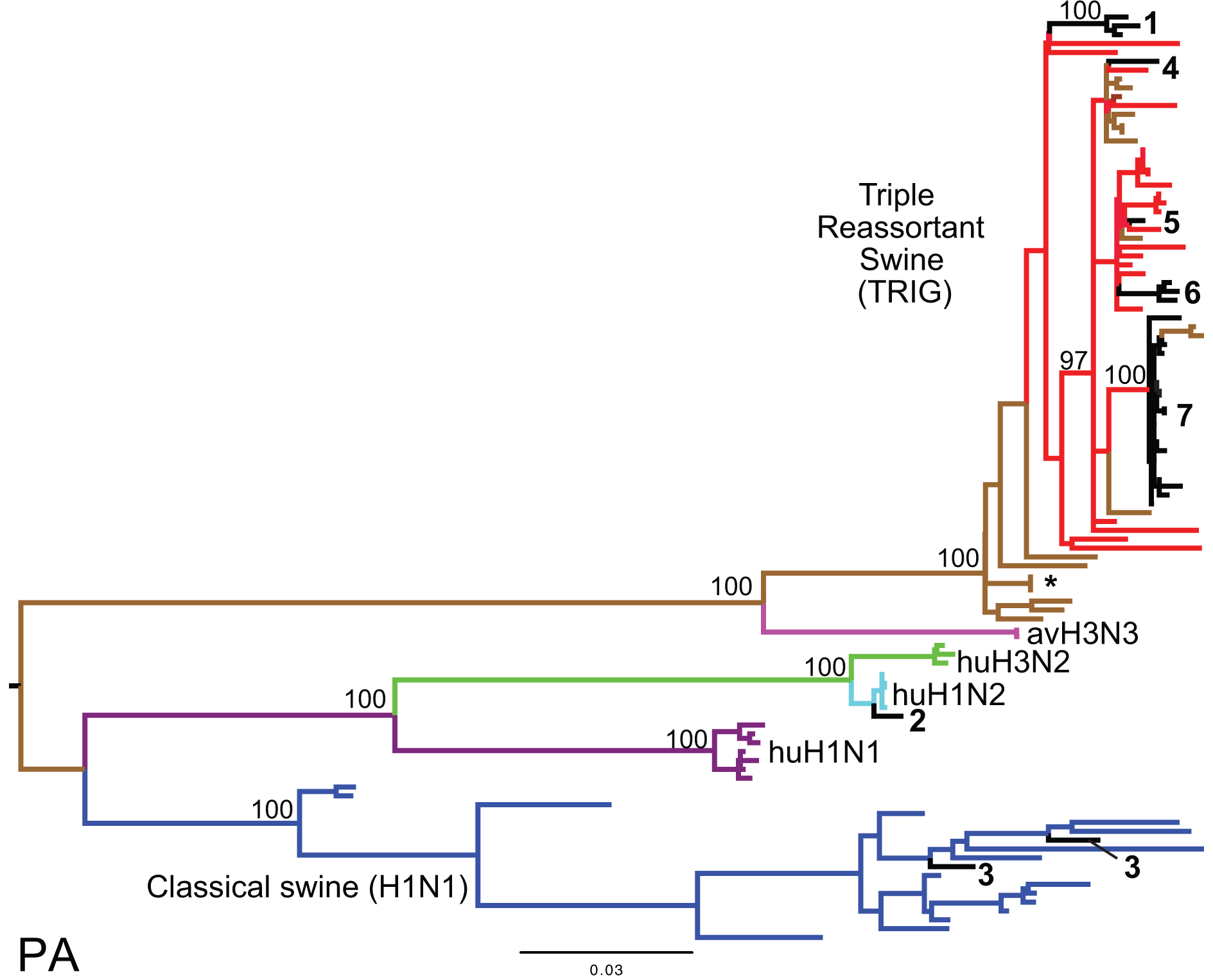

Supplement: Figure S3 — Phylogenetic relationships of the PA segment. Phylogenetic relationships of the PA segment (2,148 nt) of 31 human-origin H1 swine influenza viruses, 15 representative human influenza viruses, and 36 classical and triple reassortant swine influenza viruses, collected during 2000–2010. Rooting, scale, labels, and color-coding are identical to Fig. 3. (TIF) [file ppat.1002077.s003.tif]

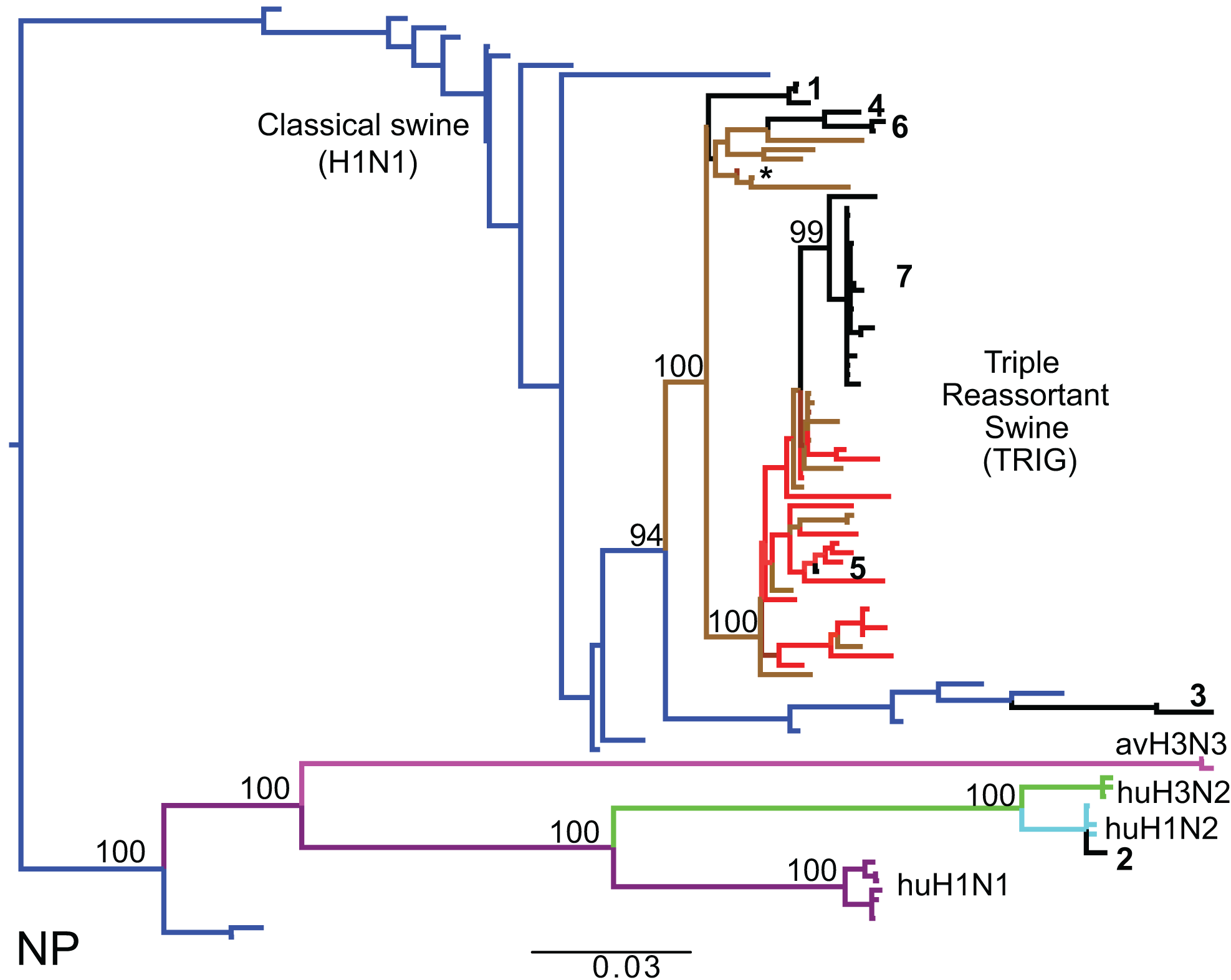

Supplement: Figure S4 — Phylogenetic relationships of the NP segment. Phylogenetic relationships of the NP segment (1,494 nt) of 31 human-origin H1 swine influenza viruses, 15 representative human influenza viruses, and 31 classical and triple reassortant swine influenza viruses, collected during 2000–2010. Rooting, scaling, labels, and color-coding are identical to Fig. 3. (TIF) [file ppat.1002077.s004.tif]

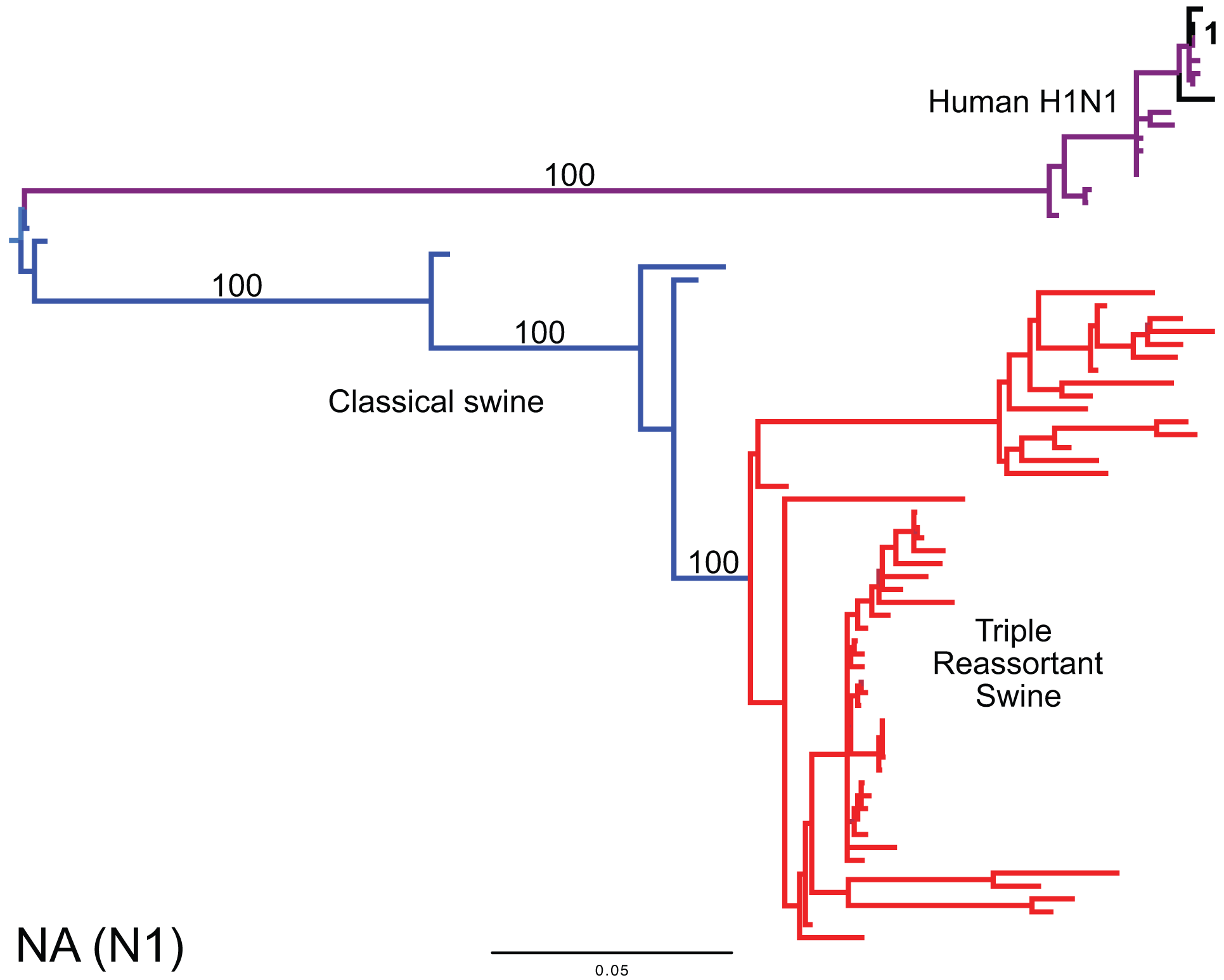

Supplement: Figure S5 — Phylogenetic relationships of the NA (N1) segment. Phylogenetic relationships of the NA (N1) segment (1,407 nt) of 3 human-origin H1N1 swine influenza viruses (#1, Table 1), 7 representative human H1N1 influenza viruses, and 35 classical and triple reassortant H1N1 swine influenza viruses, collected during 2000–2010. Rooting, scaling, labels, and color-coding are identical to Fig. 3. Phylogenetic relationships of the N2 sequences (n = 28) (#s 2–7, Table 1) are depicted in Fig. 4. (TIF) [file ppat.1002077.s005.tif]

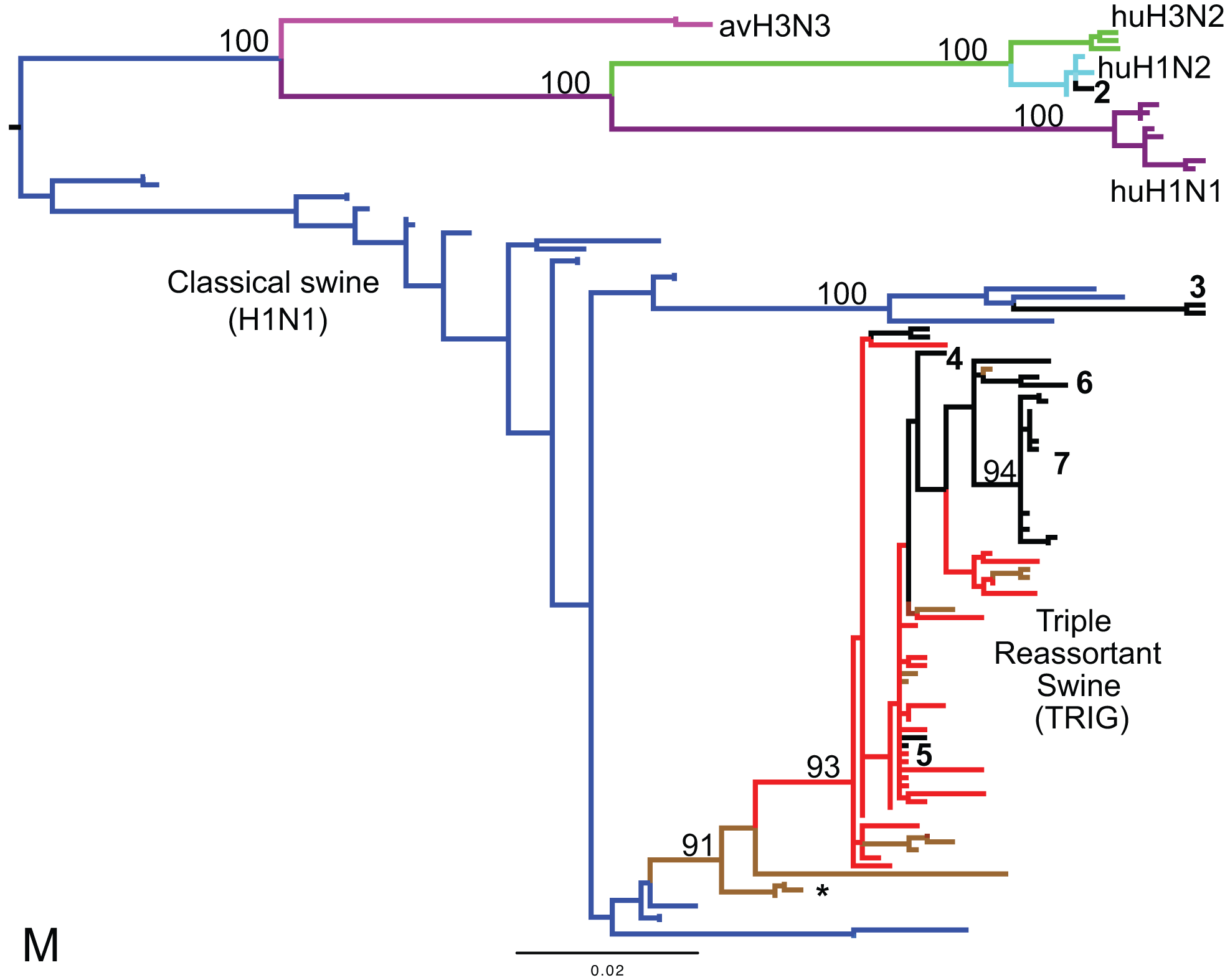

Supplement: Figure S6 — Phylogenetic relationships of the M segment. Phylogenetic relationships of the M segment (979 nt) of 31 human-origin H1 swine influenza viruses, 15 representative human influenza viruses, and 47 classical and triple reassortant swine influenza viruses, collected during 2000–2010. Rooting, scale, labels, and color-coding are identical to Fig. 3. (TIF) [file ppat.1002077.s006.tif]

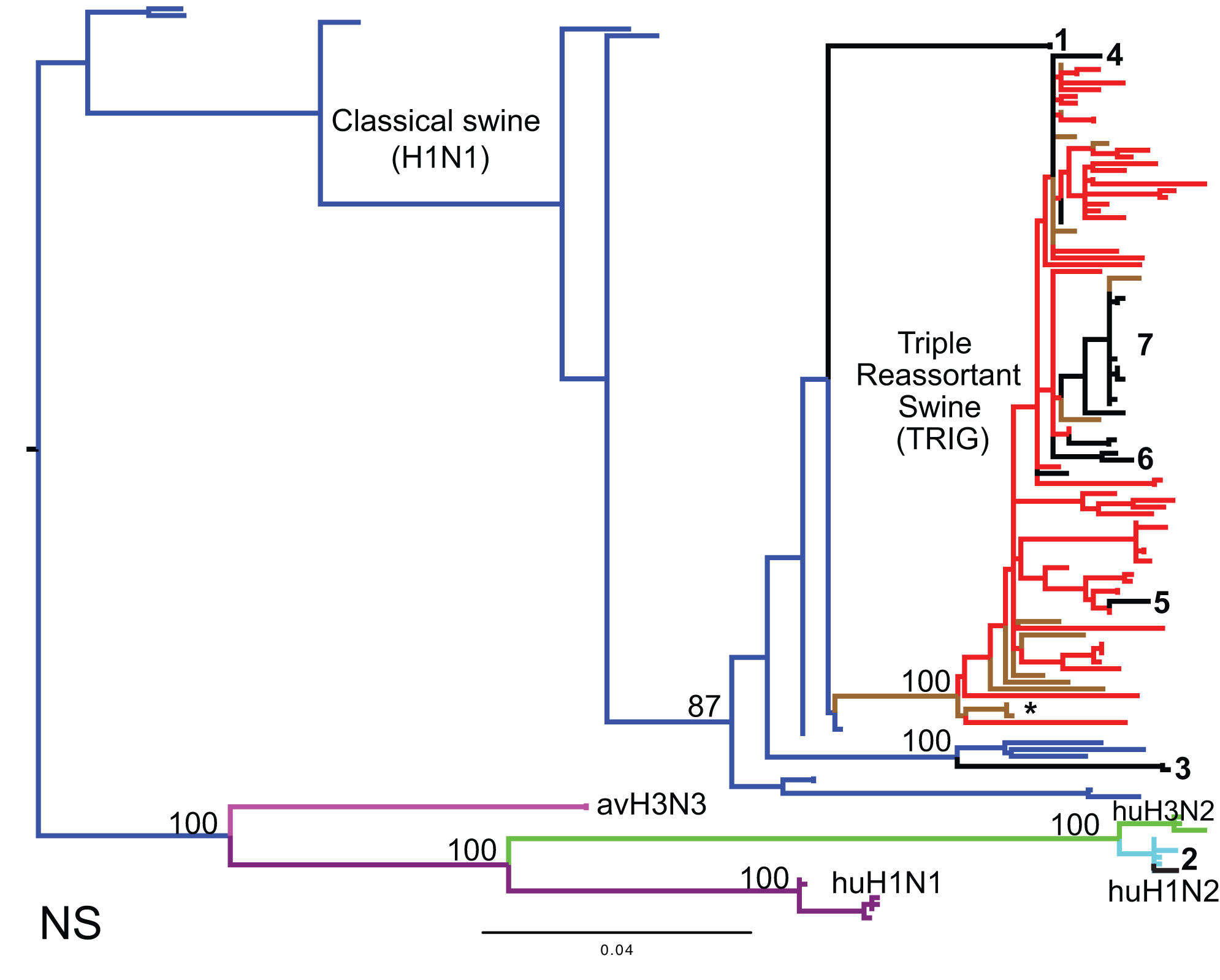

Supplement: Figure S7 — Phylogenetic relationships of the NS segment. Phylogenetic relationships of the NS segment (835 nt) of 31 human-origin H1 swine influenza viruses, 15 representative human influenza viruses, and 67 classical and triple reassortant swine influenza viruses, collected in 2000–2010. Rooting, scale, labels, and color-coding are identical to Fig. 3. (TIF) [file ppat.1002077.s007.tif]

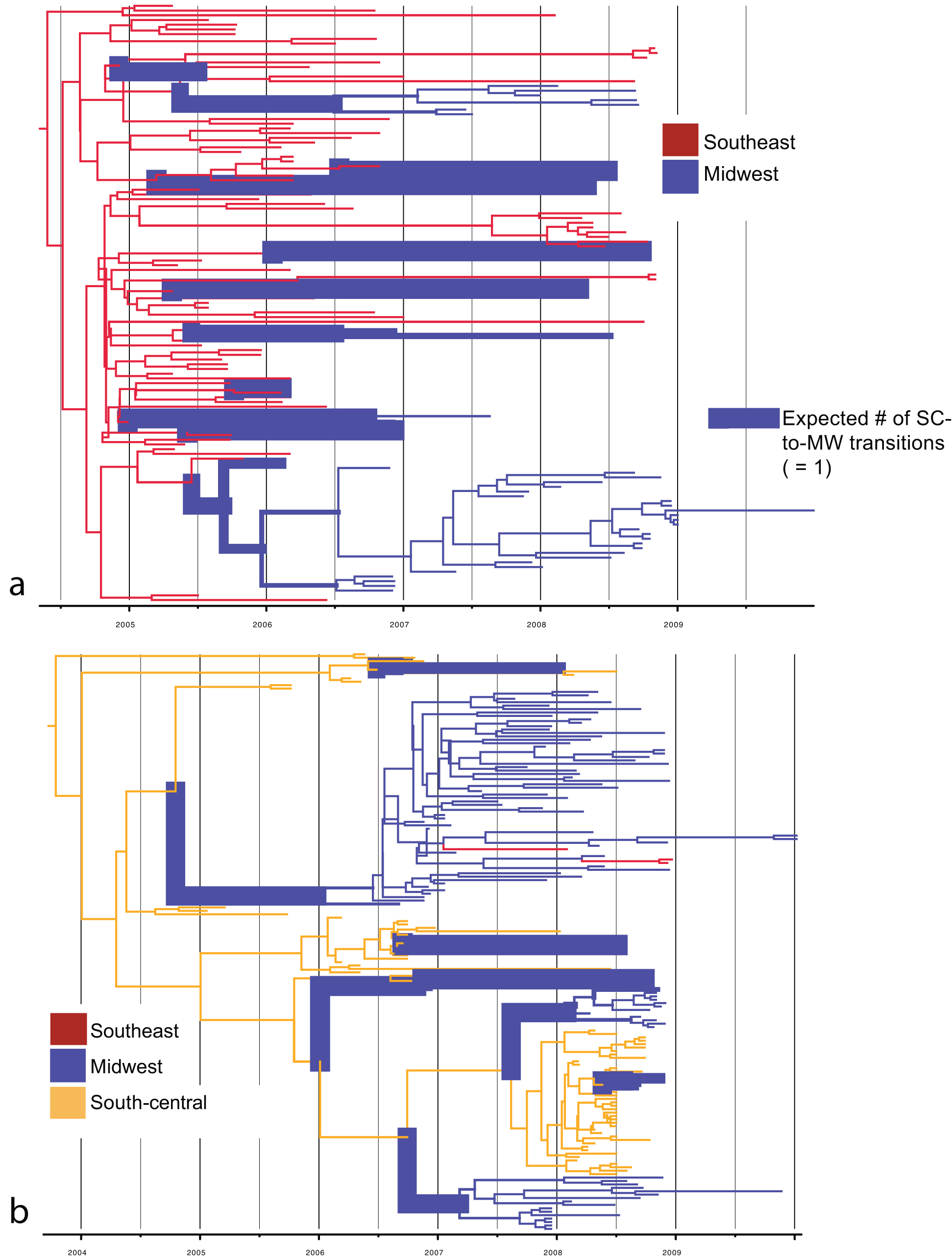

Supplement: Figure S8 — Time-scaled Bayesian MCC tree of 127 HA1 sequences of human-origin A/H1N1 influenza viruses collected in swine between 2005–2009 (a). Branches are colored according to the most probable location (US region) inferred for the nodes, and the thickness of the branch is proportional to the ‘Markov jump’ counts of location state transitions in the Southeast-to-Midwest direction. Time-scaled Bayesian MCC tree of 169 HA1 sequences of human-origin A/H1N1 influenza viruses collected in swine between 2005–2010 (b). Branches are colored according to the most probable location (US region) inferred for the nodes, and the thickness of the branch is proportional to the ‘Markov jump’ counts of location state transitions in the South-central-to-Midwest direction. (TIF) [file ppat.1002077.s008.tif]

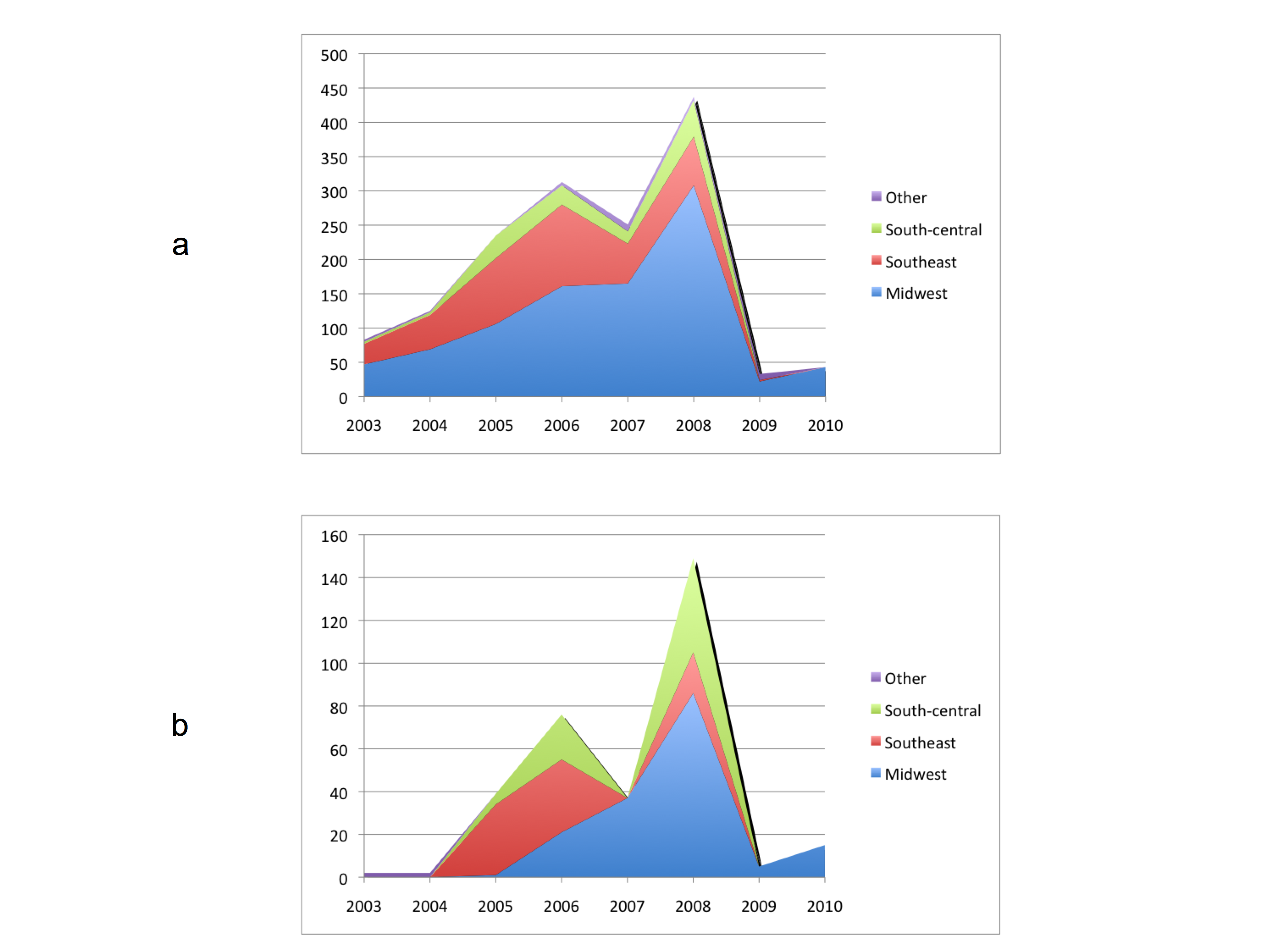

Supplement: Figure S9 — The number of H1 swine influenza virus isolates collected from each of the three US regions – South-central (green), Southeast (red), and Midwest (blue) – and other localities in the US and Canada (purple) during the study period 2003–2010 for: (a) the entire data set of 1,516 isolates and (b) the 325 human-origin H1 swine influenza virus isolates. (TIF) [file ppat.1002077.s009.tif]
