# Supplementary material for: Spatial Dynamics of Human-Origin H1 Influenza A Virus in North American Swine
Source: PLoS Pathog. 2011 Jun 9;7(6):e1002077. doi: 10.1371/journal.ppat.1002077 (PMC3111536; doi:10.1371/journal.ppat.1002077)
Supplement: Table S1 — Time to the most recent common ancestor (TMRCA) of human-origin H1N1 and H1N2 influenza viruses in North American swine. Mean TMRCA estimates, with credible intervals, for parent and descendent nodes of the branch along which human-to-swine transmission occurred. Estimated date of emergence in swine is the difference between the TMRCA and the date of the most recently collected isolate for H1N1 (A/Swine/Illinois/03036/2010, June 24, 2010) and H1N2 (A/Swine/Minnesota/03043/2010, July 7, 2010). (DOCX) [file ppat.1002077.s011.docx]

|  | **TMRCA (mean)** | **95%**  **Credible interval** | **Estimated timeframe of human-to-swine transmission (mean)** | **95%**  **Credible interval** |
| --- | --- | --- | --- | --- |
| **H1N1** | 7.3 – 7.7 yrs | [7.1, 7.5] – [7.5. 7.8] | Oct 2002 – Mar 2003 | [Sept 2002, Dec 2002] – [Jan 2003 – May 2003] |
| **H1N2** | 7.4 – 7.9 yrs | [7.0, 7.7] – [7.6,8.1] | Aug 2002 – Feb 2003 | [May 2002, Nov 2002] – [Oct 2002, Jun 2003] |
