# Supplementary material for: Spatial Dynamics of Human-Origin H1 Influenza A Virus in North American Swine
Source: PLoS Pathog. 2011 Jun 9;7(6):e1002077. doi: 10.1371/journal.ppat.1002077 (PMC3111536; doi:10.1371/journal.ppat.1002077)
Supplement: Table S2 — ‘Swine-flows’ between US regions, 2001. Number of pigs transported between US regions in a pairwise manner during 2001, aggregated from the state level. Data compiled from State certificates of veterinary inspection for animals for feeding and breeding, and hence reflect general patterns but underestimate actual flows. Available through the United States Department of Agriculture (USDA) Economic Research Service (http://www.ers.usda.gov/Data/InterstateLivestockMovements/view.asp). (DOCX) [file ppat.1002077.s012.docx]

|  | **Midwest** | **Southeast** | **South-central** |
| --- | --- | --- | --- |
| Midwest | - | 271,525 | 68,983 |
| Southeast | 4,396,128 | - | 12,520 |
| South-central | 3,632,134 | 58,149 | - |
