# Supplementary material for: Spatial Dynamics of Human-Origin H1 Influenza A Virus in North American Swine
Source: PLoS Pathog. 2011 Jun 9;7(6):e1002077. doi: 10.1371/journal.ppat.1002077 (PMC3111536; doi:10.1371/journal.ppat.1002077)
Supplement: Table S3 — US swine populations, 2007. Number of hogs and pigs recorded in each region, aggregated from the state level, in 2007. Data based on inventory and sales, available from the USDA 2007 Census of Agriculture [35]. (DOCX) [file ppat.1002077.s013.docx]

|  | **Midwest** | **Southeast** | **South-central** |
| --- | --- | --- | --- |
| No. of swine | 48,000,000 | 10,400,000 | 3,500,000 |
