# Supplementary material for: Spatial Dynamics of Human-Origin H1 Influenza A Virus in North American Swine
Source: PLoS Pathog. 2011 Jun 9;7(6):e1002077. doi: 10.1371/journal.ppat.1002077 (PMC3111536; doi:10.1371/journal.ppat.1002077)
Supplement: Table S4 — Comparison of viral migration patterns in the full data set and subset of data with equal sampling in 3 regions. The number of viral introductions is represented by the Markov jump counts (number of expected location state transitions) with 95% highest posterior density (HPD) intervals between the three key US regions: South-central (SC), Southeast (SE), and Midwest (MW), for three data sets: (a) the entire data set of human-origin H1 swine influenza viruses (n = 325 isolates) using combined rate matrices (see Table 2), (b) a subsampled data set including equal numbers (n = 70) of isolates randomly sampled from each region (MW, SC, and SE), and (c) the entire data set using separate rate matrices. (DOCX) [file ppat.1002077.s014.docx]

(a) All data

|  | **SC-to-SE** | **SC-to-MW** | **SE-to-MW** | **SE-to-SC** | **MW-to-SC** | **MW-to-SE** |
| --- | --- | --- | --- | --- | --- | --- |
| Mean (95% HPD) | 0.14 (0, 1) | **9.4** (7, 12) | **13.1** (10, 16) | 0 (0, 0) | 0.39 (0, 2) | 3.3 (2, 6) |
| Swine-flows | 232,596 | 14,528,536 | 17,584,512 | 50,080 | 275,932 | 1,086,101 |
| Swine-flows/intro | 1,661,400 | 1,545,589 | 1,342,329 | n/a | 707,518 | 329,121 |

(b) Subsampled data

|  | **SC-to-SE** | **SC-to-MW** | **SE-to-MW** | **SE-to-SC** | **MW-to-SC** | **MW-to-SE** |
| --- | --- | --- | --- | --- | --- | --- |
| Mean (95% HPD) | 0.08 (0, 1) | **5.1** (3, 7) | **10.4** (9, 13) | 0 (0, 0) | 0.43 (0, 2) | 1.90 (0, 4) |
| Swine-flows | 232,596 | 14,528,536 | 17,584,512 | 50,080 | 275,932 | 1,086,101 |
| Swine-flows/intro | 2,907,450 | 2,848,732 | 1,690818 | n/a | 641,702 | 571,632 |

(c) Separate rate matrices

|  | **SC-to-SE** | **SC-to-MW** | **SE-to-MW** | **SE-to-SC** | **MW-to-SC** | **MW-to-SE** |
| --- | --- | --- | --- | --- | --- | --- |
| Mean (95% HPD) | 0.05 (0, 1) | **9.3** (6, 11) | **12.5** (11, 15) | 0.04 (0, 1) | 0.32 (0, 2) | 2.71 (2, 5) |
| Swine-flows | 232,596 | 14,528,536 | 17,584,512 | 50,080 | 275,932 | 1,086,101 |
| Swine-flows/intro | 4,651,920 | 1,562,208 | 1,406,760 | 1,252,000 | 862,287 | 400,775 |
