# Supplementary material for: Spatial Dynamics of Human-Origin H1 Influenza A Virus in North American Swine
Source: PLoS Pathog. 2011 Jun 9;7(6):e1002077. doi: 10.1371/journal.ppat.1002077 (PMC3111536; doi:10.1371/journal.ppat.1002077)
Supplement: Table S5 — Best-fit phylogeographic model. Log marginal likelihood estimates for four phylogeographic models [24]. The log Bayes factor (BF) comparison between each possible model and a model with rates fixed equally is provided, with the best-fit model highlighted in bold, for both (a) all data (see Table 3) and (b) a subsampled data set including 70 isolates randomly sampled from each region (MW, SC, and SE). (DOCX) [file ppat.1002077.s015.docx]

(a) All data

| **Phylogeographic Model** | **Log marginal likelihood** | **Log BF** |
| --- | --- | --- |
| Rates fixed equally | -87.08 | -- |
| Rates fixed to population of destination | -83.24 | 3.8 |
| Rates fixed to population of origin | -108.32 | -21.2 |
| Rates fixed to product (destination * origin) | -85.45 | 1.6 |
| Rates fixed to swine-flows | **-80.99** | **6.1** |

(b) Subsampled data

| **Phylogeographic Model** | **Log marginal likelihood** | **Log BF** |
| --- | --- | --- |
| Rates fixed equally | -58.62 | -- |
| Rates fixed to population of destination | -57.57 | -0.46 |
| Rates fixed to population of origin | -70.48 | -5.61 |
