# Supplementary material for: Spatial Dynamics of Human-Origin H1 Influenza A Virus in North American Swine
Source: PLoS Pathog. 2011 Jun 9;7(6):e1002077. doi: 10.1371/journal.ppat.1002077 (PMC3111536; doi:10.1371/journal.ppat.1002077)
Supplement: Table S6 — Accession numbers for entire data set (n = 1,516) of swine influenza virus HA1 (H1) sequences used in this analysis, including the 1,412 sequences newly generated in this analysis. GenBank accession numbers (HA), isolate name, and collection date, when available, are listed. The 325 human-origin swine influenza viruses are identified as ‘human’, with the isolates that are associated with cluster 7 denoted in parentheses. The 31 isolates for which whole-genome sequences are available on GenBank are identified with the accession number for the PB2 segment. (DOCX) [file ppat.1002077.s016.docx]

|  | **Accession** | **Isolate name** | | **Date** | **Human-origin** | **Whole-genome** |
| --- | --- | --- | --- | --- | --- | --- |
| 1 | ACT33157 | A/Swine/Alberta/OTH-33-1/2009 | | 5/3/09 |  |  |
| 2 | ACT33158 | A/Swine/Alberta/OTH-33-2/2009 | | 5/3/09 |  |  |
| 3 | ACT33161 | A/Swine/Alberta/OTH-33-21/2009 | | 5/3/09 |  |  |
| 4 | ACT33162 | A/Swine/Alberta/OTH-33-23/2009 | | 5/3/09 |  |  |
| 5 | ACT33163 | A/Swine/Alberta/OTH-33-24/2009 | | 5/3/09 |  |  |
| 6 | ACT33159 | A/Swine/Alberta/OTH-33-3/2009 | | 5/3/09 |  |  |
| 7 | ACT33160 | A/Swine/Alberta/OTH-33-7/2009 | | 5/7/09 |  |  |
| 8 | ACR01025 | A/Swine/Alberta/OTH-33-8/2009 | |  |  |  |
| 9 | CY082070 | A/Swine/Arkansas/00993/2006 | | 2/6/06 |  |  |
| 10 | CY082074 | A/Swine/Arkansas/00998/2006 | | 2/23/06 |  |  |
| 11 | CY082359 | A/Swine/Arkansas/01460/2007 | | 1/31/07 |  |  |
| 12 | [CY040460](http://www.ncbi.nlm.nih.gov/entrez/viewer.fcgi??db=nucleotide&val=CY040460) | A/Swine/Arkansas/63607-3/2008 | | 10/3/08 |  |  |
| 13 | CY081656 | A/Swine/Colorado/00212/2003 | | 11/19/03 |  |  |
| 14 | CY081858 | A/Swine/Colorado/00597/2003 | | 10/23/03 | Human |  |
| 15 | CY082171 | A/Swine/Colorado/01151/2006 | | 5/31/06 |  |  |
| 16 | CY081685 | A/Swine/Georgia/00252/2004 | | 2/24/04 |  |  |
| 17 | CY081707 | A/Swine/Georgia/00297/2004 | | 5/4/04 |  |  |
| 18 | CY082071 | A/Swine/Georgia/00995/2006 | | 2/10/06 |  |  |
| 19 | ACM17254 | A/Swine/IL/07003243/2007 | |  | Human | ACM17250 |
| 20 | ADD64924 | A/Swine/IL/10-001550/2009 | | 12/29/09 |  |  |
| 21 | ADD64928 | A/Swine/IL/10-001551-1/2009 | | 12/20/09 |  |  |
| 22 | ADD64932 | A/Swine/IL/10-001551-2/2009 | | 12/20/09 |  |  |
| 23 | ADG85221 | A/Swine/IL/12660/2010 | | 2/23/10 |  |  |
| 24 | ADG85241 | A/Swine/IL/17315-1/2010 | | 3/18/10 |  |  |
| 25 | ADG85245 | A/Swine/IL/17315-3/2010 | | 3/18/10 |  |  |
| 26 | ADB82958 | A/Swine/IL/32974/2009 | | 11/11/09 |  |  |
| 27 | ADD64916 | A/Swine/IL/35572/2009 | | 12/16/09 |  |  |
| 28 | ADD64920 | A/Swine/IL/35573/2009 | | 12/16/09 |  |  |
| 29 | ADD37828 | A/Swine/IL/3910/2010 | | 1/11/10 |  |  |
| 30 | ADD64936 | A/Swine/IL/5265-1/2010 | | 1/25/10 |  |  |
| 31 | ADD64940 | A/Swine/IL/5265-2/2010 | | 1/25/10 |  |  |
| 32 | CY081619 | A/Swine/Illinois/00124/2003 | | 7/29/03 |  |  |
| 33 | CY081670 | A/Swine/Illinois/00233/2003 | | 12/19/03 |  |  |
| 34 | CY081683 | A/Swine/Illinois/00249/2004 | | 2/18/04 |  |  |
| 35 | CY081687 | A/Swine/Illinois/00255/2004 | | 2/26/04 |  |  |
| 36 | CY081714 | A/Swine/Illinois/00311/2004 | | 6/7/04 |  |  |
| 37 | CY081745 | A/Swine/Illinois/00370/2004 | | 10/12/04 |  |  |
| 38 | CY081771 | A/Swine/Illinois/00426/2004 | | 10/29/04 |  |  |
| 39 | CY081777 | A/Swine/Illinois/00435/2004 | | 12/10/04 |  |  |
| 40 | CY081793 | A/Swine/Illinois/00466/2005 | | 1/5/05 |  |  |
| 41 | CY081803 | A/Swine/Illinois/00493/2005 | | 2/4/05 |  |  |
| 42 | CY081808 | A/Swine/Illinois/00501/2005 | | 2/15/05 |  |  |
| 43 | CY081898 | A/Swine/Illinois/00681/2005 | | 8/3/05 |  |  |
| 44 | CY081899 | A/Swine/Illinois/00685/2005 | | 8/17/05 | Human | ACM17272 |
| 45 | CY081969 | A/Swine/Illinois/00811/2005 | | 10/11/05 |  |  |
| 46 | CY082012 | A/Swine/Illinois/00880/2005 | | 12/1/05 |  |  |
| 47 | CY082016 | A/Swine/Illinois/00886/2005 | | 12/2/05 |  |  |
| 48 | CY082038 | A/Swine/Illinois/00937/2005 | | 12/30/05 |  |  |
| 49 | CY082082 | A/Swine/Illinois/01013/2006 | | 2/28/06 |  |  |
| 50 | CY082083 | A/Swine/Illinois/01014/2006 | | 2/28/06 |  |  |
| 51 | CY082084 | A/Swine/Illinois/01015/2006 | | 2/28/06 |  |  |
| 52 | CY082095 | A/Swine/Illinois/01031/2006 | | 3/2/06 |  |  |
| 53 | CY082097 | A/Swine/Illinois/01035/2006 | | 3/3/06 |  |  |
| 54 | CY082098 | A/Swine/Illinois/01036/2006 | | 3/3/06 |  |  |
| 55 | CY082099 | A/Swine/Illinois/01037/2006 | | 3/3/06 |  |  |
| 56 | CY082100 | A/Swine/Illinois/01038/2006 | | 3/3/06 |  |  |
| 57 | CY082169 | A/Swine/Illinois/01149/2006 | | 5/25/06 |  |  |
| 58 | CY082245 | A/Swine/Illinois/01266/2006 | | 10/13/06 |  |  |
| 59 | CY082298 | A/Swine/Illinois/01353/2006 | | 11/22/06 | Human |  |
| 60 | CY082301 | A/Swine/Illinois/01356/2006 | | 12/5/06 | Human |  |
| 61 | CY082306 | A/Swine/Illinois/01361/2006 | | 11/30/06 | Human |  |
| 62 | CY082313 | A/Swine/Illinois/01372/2006 | | 11/30/06 | Human |  |
| 63 | CY082315 | A/Swine/Illinois/01374/2006 | | 12/5/06 | Human |  |
| 64 | CY082343 | A/Swine/Illinois/01432/2007 | | 1/9/07 |  |  |
| 65 | CY082355 | A/Swine/Illinois/01455/2007 | | 1/24/07 |  |  |
| 66 | CY082361 | A/Swine/Illinois/01462/2007 | | 2/20/07 |  |  |
| 67 | CY082376 | A/Swine/Illinois/01521/2007 | | 3/13/07 |  |  |
| 68 | CY082379 | A/Swine/Illinois/01524/2007 | | 3/15/07 |  |  |
| 69 | CY082393 | A/Swine/Illinois/01568/2007 | | 4/10/07 |  |  |
| 70 | CY082402 | A/Swine/Illinois/01595/2007 | | 5/1/07 |  |  |
| 71 | CY082413 | A/Swine/Illinois/01621/2007 | | 5/15/07 |  |  |
| 72 | CY082415 | A/Swine/Illinois/01626/2007 | | 5/10/07 |  |  |
| 73 | CY082430 | A/Swine/Illinois/01670/2007 | | 6/27/07 |  |  |
| 74 | CY082437 | A/Swine/Illinois/01682/2007 | | 7/12/07 |  |  |
| 75 | CY082446 | A/Swine/Illinois/01698/2007 | | 7/31/07 |  |  |
| 76 | CY082447 | A/Swine/Illinois/01702/2007 | | 8/2/07 |  |  |
| 77 | CY082457 | A/Swine/Illinois/01730/2007 | | 8/24/07 |  |  |
| 78 | CY082464 | A/Swine/Illinois/01746/2007 | | 8/28/07 |  |  |
| 79 | CY082470 | A/Swine/Illinois/01764/2007 | | 8/31/07 |  |  |
| 80 | CY082478 | A/Swine/Illinois/01790/2007 | | 10/5/07 |  |  |
| 81 | CY082487 | A/Swine/Illinois/01812/2007 | | 10/5/07 |  |  |
| 82 | CY082496 | A/Swine/Illinois/01827/2007 | | 11/6/07 |  |  |
| 83 | CY082534 | A/Swine/Illinois/01879/2007 | | 11/30/07 |  |  |
| 84 | CY082541 | A/Swine/Illinois/01887/2007 | | 11/20/07 |  |  |
| 85 | CY082559 | A/Swine/Illinois/01917/2007 | | 12/7/07 |  |  |
| 86 | CY082560 | A/Swine/Illinois/01918/2007 | | 12/7/07 |  |  |
| 87 | CY082561 | A/Swine/Illinois/01919/2007 | | 12/7/07 |  |  |
| 88 | CY082583 | A/Swine/Illinois/01955/2007 | | 12/14/07 |  |  |
| 89 | CY042320 | A/Swine/Illinois/01981/2008 | | 1/16/08 |  |  |
| 90 | CY082616 | A/Swine/Illinois/02006/2008 | | 2/6/08 |  |  |
| 91 | CY082619 | A/Swine/Illinois/02010/2008 | | 1/10/08 | Human |  |
| 92 | CY082644 | A/Swine/Illinois/02044/2008 | | 2/22/08 | Human |  |
| 93 | CY082646 | A/Swine/Illinois/02047/2008 | | 2/26/08 |  |  |
| 94 | CY082648 | A/Swine/Illinois/02049/2008 | | 2/27/08 |  |  |
| 95 | CY082649 | A/Swine/Illinois/02050/2008 | | 2/27/08 |  |  |
| 96 | CY082659 | A/Swine/Illinois/02064/2008 | | 3/12/08 |  |  |
| 97 | CY082660 | A/Swine/Illinois/02065/2008 | | 3/12/08 |  |  |
| 98 | CY082661 | A/Swine/Illinois/02066/2008 | | 3/13/08 | Human |  |
| 99 | CY082674 | A/Swine/Illinois/02087/2008 | | 3/21/08 |  |  |
| 100 | CY082692 | A/Swine/Illinois/02109/2008 | | 4/3/08 |  |  |
| 101 | CY082712 | A/Swine/Illinois/02136/2008 | | 4/23/08 |  |  |
| 102 | CY082716 | A/Swine/Illinois/02144/2008 | | 4/24/08 |  |  |
| 103 | CY082727 | A/Swine/Illinois/02166/2008 | | 5/1/08 |  |  |
| 104 | CY082728 | A/Swine/Illinois/02167/2008 | | 5/8/08 |  |  |
| 105 | CY082754 | A/Swine/Illinois/02217/2008 | | 6/19/08 |  |  |
| 106 | CY042312 | A/Swine/Illinois/02238/2008 | | 7/16/08 |  |  |
| 107 | CY042316 | A/Swine/Illinois/02251/2008 | | 7/31/08 |  |  |
| 108 | CY082778 | A/Swine/Illinois/02260/2008 | | 8/12/08 |  |  |
| 109 | CY082783 | A/Swine/Illinois/02268/2008 | | 8/8/08 | Human |  |
| 110 | CY082803 | A/Swine/Illinois/02296/2008 | | 8/29/08 |  |  |
| 111 | CY082816 | A/Swine/Illinois/02317/2008 | | 9/25/08 |  |  |
| 112 | CY082819 | A/Swine/Illinois/02320/2008 | | 9/26/08 |  |  |
| 113 | CY082824 | A/Swine/Illinois/02329/2008 | | 9/19/08 | Human |  |
| 114 | CY082825 | A/Swine/Illinois/02331/2008 | | 9/24/08 |  |  |
| 115 | CY082834 | A/Swine/Illinois/02346/2008 | | 10/2/08 |  |  |
| 116 | CY082835 | A/Swine/Illinois/02347/2008 | | 10/2/08 |  |  |
| 117 | CY082854 | A/Swine/Illinois/02374/2008 | | 10/8/08 |  |  |
| 118 | CY082858 | A/Swine/Illinois/02380/2008 | | 10/15/08 |  |  |
| 119 | CY082862 | A/Swine/Illinois/02384/2008 | | 10/16/08 |  |  |
| 120 | CY082863 | A/Swine/Illinois/02385/2008 | | 10/17/08 |  |  |
| 121 | CY082893 | A/Swine/Illinois/02431/2008 | | 11/5/08 |  |  |
| 122 | CY082894 | A/Swine/Illinois/02432/2008 | | 11/11/08 |  |  |
| 123 | CY082897 | A/Swine/Illinois/02435/2008 | | 11/13/08 |  |  |
| 124 | CY082900 | A/Swine/Illinois/02443/2008 | | 11/5/08 | Human |  |
| 125 | CY082902 | A/Swine/Illinois/02445/2008 | | 11/11/08 |  |  |
| 126 | CY082907 | A/Swine/Illinois/02450/2008 | | 11/19/08 |  |  |
| 127 | CY082908 | A/Swine/Illinois/02451/2008 | | 11/20/08 |  |  |
| 128 | CY082911 | A/Swine/Illinois/02454/2008 | | 11/21/08 |  |  |
| 129 | CY082912 | A/Swine/Illinois/02455/2008 | | 11/24/08 |  |  |
| 130 | CY082935 | A/Swine/Illinois/02486/2008 | | 12/11/08 | Human |  |
| 131 | CY082936 | A/Swine/Illinois/02487/2008 | | 12/12/08 | Human |  |
| 132 | ADB56963 | A/Swine/Illinois/02919/2009 | | 11/12/09 |  |  |
| 133 | ADB45160 | A/Swine/Illinois/02930/2009 | | 12/30/09 | Human |  |
| 134 | ADB45161 | A/Swine/Illinois/02931/2009 | | 12/30/09 | Human |  |
| 135 | ADB45162 | A/Swine/Illinois/02932/2009 | | 12/30/09 | Human |  |
| 136 | ADC80756 | A/Swine/Illinois/02935/2009 | |  |  |  |
| 137 | ADC80757 | A/Swine/Illinois/02936/2009 | |  |  |  |
| 138 | ADC80758 | A/Swine/Illinois/02937/2009 | |  | Human |  |
| 139 | ADC80759 | A/Swine/Illinois/02938/2009 | |  |  |  |
| 140 | ADC79610 | A/Swine/Illinois/02957/2010 | | 1/26/10 |  |  |
| 141 | ADC79611 | A/Swine/Illinois/02960/2010 | | 1/25/10 |  |  |
| 142 | ADE19044 | A/Swine/Illinois/02984/2010 | | 3/4/10 |  |  |
| 143 | ADJ96330 | A/Swine/Illinois/03033/2010 | | 4/28/10 | Human |  |
| 144 | ADK26592 | A/Swine/Illinois/03036/2010 | | 6/24/10 | Human |  |
| 145 | ADK26593 | A/Swine/Illinois/03037/2010 | | 6/24/10 |  |  |
| 146 | CY082957 | A/Swine/Illinois/16980-2a/2008 | |  |  |  |
| 147 | [CY040461](http://www.ncbi.nlm.nih.gov/entrez/viewer.fcgi??db=nucleotide&val=CY040461) | A/Swine/Illinois/225-1/2008 | | 11/5/08 |  |  |
| 148 | ADG85233 | A/Swine/IN/17311/2010 | | 3/17/10 |  |  |
| 149 | CY081638 | A/Swine/Indiana/00172/2003 | | 4/22/03 |  |  |
| 150 | CY081682 | A/Swine/Indiana/00248/2004 | | 2/17/04 |  |  |
| 151 | CY081772 | A/Swine/Indiana/00427/2004 | | 11/2/04 |  |  |
| 152 | CY081862 | A/Swine/Indiana/00607/2005 | | 5/11/05 |  |  |
| 153 | CY081944 | A/Swine/Indiana/00766/2006 | | 8/18/06 |  |  |
| 154 | CY082005 | A/Swine/Indiana/00870/2005 | | 11/22/05 |  |  |
| 155 | CY082040 | A/Swine/Indiana/00939/2006 | | 9/6/06 |  |  |
| 156 | CY082143 | A/Swine/Indiana/01101/2006 | | 4/20/06 |  |  |
| 157 | CY082212 | A/Swine/Indiana/01224/2006 | | 10/12/06 |  |  |
| 158 | CY082238 | A/Swine/Indiana/01257/2006 | | 10/27/06 |  |  |
| 159 | CY082320 | A/Swine/Indiana/01379/2006 | | 12/6/06 |  |  |
| 160 | CY082414 | A/Swine/Indiana/01622/2007 | | 5/16/07 | Human |  |
| 161 | CY082443 | A/Swine/Indiana/01695/2007 | |  |  |  |
| 162 | CY082545 | A/Swine/Indiana/01893/2007 | | 11/28/07 |  |  |
| 163 | CY082689 | A/Swine/Indiana/02106/2008 | | 3/28/08 |  |  |
| 164 | ADB66685 | A/Swine/Indiana/27007/2009 | |  |  |  |
| 165 | CY081594 | A/Swine/Iowa/00051/2003 | | 2/3/03 |  |  |
| 166 | CY081609 | A/Swine/Iowa/00109/2003 | | 5/20/03 |  |  |
| 167 | CY081623 | A/Swine/Iowa/00129/2003 | | 8/8/03 |  |  |
| 168 | CY081634 | A/Swine/Iowa/00156/2003 | | 9/25/03 |  |  |
| 169 | CY081636 | A/Swine/Iowa/00158/2003 | | 10/8/03 |  |  |
| 170 | CY081646 | A/Swine/Iowa/00193/2003 | | 10/14/03 |  |  |
| 171 | CY081652 | A/Swine/Iowa/00201/2003 | | 10/23/03 |  |  |
| 172 | CY081657 | A/Swine/Iowa/00214/2003 | | 11/19/03 |  |  |
| 173 | CY081658 | A/Swine/Iowa/00215/2003 | | 11/19/03 |  |  |
| 174 | CY081660 | A/Swine/Iowa/00218/2003 | | 10/10/03 |  |  |
| 175 | CY081675 | A/Swine/Iowa/00239/2004 | | 1/7/04 |  |  |
| 176 | CY081684 | A/Swine/Iowa/00250/2004 | | 3/4/04 |  |  |
| 177 | CY081719 | A/Swine/Iowa/00326/2004 | | 6/25/04 |  |  |
| 178 | CY081720 | A/Swine/Iowa/00328/2004 | | 6/25/04 |  |  |
| 179 | CY081756 | A/Swine/Iowa/00391/2004 | | 8/20/04 |  |  |
| 180 | CY081789 | A/Swine/Iowa/00457/2004 | | 12/17/04 |  |  |
| 181 | CY081813 | A/Swine/Iowa/00509/2005 | | 2/1/05 |  |  |
| 182 | CY081814 | A/Swine/Iowa/00510/2005 | | 2/1/05 |  |  |
| 183 | CY081816 | A/Swine/Iowa/00522/2005 | | 3/3/05 |  |  |
| 184 | CY081825 | A/Swine/Iowa/00542/2005 | | 3/18/05 |  |  |
| 185 | CY081826 | A/Swine/Iowa/00543/2005 | | 3/18/05 |  |  |
| 186 | CY081827 | A/Swine/Iowa/00544/2005 | | 3/18/05 |  |  |
| 187 | CY081828 | A/Swine/Iowa/00545/2005 | | 3/18/05 |  |  |
| 188 | CY081829 | A/Swine/Iowa/00549/2005 | | 3/18/05 |  |  |
| 189 | CY081830 | A/Swine/Iowa/00550/2005 | | 3/18/05 |  |  |
| 190 | CY081831 | A/Swine/Iowa/00551/2005 | | 3/18/05 |  |  |
| 191 | CY081832 | A/Swine/Iowa/00552/2005 | | 3/18/05 |  |  |
| 192 | CY081853 | A/Swine/Iowa/00589/2005 | | 4/22/05 |  |  |
| 193 | CY081857 | A/Swine/Iowa/00596/2005 | | 4/20/05 |  |  |
| 194 | CY081861 | A/Swine/Iowa/00605/2005 | | 5/4/05 |  |  |
| 195 | CY081877 | A/Swine/Iowa/00647/2005 | | 6/9/05 |  |  |
| 196 | CY081900 | A/Swine/Iowa/00688/2006 | | 8/17/06 |  |  |
| 197 | CY081903 | A/Swine/Iowa/00694/2006 | | 9/1/06 |  |  |
| 198 | CY081930 | A/Swine/Iowa/00737/2005 | | 9/21/05 |  |  |
| 199 | CY081931 | A/Swine/Iowa/00738/2005 | | 9/21/05 |  |  |
| 200 | CY081938 | A/Swine/Iowa/00748/2005 | | 10/4/05 |  |  |
| 201 | CY081968 | A/Swine/Iowa/00807/2005 | | 10/11/05 |  |  |
| 202 | CY081985 | A/Swine/Iowa/00835/2005 | | 10/25/05 |  |  |
| 203 | CY081989 | A/Swine/Iowa/00842/2005 | | 11/1/05 |  |  |
| 204 | CY081990 | A/Swine/Iowa/00843/2005 | | 11/1/05 |  |  |
| 205 | CY082037 | A/Swine/Iowa/00935/2005 | | 12/23/05 |  |  |
| 206 | CY082062 | A/Swine/Iowa/00980/2006 | | 2/3/06 |  |  |
| 207 | CY082081 | A/Swine/Iowa/01012/2006 | | 2/28/06 |  |  |
| 208 | CY082119 | A/Swine/Iowa/01066/2006 | | 3/23/06 |  |  |
| 209 | CY082133 | A/Swine/Iowa/01087/2006 | | 4/12/06 |  |  |
| 210 | CY082134 | A/Swine/Iowa/01088/2006 | | 4/13/06 |  |  |
| 211 | CY082165 | A/Swine/Iowa/01134/2006 | | 5/10/06 |  |  |
| 212 | CY082183 | A/Swine/Iowa/01175/2006 | | 6/27/06 |  |  |
| 213 | CY082184 | A/Swine/Iowa/01176/2006 | | 6/27/06 |  |  |
| 214 | CY082198 | A/Swine/Iowa/01201/2006 | | 8/16/06 |  |  |
| 215 | CY082204 | A/Swine/Iowa/01210/2006 | | 9/20/06 |  |  |
| 216 | CY082326 | A/Swine/Iowa/01400/2006 | | 12/14/06 |  |  |
| 217 | CY082347 | A/Swine/Iowa/01438/2007 | | 1/19/07 | Human |  |
| 218 | CY082348 | A/Swine/Iowa/01439/2007 | | 1/19/07 | Human |  |
| 219 | CY082349 | A/Swine/Iowa/01440/2007 | | 1/19/07 | Human |  |
| 220 | CY082350 | A/Swine/Iowa/01441/2007 | | 1/19/07 | Human |  |
| 221 | CY082351 | A/Swine/Iowa/01442/2007 | | 1/19/07 |  |  |
| 222 | CY082391 | A/Swine/Iowa/01564/2007 | | 4/25/07 |  |  |
| 223 | CY082461 | A/Swine/Iowa/01739/2007 | | 8/7/07 |  |  |
| 224 | CY082466 | A/Swine/Iowa/01749/2007 | | 8/28/07 |  |  |
| 225 | CY082547 | A/Swine/Iowa/01895/2007 | | 12/12/07 | Human |  |
| 226 | CY082548 | A/Swine/Iowa/01896/2007 | | 12/12/07 | Human |  |
| 227 | CY082549 | A/Swine/Iowa/01897/2007 | | 12/12/07 | Human |  |
| 228 | CY082550 | A/Swine/Iowa/01898/2007 | | 12/12/07 | Human |  |
| 229 | CY082551 | A/Swine/Iowa/01899/2007 | | 12/12/07 | Human |  |
| 230 | CY082552 | A/Swine/Iowa/01900/2007 | | 12/12/07 | Human |  |
| 231 | CY082579 | A/Swine/Iowa/01948/2007 | | 12/21/07 | Human |  |
| 232 | CY082587 | A/Swine/Iowa/01960/2008 | | 1/4/08 | Human |  |
| 233 | CY082588 | A/Swine/Iowa/01961/2008 | | 1/4/08 |  |  |
| 234 | CY082601 | A/Swine/Iowa/01982/2008 | | 1/18/08 |  |  |
| 235 | CY082606 | A/Swine/Iowa/01992/2008 | | 1/25/08 |  |  |
| 236 | CY082623 | A/Swine/Iowa/02014/2008 | | 2/8/08 | Human |  |
| 237 | CY082624 | A/Swine/Iowa/02019/2008 | | 2/15/08 | Human |  |
| 238 | CY082632 | A/Swine/Iowa/02028/2008 | | 2/19/08 |  |  |
| 239 | CY082639 | A/Swine/Iowa/02039/2008 | | 2/13/08 | Human (7) | ADM18132 |
| 240 | CY082651 | A/Swine/Iowa/02054/2008 | | 2/28/08 |  |  |
| 241 | CY082656 | A/Swine/Iowa/02061/2008 | | 3/6/08 |  |  |
| 242 | CY082662 | A/Swine/Iowa/02067/2008 | | 3/14/08 |  |  |
| 243 | CY082663 | A/Swine/Iowa/02068/2008 | | 3/14/08 | Human |  |
| 244 | CY082681 | A/Swine/Iowa/02096/2008 | | 3/28/08 |  |  |
| 245 | CY082682 | A/Swine/Iowa/02097/2008 | | 3/28/08 |  |  |
| 246 | CY082710 | A/Swine/Iowa/02133/2008 | | 4/18/08 | Human |  |
| 247 | CY082719 | A/Swine/Iowa/02152/2008 | | 5/1/08 | Human |  |
| 248 | CY082732 | A/Swine/Iowa/02177/2008 | | 5/22/08 | Human |  |
| 249 | CY082733 | A/Swine/Iowa/02178/2008 | | 5/22/08 | Human |  |
| 250 | CY082742 | A/Swine/Iowa/02193/2008 | | 5/15/08 | Human |  |
| 251 | CY082756 | A/Swine/Iowa/02220/2008 | | 6/27/08 |  |  |
| 252 | CY082763 | A/Swine/Iowa/02229/2008 | | 7/2/08 |  |  |
| 253 | CY082780 | A/Swine/Iowa/02264/2008 | | 8/15/08 |  |  |
| 254 | CY082814 | A/Swine/Iowa/02315/2008 | |  |  |  |
| 255 | CY082822 | A/Swine/Iowa/02325/2008 | | 9/11/08 |  |  |
| 256 | CY082887 | A/Swine/Iowa/02423/2008 | | 10/30/08 |  |  |
| 257 | CY082891 | A/Swine/Iowa/02428/2008 | | 11/10/08 |  |  |
| 258 | CY082916 | A/Swine/Iowa/02460/2008 | | 11/24/08 | Human |  |
| 259 | CY082931 | A/Swine/Iowa/02481/2008 | | 12/5/08 |  |  |
| 260 | CY082933 | A/Swine/Iowa/02484/2008 | | 12/9/08 | Human |  |
| 261 | CY082934 | A/Swine/Iowa/02485/2008 | | 12/9/08 | Human |  |
| 262 | ADG21875 | A/Swine/Iowa/02997/2010 | | 4/11/10 | Human |  |
| 263 | ADG85792 | A/Swine/Iowa/02998/2010 | | 3/17/10 | Human |  |
| 264 | ADG85793 | A/Swine/Iowa/02999/2010 | | 4/1/10 |  |  |
| 265 | ADJ18755 | A/Swine/Iowa/03010/2010 | | 5/4/10 | Human |  |
| 266 | ADJ18756 | A/Swine/Iowa/03026/2010 | | 5/6/10 | Human |  |
| 267 | ADJ38087 | A/Swine/Iowa/03031/2010 | | 5/27/10 |  |  |
| 268 | ADJ38088 | A/Swine/Iowa/03032/2010 | | 6/4/10 |  |  |
| 269 | [CY040466](http://www.ncbi.nlm.nih.gov/entrez/viewer.fcgi??db=nucleotide&val=CY040466) | A/Swine/Iowa/225-8/2008 | |  |  |  |
| 270 | [CY040468](http://www.ncbi.nlm.nih.gov/entrez/viewer.fcgi??db=nucleotide&val=CY040468) | A/Swine/Iowa/63607-1/2008 | | 10/1/08 |  |  |
| 271 | [CY040471](http://www.ncbi.nlm.nih.gov/entrez/viewer.fcgi??db=nucleotide&val=CY040471) | A/Swine/Iowa/63607-23/2008 | | 10/17/08 |  |  |
| 272 | [CY040472](http://www.ncbi.nlm.nih.gov/entrez/viewer.fcgi??db=nucleotide&val=CY040472) | A/Swine/Iowa/63607-34/2008 | | 10/29/08 | Human |  |
| 273 | CY081641 | A/Swine/Kansas/00177/2003 | | 8/27/03 |  |  |
| 274 | CY081680 | A/Swine/Kansas/00246/2004 | | 2/5/04 |  |  |
| 275 | CY081762 | A/Swine/Kansas/00405/2004 | | 9/10/04 |  |  |
| 276 | CY081763 | A/Swine/Kansas/00406/2004 | | 9/10/04 |  |  |
| 277 | CY081764 | A/Swine/Kansas/00407/2004 | | 9/10/04 |  |  |
| 278 | CY081765 | A/Swine/Kansas/00408/2004 | | 9/10/04 |  |  |
| 279 | CY081766 | A/Swine/Kansas/00409/2004 | | 9/10/04 |  |  |
| 280 | CY081776 | A/Swine/Kansas/00434/2004 | | 11/19/04 |  |  |
| 281 | CY081796 | A/Swine/Kansas/00472/2005 | | 1/7/05 |  |  |
| 282 | CY081996 | A/Swine/Kansas/00854/2005 | | 11/11/05 |  |  |
| 283 | CY082046 | A/Swine/Kansas/00948/2006 | | 9/27/06 |  |  |
| 284 | CY082267 | A/Swine/Kansas/01302/2006 | | 11/17/06 |  |  |
| 285 | CY082268 | A/Swine/Kansas/01303/2006 | | 11/17/06 |  |  |
| 286 | CY082270 | A/Swine/Kansas/01305/2006 | | 11/17/06 |  |  |
| 287 | CY082278 | A/Swine/Kansas/01317/2006 | | 11/21/06 | Human |  |
| 288 | CY082342 | A/Swine/Kansas/01430/2007 | | 1/24/07 |  |  |
| 289 | CY082362 | A/Swine/Kansas/01463/2007 | | 2/20/07 |  |  |
| 290 | CY082411 | A/Swine/Kansas/01618/2007 | | 5/18/07 |  |  |
| 291 | CY082419 | A/Swine/Kansas/01640/2007 | | 5/31/07 |  |  |
| 292 | CY082451 | A/Swine/Kansas/01722/2007 | | 8/17/07 |  |  |
| 293 | CY082462 | A/Swine/Kansas/01740/2007 | | 8/15/07 |  |  |
| 294 | CY082473 | A/Swine/Kansas/01771/2007 | | 9/14/07 |  |  |
| 295 | CY082476 | A/Swine/Kansas/01779/2007 | | 9/28/07 |  |  |
| 296 | CY082483 | A/Swine/Kansas/01796/2007 | | 10/12/07 |  |  |
| 297 | CY082484 | A/Swine/Kansas/01797/2007 | | 10/15/07 |  |  |
| 298 | CY082485 | A/Swine/Kansas/01805/2007 | | 10/12/07 |  |  |
| 299 | CY082495 | A/Swine/Kansas/01826/2007 | | 10/31/07 |  |  |
| 300 | CY082499 | A/Swine/Kansas/01830/2007 | | 10/26/07 |  |  |
| 301 | CY082503 | A/Swine/Kansas/01836/2007 | | 10/31/07 |  |  |
| 302 | CY082509 | A/Swine/Kansas/01843/2007 | | 11/14/07 |  |  |
| 303 | CY082538 | A/Swine/Kansas/01883/2007 | | 12/5/07 |  |  |
| 304 | CY082539 | A/Swine/Kansas/01885/2007 | | 12/5/07 |  |  |
| 305 | CY082572 | A/Swine/Kansas/01941/2007 | | 12/13/07 | Human |  |
| 306 | CY082573 | A/Swine/Kansas/01942/2007 | | 12/13/07 |  |  |
| 307 | CY082586 | A/Swine/Kansas/01959/2008 | | 1/4/08 | Human |  |
| 308 | CY082604 | A/Swine/Kansas/01986/2008 | | 1/30/08 | Human |  |
| 309 | CY082611 | A/Swine/Kansas/01999/2008 | | 1/10/08 |  |  |
| 310 | CY082625 | A/Swine/Kansas/02020/2008 | | 2/19/08 |  |  |
| 311 | CY082664 | A/Swine/Kansas/02069/2008 | | 3/14/08 | Human |  |
| 312 | CY082665 | A/Swine/Kansas/02071/2008 | | 3/14/08 |  |  |
| 313 | CY082698 | A/Swine/Kansas/02118/2008 | | 4/7/08 | Human |  |
| 314 | CY082702 | A/Swine/Kansas/02122/2008 | | 4/15/08 |  |  |
| 315 | CY082715 | A/Swine/Kansas/02142/2008 | | 4/24/08 |  |  |
| 316 | CY082798 | A/Swine/Kansas/02291/2008 | | 9/10/08 |  |  |
| 317 | CY082799 | A/Swine/Kansas/02292/2008 | | 9/10/08 |  |  |
| 318 | CY081598 | A/Swine/Kentucky/00057/2003 | | 2/13/03 |  |  |
| 319 | CY081823 | A/Swine/Kentucky/00539/2005 | | 3/11/05 |  |  |
| 320 | CY082185 | A/Swine/Kentucky/01177/2006 | | 6/28/06 | Human |  |
| 321 | CY082394 | A/Swine/Kentucky/01569/2007 | | 3/13/08 | Human |  |
| 322 | CY082673 | A/Swine/Kentucky/02086/2008 | | 4/2/08 |  |  |
| 323 | CY082696 | A/Swine/Kentucky/02116/2008 | |  |  |  |
| 324 | CY082704 | A/Swine/Kentucky/02126/2008 | | 4/2/08 |  |  |
| 325 | CY082794 | A/Swine/Kentucky/02284/2008 | | 8/29/08 |  |  |
| 326 | CY082076 | A/Swine/Manitoba/01004/2006 | |  |  |  |
| 327 | CY082137 | A/Swine/Manitoba/01093/2006 | |  |  |  |
| 328 | CY082186 | A/Swine/Manitoba/01178/2006 | |  |  |  |
| 329 | CY082435 | A/Swine/Manitoba/01680/2007 | | 7/5/07 |  |  |
| 330 | CY082477 | A/Swine/Manitoba/01781/2007 | | 10/2/07 |  |  |
| 331 | CY082521 | A/Swine/Manitoba/01861/2007 | | 11/20/07 |  |  |
| 332 | CY082531 | A/Swine/Manitoba/01875/2007 | | 11/23/07 |  |  |
| 333 | CY082540 | A/Swine/Manitoba/01886/2007 | | 12/6/07 |  |  |
| 334 | CY082542 | A/Swine/Manitoba/01888/2007 | | 11/20/07 |  |  |
| 335 | CY082544 | A/Swine/Manitoba/01891/2007 | | 11/23/07 |  |  |
| 336 | CY082723 | A/Swine/Manitoba/02158/2008 | | 5/13/08 |  |  |
| 337 | CY082736 | A/Swine/Manitoba/02185/2008 | | 5/13/08 |  |  |
| 338 | CY082879 | A/Swine/Manitoba/02414/2008 | | 11/6/08 |  |  |
| 339 | CY082290 | A/Swine/Michigan/01335/2006 | | 12/5/06 |  |  |
| 340 | CY082316 | A/Swine/Michigan/01375/2006 | | 12/5/06 |  |  |
| 341 | CY082317 | A/Swine/Michigan/01376/2006 | | 12/5/06 |  |  |
| 342 | CY082318 | A/Swine/Michigan/01377/2006 | | 12/5/06 |  |  |
| 343 | CY082460 | A/Swine/Michigan/01736/2007 | | 8/16/07 | Human |  |
| 344 | CY082600 | A/Swine/Michigan/01978/2008 | | 1/15/08 | Human |  |
| 345 | CY082878 | A/Swine/Michigan/02413/2008 | | 11/5/08 | Human |  |
| 346 | CY081595 | A/Swine/Minnesota/00053/2003 | | 2/6/03 | Human |  |
| 347 | CY081599 | A/Swine/Minnesota/00058/2003 | | 2/14/03 | Human |  |
| 348 | CY081607 | A/Swine/Minnesota/00105/2003 | | 4/23/03 |  |  |
| 349 | CY081608 | A/Swine/Minnesota/00108/2003 | | 5/6/03 | Human |  |
| 350 | CY081614 | A/Swine/Minnesota/00115/2003 | | 6/26/03 |  |  |
| 351 | CY081617 | A/Swine/Minnesota/00122/2003 | | 7/22/03 |  |  |
| 352 | CY081622 | A/Swine/Minnesota/00128/2003 | | 8/1/03 |  |  |
| 353 | CY081637 | A/Swine/Minnesota/00159/2003 | | 10/8/03 |  |  |
| 354 | CY081645 | A/Swine/Minnesota/00190/2003 | | 10/9/03 |  |  |
| 355 | CY081647 | A/Swine/Minnesota/00194/2003 | | 10/14/03 |  |  |
| 356 | CY081649 | A/Swine/Minnesota/00197/2003 | | 10/16/03 |  |  |
| 357 | CY081650 | A/Swine/Minnesota/00198/2003 | | 10/17/03 |  |  |
| 358 | CY081653 | A/Swine/Minnesota/00206/2003 | | 10/31/03 |  |  |
| 359 | CY081659 | A/Swine/Minnesota/00216/2003 | | 11/20/03 |  |  |
| 360 | CY081668 | A/Swine/Minnesota/00229/2003 | | 12/12/03 |  |  |
| 361 | CY081676 | A/Swine/Minnesota/00241/2004 | | 1/29/04 |  |  |
| 362 | CY081677 | A/Swine/Minnesota/00242/2004 | | 1/29/04 |  |  |
| 363 | CY081681 | A/Swine/Minnesota/00247/2004 | | 2/5/04 |  |  |
| 364 | CY081697 | A/Swine/Minnesota/00272/2004 | | 3/31/04 |  |  |
| 365 | CY081700 | A/Swine/Minnesota/00276/2004 | | 3/31/04 |  |  |
| 366 | CY081703 | A/Swine/Minnesota/00284/2004 | | 4/15/04 |  |  |
| 367 | CY081706 | A/Swine/Minnesota/00292/2004 | | 4/27/04 |  |  |
| 368 | CY081709 | A/Swine/Minnesota/00299/2004 | | 5/10/04 |  |  |
| 369 | CY081710 | A/Swine/Minnesota/00301/2004 | | 5/13/04 |  |  |
| 370 | CY081711 | A/Swine/Minnesota/00302/2004 | | 5/13/04 |  |  |
| 371 | CY081713 | A/Swine/Minnesota/00310/2004 | | 6/4/04 |  |  |
| 372 | CY081724 | A/Swine/Minnesota/00335/2004 | | 5/5/04 |  |  |
| 373 | CY081725 | A/Swine/Minnesota/00336/2004 | | 5/28/04 |  |  |
| 374 | CY081736 | A/Swine/Minnesota/00352/2004 | | 7/23/04 |  |  |
| 375 | CY081743 | A/Swine/Minnesota/00367/2004 | | 10/20/04 |  |  |
| 376 | CY081744 | A/Swine/Minnesota/00369/2004 | | 10/13/04 |  |  |
| 377 | CY081760 | A/Swine/Minnesota/00401/2004 | | 9/9/04 |  |  |
| 378 | CY081778 | A/Swine/Minnesota/00436/2004 | | 12/9/04 |  |  |
| 379 | CY081782 | A/Swine/Minnesota/00440/2004 | | 12/7/04 |  |  |
| 380 | CY081783 | A/Swine/Minnesota/00441/2004 | | 12/7/04 |  |  |
| 381 | CY081784 | A/Swine/Minnesota/00450/2004 | | 11/22/04 |  |  |
| 382 | CY081786 | A/Swine/Minnesota/00452/2004 | | 12/3/04 |  |  |
| 383 | CY081792 | A/Swine/Minnesota/00465/2005 | | 1/4/05 |  |  |
| 384 | CY081795 | A/Swine/Minnesota/00469/2005 | | 1/6/05 |  |  |
| 385 | CY081798 | A/Swine/Minnesota/00480/2005 | | 1/14/05 |  |  |
| 386 | CY081801 | A/Swine/Minnesota/00488/2005 | | 1/26/05 |  |  |
| 387 | CY081806 | A/Swine/Minnesota/00499/2005 | | 2/10/05 |  |  |
| 388 | CY081807 | A/Swine/Minnesota/00500/2005 | | 2/15/05 |  |  |
| 389 | CY081809 | A/Swine/Minnesota/00502/2005 | | 2/16/05 |  |  |
| 390 | CY081824 | A/Swine/Minnesota/00541/2005 | | 3/18/05 |  |  |
| 391 | CY081834 | A/Swine/Minnesota/00555/2005 | | 3/22/05 |  |  |
| 392 | CY081836 | A/Swine/Minnesota/00559/2005 | | 3/25/05 |  |  |
| 393 | CY081837 | A/Swine/Minnesota/00560/2005 | | 3/28/05 |  |  |
| 394 | CY081838 | A/Swine/Minnesota/00561/2005 | | 3/29/05 |  |  |
| 395 | CY081843 | A/Swine/Minnesota/00574/2005 | | 4/26/05 |  |  |
| 396 | CY081844 | A/Swine/Minnesota/00575/2004 | | 11/24/04 |  |  |
| 397 | CY081850 | A/Swine/Minnesota/00584/2005 | | 4/20/05 |  |  |
| 398 | CY081852 | A/Swine/Minnesota/00586/2005 | | 5/3/05 |  |  |
| 399 | CY081854 | A/Swine/Minnesota/00590/2005 | | 3/30/05 |  |  |
| 400 | CY081872 | A/Swine/Minnesota/00626/2005 | | 5/9/05 |  |  |
| 401 | CY081883 | A/Swine/Minnesota/00662/2005 | | 7/1/05 |  |  |
| 402 | CY081889 | A/Swine/Minnesota/00671/2005 | | 7/15/05 |  |  |
| 403 | CY081901 | A/Swine/Minnesota/00691/2006 | | 8/30/06 |  |  |
| 404 | CY081902 | A/Swine/Minnesota/00692/2006 | | 8/30/06 |  |  |
| 405 | CY081904 | A/Swine/Minnesota/00695/2006 | | 9/8/06 |  |  |
| 406 | CY081912 | A/Swine/Minnesota/00711/2005 | | 8/19/05 |  |  |
| 407 | CY081915 | A/Swine/Minnesota/00715/2005 | | 8/25/05 |  |  |
| 408 | CY081918 | A/Swine/Minnesota/00718/2005 | | 8/31/05 |  |  |
| 409 | CY081920 | A/Swine/Minnesota/00720/2005 | | 9/7/05 |  |  |
| 410 | CY081925 | A/Swine/Minnesota/00728/2005 | | 9/14/05 |  |  |
| 411 | CY081945 | A/Swine/Minnesota/00767/2006 | | 8/30/06 |  |  |
| 412 | CY081947 | A/Swine/Minnesota/00773/2005 | | 5/17/05 |  |  |
| 413 | CY081948 | A/Swine/Minnesota/00774/2005 | | 5/18/05 |  |  |
| 414 | CY081951 | A/Swine/Minnesota/00782/2005 | | 9/20/05 |  |  |
| 415 | CY081964 | A/Swine/Minnesota/00798/2005 | | 9/28/05 |  |  |
| 416 | CY081970 | A/Swine/Minnesota/00812/2005 | | 10/11/05 |  |  |
| 417 | CY081974 | A/Swine/Minnesota/00819/2005 | | 10/14/05 |  |  |
| 418 | CY081978 | A/Swine/Minnesota/00825/2005 | | 10/18/05 |  |  |
| 419 | CY081982 | A/Swine/Minnesota/00831/2005 | | 10/24/05 |  |  |
| 420 | CY081987 | A/Swine/Minnesota/00838/2005 | | 10/27/05 |  |  |
| 421 | CY081998 | A/Swine/Minnesota/00857/2005 | | 11/11/05 |  |  |
| 422 | CY082004 | A/Swine/Minnesota/00869/2005 | | 11/18/05 |  |  |
| 423 | CY082010 | A/Swine/Minnesota/00875/2005 | | 11/25/05 |  |  |
| 424 | CY082013 | A/Swine/Minnesota/00881/2005 | | 12/1/05 |  |  |
| 425 | CY082014 | A/Swine/Minnesota/00882/2005 | | 12/2/05 |  |  |
| 426 | CY082017 | A/Swine/Minnesota/00888/2005 | | 12/7/05 |  |  |
| 427 | CY082035 | A/Swine/Minnesota/00930/2005 | | 12/15/05 |  |  |
| 428 | CY082039 | A/Swine/Minnesota/00938/2005 | | 12/30/05 |  |  |
| 429 | CY082041 | A/Swine/Minnesota/00940/2006 | | 9/12/06 |  |  |
| 430 | CY082043 | A/Swine/Minnesota/00944/2006 | | 9/21/06 |  |  |
| 431 | CY082052 | A/Swine/Minnesota/00961/2006 | | 1/12/06 |  |  |
| 432 | CY082053 | A/Swine/Minnesota/00964/2006 | | 1/17/06 |  |  |
| 433 | CY082054 | A/Swine/Minnesota/00965/2006 | | 1/17/06 |  |  |
| 434 | CY082055 | A/Swine/Minnesota/00966/2006 | | 1/17/06 |  |  |
| 435 | CY082072 | A/Swine/Minnesota/00996/2006 | | 2/10/06 |  |  |
| 436 | CY082075 | A/Swine/Minnesota/00999/2006 | | 2/23/06 |  |  |
| 437 | CY082085 | A/Swine/Minnesota/01017/2006 | | 2/28/06 |  |  |
| 438 | CY082114 | A/Swine/Minnesota/01060/2006 | | 3/16/06 |  |  |
| 439 | CY082116 | A/Swine/Minnesota/01062/2006 | | 3/22/06 | Human |  |
| 440 | CY082118 | A/Swine/Minnesota/01065/2006 | | 3/22/06 |  |  |
| 441 | CY082120 | A/Swine/Minnesota/01067/2006 | | 3/23/06 |  |  |
| 442 | CY082121 | A/Swine/Minnesota/01069/2006 | | 3/23/06 |  |  |
| 443 | CY082126 | A/Swine/Minnesota/01075/2006 | | 3/29/06 |  |  |
| 444 | CY082127 | A/Swine/Minnesota/01076/2006 | | 3/31/06 |  |  |
| 445 | CY082131 | A/Swine/Minnesota/01082/2006 | | 4/10/06 |  |  |
| 446 | CY082138 | A/Swine/Minnesota/01094/2006 | | 4/13/06 |  |  |
| 447 | CY082139 | A/Swine/Minnesota/01096/2006 | | 4/13/06 |  |  |
| 448 | CY082140 | A/Swine/Minnesota/01097/2006 | | 4/14/06 |  |  |
| 449 | CY082141 | A/Swine/Minnesota/01099/2006 | | 4/19/06 |  |  |
| 450 | CY082147 | A/Swine/Minnesota/01108/2006 | | 4/24/06 |  |  |
| 451 | CY082149 | A/Swine/Minnesota/01113/2006 | | 4/28/06 |  |  |
| 452 | CY082155 | A/Swine/Minnesota/01120/2006 | | 5/3/06 |  |  |
| 453 | CY082156 | A/Swine/Minnesota/01121/2006 | | 5/3/06 |  |  |
| 454 | CY082159 | A/Swine/Minnesota/01126/2006 | | 5/4/06 |  |  |
| 455 | CY082162 | A/Swine/Minnesota/01130/2006 | | 5/5/06 |  |  |
| 456 | CY082164 | A/Swine/Minnesota/01133/2006 | | 5/10/06 |  |  |
| 457 | CY082166 | A/Swine/Minnesota/01136/2006 | | 5/11/06 |  |  |
| 458 | CY082167 | A/Swine/Minnesota/01137/2006 | | 5/11/06 |  |  |
| 459 | CY082191 | A/Swine/Minnesota/01185/2006 | | 7/12/06 |  |  |
| 460 | CY082195 | A/Swine/Minnesota/01194/2006 | | 8/7/06 |  |  |
| 461 | CY082197 | A/Swine/Minnesota/01200/2006 | | 8/15/06 |  |  |
| 462 | CY082200 | A/Swine/Minnesota/01203/2006 | | 8/16/06 |  |  |
| 463 | CY082202 | A/Swine/Minnesota/01207/2006 | | 8/30/06 |  |  |
| 464 | CY082203 | A/Swine/Minnesota/01209/2006 | | 8/31/06 |  |  |
| 465 | CY082207 | A/Swine/Minnesota/01215/2006 | | 7/18/06 |  |  |
| 466 | CY082208 | A/Swine/Minnesota/01218/2006 | | 10/5/06 |  |  |
| 467 | CY082209 | A/Swine/Minnesota/01219/2006 | | 10/10/06 |  |  |
| 468 | CY082223 | A/Swine/Minnesota/01237/2006 | | 9/28/06 | Human |  |
| 469 | CY082239 | A/Swine/Minnesota/01259/2006 | | 3/9/06 |  |  |
| 470 | CY082240 | A/Swine/Minnesota/01260/2006 | | 3/22/06 |  |  |
| 471 | CY082241 | A/Swine/Minnesota/01261/2006 | | 4/21/06 |  |  |
| 472 | CY082242 | A/Swine/Minnesota/01263/2006 | | 6/13/06 |  |  |
| 473 | CY082246 | A/Swine/Minnesota/01267/2006 | | 10/16/06 |  |  |
| 474 | CY082248 | A/Swine/Minnesota/01270/2006 | | 10/18/06 |  |  |
| 475 | CY082251 | A/Swine/Minnesota/01278/2006 | | 9/27/06 |  |  |
| 476 | CY082252 | A/Swine/Minnesota/01280/2006 | | 10/13/06 |  |  |
| 477 | CY082254 | A/Swine/Minnesota/01283/2006 | | 11/2/06 |  |  |
| 478 | CY082255 | A/Swine/Minnesota/01284/2006 | | 11/3/06 |  |  |
| 479 | CY082257 | A/Swine/Minnesota/01286/2006 | | 11/7/06 |  |  |
| 480 | CY082260 | A/Swine/Minnesota/01291/2006 | | 10/24/06 |  |  |
| 481 | CY082264 | A/Swine/Minnesota/01298/2006 | | 11/3/06 |  |  |
| 482 | CY082265 | A/Swine/Minnesota/01300/2006 | | 11/8/06 |  |  |
| 483 | CY082271 | A/Swine/Minnesota/01306/2006 | | 7/21/06 |  |  |
| 484 | CY082272 | A/Swine/Minnesota/01307/2006 | | 7/25/06 | Human |  |
| 485 | CY082274 | A/Swine/Minnesota/01309/2006 | | 11/8/06 |  |  |
| 486 | CY082275 | A/Swine/Minnesota/01310/2006 | | 11/10/06 |  |  |
| 487 | CY082276 | A/Swine/Minnesota/01311/2006 | | 11/15/06 |  |  |
| 488 | CY082281 | A/Swine/Minnesota/01321/2006 | | 11/15/06 |  |  |
| 489 | CY082286 | A/Swine/Minnesota/01329/2006 | | 11/10/06 |  |  |
| 490 | CY082287 | A/Swine/Minnesota/01331/2006 | | 11/16/06 |  |  |
| 491 | CY082297 | A/Swine/Minnesota/01352/2006 | | 11/16/06 |  |  |
| 492 | CY082300 | A/Swine/Minnesota/01355/2006 | | 12/4/06 |  |  |
| 493 | CY082303 | A/Swine/Minnesota/01358/2006 | | 11/16/06 |  |  |
| 494 | CY082305 | A/Swine/Minnesota/01360/2006 | | 11/29/06 | Human |  |
| 495 | CY082310 | A/Swine/Minnesota/01369/2006 | | 12/1/06 | Human |  |
| 496 | CY082311 | A/Swine/Minnesota/01370/2006 | | 11/16/06 |  |  |
| 497 | CY082312 | A/Swine/Minnesota/01371/2006 | | 11/16/06 |  |  |
| 498 | CY082319 | A/Swine/Minnesota/01378/2006 | | 12/5/06 | Human |  |
| 499 | CY082327 | A/Swine/Minnesota/01401/2006 | | 12/15/06 | Human |  |
| 500 | CY082329 | A/Swine/Minnesota/01403/2006 | | 12/21/06 | Human |  |
| 501 | CY082330 | A/Swine/Minnesota/01405/2006 | | 12/27/06 | Human |  |
| 502 | CY082332 | A/Swine/Minnesota/01411/2006 | | 12/7/06 | Human |  |
| 503 | CY082336 | A/Swine/Minnesota/01415/2006 | | 12/27/06 |  |  |
| 504 | CY082338 | A/Swine/Minnesota/01419/2006 | | 12/28/06 |  |  |
| 505 | CY082341 | A/Swine/Minnesota/01427/2007 | | 1/10/07 |  |  |
| 506 | CY082344 | A/Swine/Minnesota/01433/2007 | | 1/11/07 |  |  |
| 507 | CY082345 | A/Swine/Minnesota/01435/2007 | | 1/17/07 |  |  |
| 508 | CY082346 | A/Swine/Minnesota/01436/2007 | | 1/19/07 |  |  |
| 509 | CY082352 | A/Swine/Minnesota/01445/2007 | | 1/19/07 |  |  |
| 510 | CY082356 | A/Swine/Minnesota/01457/2007 | | 2/8/07 |  |  |
| 511 | CY082357 | A/Swine/Minnesota/01458/2006 | | 11/17/06 |  |  |
| 512 | CY082358 | A/Swine/Minnesota/01459/2007 | | 1/25/07 |  |  |
| 513 | CY082363 | A/Swine/Minnesota/01465/2007 | | 2/14/07 |  |  |
| 514 | CY082364 | A/Swine/Minnesota/01467/2007 | | 2/14/07 | Human |  |
| 515 | CY082380 | A/Swine/Minnesota/01529/2007 | | 3/9/07 |  |  |
| 516 | CY082382 | A/Swine/Minnesota/01537/2007 | | 4/4/07 |  |  |
| 517 | CY082383 | A/Swine/Minnesota/01538/2006 | | 12/20/06 |  |  |
| 518 | CY082384 | A/Swine/Minnesota/01541/2007 | | 3/22/07 |  |  |
| 519 | CY082385 | A/Swine/Minnesota/01547/2007 | | 4/3/07 |  |  |
| 520 | CY082386 | A/Swine/Minnesota/01548/2007 | | 4/5/07 |  |  |
| 521 | CY082387 | A/Swine/Minnesota/01552/2007 | | 4/11/07 |  |  |
| 522 | CY082395 | A/Swine/Minnesota/01570/2007 | | 4/17/07 |  |  |
| 523 | CY082396 | A/Swine/Minnesota/01574/2007 | | 4/25/07 |  |  |
| 524 | CY082399 | A/Swine/Minnesota/01581/2007 | | 4/26/07 |  |  |
| 525 | CY082400 | A/Swine/Minnesota/01584/2007 | | 4/27/07 |  |  |
| 526 | CY082403 | A/Swine/Minnesota/01597/2007 | | 5/9/07 |  |  |
| 527 | CY082404 | A/Swine/Minnesota/01599/2007 | | 5/2/07 |  |  |
| 528 | CY082405 | A/Swine/Minnesota/01600/2007 | | 4/6/07 |  |  |
| 529 | CY082406 | A/Swine/Minnesota/01611/2007 | | 5/4/07 |  |  |
| 530 | CY082407 | A/Swine/Minnesota/01612/2007 | | 5/7/07 |  |  |
| 531 | CY082408 | A/Swine/Minnesota/01613/2007 | | 5/8/07 |  |  |
| 532 | CY082409 | A/Swine/Minnesota/01614/2007 | | 5/8/07 |  |  |
| 533 | CY082412 | A/Swine/Minnesota/01620/2007 | | 5/14/07 |  |  |
| 534 | CY082416 | A/Swine/Minnesota/01632/2007 | | 5/25/07 |  |  |
| 535 | CY082418 | A/Swine/Minnesota/01637/2007 | | 6/6/07 |  |  |
| 536 | CY082420 | A/Swine/Minnesota/01647/2007 | | 6/11/07 |  |  |
| 537 | CY082421 | A/Swine/Minnesota/01650/2007 | | 6/12/07 |  |  |
| 538 | CY082427 | A/Swine/Minnesota/01659/2007 | | 6/27/07 |  |  |
| 539 | CY082429 | A/Swine/Minnesota/01666/2007 | | 6/21/07 |  |  |
| 540 | CY082436 | A/Swine/Minnesota/01681/2007 | | 7/3/07 |  |  |
| 541 | CY082438 | A/Swine/Minnesota/01683/2007 | | 7/12/07 | Human |  |
| 542 | CY082442 | A/Swine/Minnesota/01689/2007 | | 7/12/07 |  |  |
| 543 | CY082448 | A/Swine/Minnesota/01706/2007 | | 8/8/07 |  |  |
| 544 | CY082449 | A/Swine/Minnesota/01708/2007 | | 8/10/07 |  |  |
| 545 | CY082452 | A/Swine/Minnesota/01724/2007 | | 8/21/07 | Human |  |
| 546 | CY082454 | A/Swine/Minnesota/01726/2007 | | 7/12/07 |  |  |
| 547 | CY082455 | A/Swine/Minnesota/01727/2007 | | 8/8/07 |  |  |
| 548 | CY082456 | A/Swine/Minnesota/01728/2007 | | 8/21/07 |  |  |
| 549 | CY082479 | A/Swine/Minnesota/01791/2007 | | 10/8/07 |  |  |
| 550 | CY082480 | A/Swine/Minnesota/01792/2007 | | 10/8/07 |  |  |
| 551 | CY082482 | A/Swine/Minnesota/01795/2007 | | 10/11/07 |  |  |
| 552 | CY082488 | A/Swine/Minnesota/01813/2007 | | 10/11/07 | Human |  |
| 553 | CY082500 | A/Swine/Minnesota/01831/2007 | | 10/26/07 |  |  |
| 554 | CY082501 | A/Swine/Minnesota/01832/2007 | | 10/26/07 |  |  |
| 555 | CY082502 | A/Swine/Minnesota/01835/2007 | | 10/30/07 |  |  |
| 556 | CY082504 | A/Swine/Minnesota/01837/2007 | | 10/31/07 |  |  |
| 557 | CY082505 | A/Swine/Minnesota/01838/2007 | | 10/31/07 |  |  |
| 558 | CY082506 | A/Swine/Minnesota/01839/2007 | | 11/1/07 |  |  |
| 559 | CY082507 | A/Swine/Minnesota/01840/2007 | | 11/1/07 |  |  |
| 560 | CY082508 | A/Swine/Minnesota/01842/2007 | | 11/7/07 |  |  |
| 561 | CY082510 | A/Swine/Minnesota/01844/2007 | | 11/1/07 |  |  |
| 562 | CY082511 | A/Swine/Minnesota/01845/2007 | | 11/1/07 | Human |  |
| 563 | CY082512 | A/Swine/Minnesota/01846/2007 | | 11/2/07 |  |  |
| 564 | CY082515 | A/Swine/Minnesota/01854/2007 | | 11/13/07 |  |  |
| 565 | CY082518 | A/Swine/Minnesota/01857/2007 | | 11/14/07 |  |  |
| 566 | CY082519 | A/Swine/Minnesota/01858/2007 | | 11/15/07 |  |  |
| 567 | CY082520 | A/Swine/Minnesota/01859/2007 | | 11/20/07 | Human |  |
| 568 | CY082522 | A/Swine/Minnesota/01863/2007 | | 11/21/07 | Human |  |
| 569 | CY082523 | A/Swine/Minnesota/01864/2007 | | 11/20/07 |  |  |
| 570 | CY082528 | A/Swine/Minnesota/01872/2007 | | 11/14/07 |  |  |
| 571 | CY082529 | A/Swine/Minnesota/01873/2007 | | 11/15/07 |  |  |
| 572 | CY082530 | A/Swine/Minnesota/01874/2007 | | 11/23/07 |  |  |
| 573 | CY082533 | A/Swine/Minnesota/01878/2007 | | 11/29/07 |  |  |
| 574 | CY082537 | A/Swine/Minnesota/01882/2007 | | 12/5/07 | Human |  |
| 575 | CY082543 | A/Swine/Minnesota/01890/2007 | | 11/23/07 |  |  |
| 576 | CY082553 | A/Swine/Minnesota/01908/2007 | | 11/27/07 |  |  |
| 577 | CY082554 | A/Swine/Minnesota/01909/2007 | | 11/30/07 |  |  |
| 578 | CY082557 | A/Swine/Minnesota/01912/2007 | | 12/5/07 |  |  |
| 579 | CY082558 | A/Swine/Minnesota/01913/2007 | | 12/5/07 | Human |  |
| 580 | CY082570 | A/Swine/Minnesota/01938/2007 | | 11/27/07 | Human |  |
| 581 | CY082574 | A/Swine/Minnesota/01943/2007 | | 12/13/07 |  |  |
| 582 | CY082581 | A/Swine/Minnesota/01952/2007 | | 12/4/07 |  |  |
| 583 | CY082589 | A/Swine/Minnesota/01964/2007 | | 12/13/07 |  |  |
| 584 | CY082591 | A/Swine/Minnesota/01968/2007 | | 12/28/07 | Human |  |
| 585 | CY082592 | A/Swine/Minnesota/01969/2007 | | 12/28/07 |  |  |
| 586 | CY082602 | A/Swine/Minnesota/01983/2008 | | 1/17/08 | Human |  |
| 587 | CY082610 | A/Swine/Minnesota/01998/2008 | | 1/8/08 | Human |  |
| 588 | CY082620 | A/Swine/Minnesota/02011/2008 | | 1/31/08 | Human |  |
| 589 | CY082626 | A/Swine/Minnesota/02021/2008 | | 1/29/08 |  |  |
| 590 | CY082627 | A/Swine/Minnesota/02022/2008 | | 2/1/08 |  |  |
| 591 | CY082633 | A/Swine/Minnesota/02029/2008 | | 2/19/08 |  |  |
| 592 | CY082634 | A/Swine/Minnesota/02031/2008 | | 2/20/08 | Human |  |
| 593 | CY082640 | A/Swine/Minnesota/02040/2008 | | 2/14/08 |  |  |
| 594 | CY082650 | A/Swine/Minnesota/02053/2008 | | 2/28/08 |  |  |
| 595 | CY082654 | A/Swine/Minnesota/02059/2008 | | 3/4/08 |  |  |
| 596 | CY082666 | A/Swine/Minnesota/02072/2008 | | 3/18/08 |  |  |
| 597 | CY082667 | A/Swine/Minnesota/02073/2008 | | 3/18/08 | Human |  |
| 598 | CY082670 | A/Swine/Minnesota/02081/2008 | | 3/19/08 |  |  |
| 599 | CY082677 | A/Swine/Minnesota/02092/2008 | | 3/6/08 |  |  |
| 600 | CY082678 | A/Swine/Minnesota/02093/2008 | | 3/7/08 |  |  |
| 601 | CY082679 | A/Swine/Minnesota/02094/2008 | | 3/13/08 |  |  |
| 602 | CY082683 | A/Swine/Minnesota/02098/2008 | | 3/31/08 | Human |  |
| 603 | CY082685 | A/Swine/Minnesota/02100/2008 | | 4/1/08 |  |  |
| 604 | CY082686 | A/Swine/Minnesota/02101/2008 | | 4/2/08 |  |  |
| 605 | CY082687 | A/Swine/Minnesota/02102/2008 | | 2/21/08 |  |  |
| 606 | CY082688 | A/Swine/Minnesota/02105/2008 | | 3/27/08 |  |  |
| 607 | CY082691 | A/Swine/Minnesota/02108/2008 | | 4/2/08 |  |  |
| 608 | CY082694 | A/Swine/Minnesota/02113/2008 | | 2/7/08 |  |  |
| 609 | CY082695 | A/Swine/Minnesota/02114/2008 | | 3/7/08 |  |  |
| 610 | CY082701 | A/Swine/Minnesota/02121/2008 | | 4/11/08 |  |  |
| 611 | CY082705 | A/Swine/Minnesota/02127/2008 | | 4/3/08 |  |  |
| 612 | CY082706 | A/Swine/Minnesota/02128/2008 | | 4/8/08 |  |  |
| 613 | CY082707 | A/Swine/Minnesota/02129/2008 | | 4/10/08 |  |  |
| 614 | CY082708 | A/Swine/Minnesota/02130/2008 | | 4/10/08 | Human |  |
| 615 | CY082711 | A/Swine/Minnesota/02134/2008 | | 4/18/08 | Human |  |
| 616 | CY082717 | A/Swine/Minnesota/02146/2008 | | 4/28/08 | Human |  |
| 617 | CY082718 | A/Swine/Minnesota/02148/2008 | | 5/1/08 | Human |  |
| 618 | CY082720 | A/Swine/Minnesota/02154/2008 | | 5/2/08 |  |  |
| 619 | CY082721 | A/Swine/Minnesota/02155/2008 | | 5/6/08 |  |  |
| 620 | CY082730 | A/Swine/Minnesota/02171/2008 | | 5/2/08 |  |  |
| 621 | CY082731 | A/Swine/Minnesota/02172/2008 | | 5/14/08 |  |  |
| 622 | CY082734 | A/Swine/Minnesota/02179/2008 | | 5/22/08 | Human |  |
| 623 | CY082735 | A/Swine/Minnesota/02180/2008 | | 5/9/08 | Human |  |
| 624 | CY082740 | A/Swine/Minnesota/02189/2008 | | 5/30/08 | Human |  |
| 625 | CY082743 | A/Swine/Minnesota/02194/2008 | | 5/28/08 |  |  |
| 626 | CY082745 | A/Swine/Minnesota/02197/2008 | | 6/10/08 |  |  |
| 627 | CY082746 | A/Swine/Minnesota/02200/2008 | | 5/7/08 |  |  |
| 628 | CY082747 | A/Swine/Minnesota/02201/2008 | | 5/22/08 |  |  |
| 629 | CY082748 | A/Swine/Minnesota/02202/2008 | | 5/29/08 |  |  |
| 630 | CY082750 | A/Swine/Minnesota/02209/2008 | | 6/19/08 |  |  |
| 631 | CY082752 | A/Swine/Minnesota/02215/2008 | | 6/12/08 |  |  |
| 632 | CY082759 | A/Swine/Minnesota/02223/2008 | | 7/3/08 | Human |  |
| 633 | CY082761 | A/Swine/Minnesota/02227/2008 | | 6/24/08 |  |  |
| 634 | CY082765 | A/Swine/Minnesota/02232/2008 | | 7/2/08 |  |  |
| 635 | CY082766 | A/Swine/Minnesota/02234/2008 | | 7/3/08 | Human |  |
| 636 | CY082767 | A/Swine/Minnesota/02235/2008 | | 7/3/08 | Human |  |
| 637 | CY082768 | A/Swine/Minnesota/02236/2008 | | 7/3/08 | Human |  |
| 638 | CY082769 | A/Swine/Minnesota/02237/2008 | | 7/11/08 |  |  |
| 639 | CY082770 | A/Swine/Minnesota/02239/2008 | | 3/7/08 | Human |  |
| 640 | CY082771 | A/Swine/Minnesota/02243/2008 | | 7/3/08 | Human |  |
| 641 | CY082773 | A/Swine/Minnesota/02250/2008 | | 7/30/08 | Human |  |
| 642 | CY082774 | A/Swine/Minnesota/02252/2008 | | 8/4/08 |  |  |
| 643 | CY082775 | A/Swine/Minnesota/02253/2008 | | 8/4/08 | Human |  |
| 644 | CY082781 | A/Swine/Minnesota/02266/2008 | | 8/15/08 | Human |  |
| 645 | CY082782 | A/Swine/Minnesota/02267/2008 | | 8/6/08 | Human |  |
| 646 | CY082787 | A/Swine/Minnesota/02272/2008 | |  |  |  |
| 647 | CY082789 | A/Swine/Minnesota/02277/2008 | | 9/4/08 |  |  |
| 648 | CY082790 | A/Swine/Minnesota/02278/2008 | | 8/12/08 |  |  |
| 649 | CY082791 | A/Swine/Minnesota/02279/2008 | | 8/25/08 |  |  |
| 650 | CY082795 | A/Swine/Minnesota/02285/2008 | | 9/2/08 |  |  |
| 651 | CY082797 | A/Swine/Minnesota/02290/2008 | | 9/8/08 |  |  |
| 652 | CY082800 | A/Swine/Minnesota/02293/2008 | | 9/10/08 | Human |  |
| 653 | CY082801 | A/Swine/Minnesota/02294/2008 | | 9/10/08 | Human |  |
| 654 | CY082802 | A/Swine/Minnesota/02295/2008 | | 9/11/08 | Human |  |
| 655 | CY082805 | A/Swine/Minnesota/02298/2008 | | 9/10/08 |  |  |
| 656 | CY082807 | A/Swine/Minnesota/02300/2008 | | 9/17/08 | Human |  |
| 657 | CY082811 | A/Swine/Minnesota/02309/2008 | | 9/12/08 |  |  |
| 658 | CY082812 | A/Swine/Minnesota/02310/2008 | | 9/12/08 |  |  |
| 659 | CY082815 | A/Swine/Minnesota/02316/2008 | | 9/25/08 |  |  |
| 660 | CY082817 | A/Swine/Minnesota/02318/2008 | | 9/25/08 | Human |  |
| 661 | CY082818 | A/Swine/Minnesota/02319/2008 | | 9/25/08 | Human |  |
| 662 | CY082820 | A/Swine/Minnesota/02321/2008 | | 9/4/08 | Human |  |
| 663 | CY082821 | A/Swine/Minnesota/02324/2008 | | 9/10/08 |  |  |
| 664 | CY082826 | A/Swine/Minnesota/02332/2008 | | 10/2/08 | Human |  |
| 665 | CY082830 | A/Swine/Minnesota/02340/2008 | | 10/6/08 | Human |  |
| 666 | CY082833 | A/Swine/Minnesota/02345/2008 | | 9/30/08 |  |  |
| 667 | CY082836 | A/Swine/Minnesota/02348/2008 | | 10/3/08 | Human |  |
| 668 | CY082837 | A/Swine/Minnesota/02350/2008 | | 9/16/08 |  |  |
| 669 | CY082845 | A/Swine/Minnesota/02358/2008 | | 10/9/08 |  |  |
| 670 | CY082847 | A/Swine/Minnesota/02364/2008 | | 10/14/08 |  |  |
| 671 | CY082848 | A/Swine/Minnesota/02366/2008 | | 10/23/08 |  |  |
| 672 | CY082849 | A/Swine/Minnesota/02367/2008 | | 10/23/08 | Human |  |
| 673 | CY082850 | A/Swine/Minnesota/02368/2008 | | 10/23/08 | Human |  |
| 674 | CY082851 | A/Swine/Minnesota/02369/2008 | | 10/23/08 |  |  |
| 675 | CY082853 | A/Swine/Minnesota/02371/2008 | | 9/5/08 |  |  |
| 676 | CY082865 | A/Swine/Minnesota/02387/2008 | | 10/22/08 |  |  |
| 677 | CY082867 | A/Swine/Minnesota/02399/2008 | | 10/28/08 |  |  |
| 678 | CY082868 | A/Swine/Minnesota/02400/2008 | | 10/28/08 |  |  |
| 679 | CY082869 | A/Swine/Minnesota/02401/2008 | | 10/30/08 | Human |  |
| 680 | CY082870 | A/Swine/Minnesota/02402/2008 | | 10/30/08 |  |  |
| 681 | CY082873 | A/Swine/Minnesota/02406/2008 | | 10/22/08 |  |  |
| 682 | CY082874 | A/Swine/Minnesota/02407/2008 | | 10/24/08 |  |  |
| 683 | CY082877 | A/Swine/Minnesota/02412/2008 | | 10/29/08 | Human |  |
| 684 | CY082881 | A/Swine/Minnesota/02416/2008 | | 10/17/08 | Human |  |
| 685 | CY082882 | A/Swine/Minnesota/02417/2008 | | 10/22/08 |  |  |
| 686 | CY082883 | A/Swine/Minnesota/02418/2008 | | 10/23/08 |  |  |
| 687 | CY082885 | A/Swine/Minnesota/02420/2008 | | 10/24/08 |  |  |
| 688 | CY082889 | A/Swine/Minnesota/02425/2008 | | 11/4/08 |  |  |
| 689 | CY082890 | A/Swine/Minnesota/02426/2008 | | 11/5/08 | Human |  |
| 690 | CY082892 | A/Swine/Minnesota/02430/2008 | | 10/30/08 |  |  |
| 691 | CY082895 | A/Swine/Minnesota/02433/2008 | | 11/11/08 |  |  |
| 692 | CY082896 | A/Swine/Minnesota/02434/2008 | | 11/11/08 |  |  |
| 693 | CY082898 | A/Swine/Minnesota/02438/2008 | | 11/12/08 |  |  |
| 694 | CY082904 | A/Swine/Minnesota/02447/2008 | | 11/14/08 | Human |  |
| 695 | CY082905 | A/Swine/Minnesota/02448/2008 | | 11/14/08 |  |  |
| 696 | CY082910 | A/Swine/Minnesota/02453/2008 | | 11/21/08 |  |  |
| 697 | CY082922 | A/Swine/Minnesota/02469/2008 | | 11/19/08 |  |  |
| 698 | CY082923 | A/Swine/Minnesota/02470/2008 | | 11/24/08 | Human |  |
| 699 | CY082924 | A/Swine/Minnesota/02471/2008 | | 11/24/08 | Human |  |
| 700 | CY082925 | A/Swine/Minnesota/02472/2008 | | 11/26/08 |  |  |
| 701 | CY082926 | A/Swine/Minnesota/02473/2008 | | 11/28/08 |  |  |
| 702 | CY082927 | A/Swine/Minnesota/02474/2008 | | 12/8/08 | Human |  |
| 703 | CY082928 | A/Swine/Minnesota/02475/2008 | | 12/8/08 | Human |  |
| 704 | CY082932 | A/Swine/Minnesota/02483/2008 | | 12/8/08 | Human |  |
| 705 | CY082939 | A/Swine/Minnesota/02492/2008 | | 12/5/08 | Human |  |
| 706 | CY082942 | A/Swine/Minnesota/02500/2008 | | 12/8/08 |  |  |
| 707 | CY082943 | A/Swine/Minnesota/02501/2008 | | 12/24/08 |  |  |
| 708 | CY082944 | A/Swine/Minnesota/02502/2008 | | 12/24/08 |  |  |
| 709 | CY082946 | A/Swine/Minnesota/02509/2008 | | 12/23/08 | Human |  |
| 710 | CY082953 | A/Swine/Minnesota/02524/2008 | | 11/25/08 |  |  |
| 711 | CY082954 | A/Swine/Minnesota/02525/2008 | | 12/3/08 |  |  |
| 712 | ADD21430 | A/Swine/Minnesota/02949/2009 | | 9/30/09 |  |  |
| 713 | ADD21431 | A/Swine/Minnesota/02950/2009 | | 10/16/09 |  |  |
| 714 | ADD21432 | A/Swine/Minnesota/02951/2009 | | 10/16/09 |  |  |
| 715 | ADC79608 | A/Swine/Minnesota/02953/2010 | | 1/6/10 | Human |  |
| 716 | ADC79609 | A/Swine/Minnesota/02954/2010 | | 1/6/10 | Human |  |
| 717 | ADD22553 | A/Swine/Minnesota/02976/2010 | | 1/12/10 |  |  |
| 718 | ADD22552 | A/Swine/Minnesota/02979/2010 | | 2/17/10 |  |  |
| 719 | ADD97109 | A/Swine/Minnesota/02982/2010 | | 1/19/10 |  |  |
| 720 | ADG85794 | A/Swine/Minnesota/03000/2010 | | 4/7/10 | Human |  |
| 721 | ADJ18758 | A/Swine/Minnesota/03018/2010 | | 5/17/10 | Human |  |
| 722 | ADJ18757 | A/Swine/Minnesota/03022/2010 | | 5/17/10 |  |  |
| 723 | ADJ18751 | A/Swine/Minnesota/03023/2010 | | 4/26/10 | Human |  |
| 724 | ADJ18752 | A/Swine/Minnesota/03024/2010 | | 4/26/10 | Human |  |
| 725 | ADJ18753 | A/Swine/Minnesota/03025/2010 | | 4/27/10 |  |  |
| 726 | ADJ18759 | A/Swine/Minnesota/03027/2010 | | 3/18/10 | Human |  |
| 727 | ADM13521 | A/Swine/Minnesota/03043/2010 | | 7/7/10 | Human |  |
| 728 | ACL79904 | A/Swine/Minnesota/07002083/2007/H1N1 | |  | Human | ACL79902 |
| 729 | CY082960 | A/Swine/Minnesota/16980-6/2008 | |  |  |  |
| 730 | CY082961 | A/Swine/Minnesota/16980-7/2008 | |  |  |  |
| 731 | [CY040473](http://www.ncbi.nlm.nih.gov/entrez/viewer.fcgi??db=nucleotide&val=CY040473) | A/Swine/Minnesota/225-10/2008 | | 11/12/08 |  |  |
| 732 | [CY040474](http://www.ncbi.nlm.nih.gov/entrez/viewer.fcgi??db=nucleotide&val=CY040474) | A/Swine/Minnesota/225-11/2008 | | 11/13/08 | Human |  |
| 733 | [CY040475](http://www.ncbi.nlm.nih.gov/entrez/viewer.fcgi??db=nucleotide&val=CY040475) | A/Swine/Minnesota/225-3/2008 | | 11/7/08 |  |  |
| 734 | [CY040476](http://www.ncbi.nlm.nih.gov/entrez/viewer.fcgi??db=nucleotide&val=CY040476) | A/Swine/Minnesota/225-4/2008 | | 11/10/08 |  |  |
| 735 | [CY040477](http://www.ncbi.nlm.nih.gov/entrez/viewer.fcgi??db=nucleotide&val=CY040477) | A/Swine/Minnesota/225-5/2008 | | 11/11/08 | Human |  |
| 736 | [CY040479](http://www.fludb.org/brc/fluSegmentDetails.do?ncbiGenomicAccession=CY040479&context=1295037256998) | A/Swine/Minnesota/63607-12/2008 | | 10/8/08 |  |  |
| 737 | [CY040480](http://www.ncbi.nlm.nih.gov/entrez/viewer.fcgi??db=nucleotide&val=CY040480) | A/Swine/Minnesota/63607-15/2008 | | 10/9/08 |  |  |
| 738 | [CY040481](http://www.ncbi.nlm.nih.gov/entrez/viewer.fcgi??db=nucleotide&val=CY040481) | A/Swine/Minnesota/63607-17/2008 | | 10/10/08 |  |  |
| 739 | [CY040482](http://www.ncbi.nlm.nih.gov/entrez/viewer.fcgi??db=nucleotide&val=CY040482) | A/Swine/Minnesota/63607-21/2008 | | 10/15/08 |  |  |
| 740 | [CY040483](http://www.ncbi.nlm.nih.gov/entrez/viewer.fcgi??db=nucleotide&val=CY040483) | A/Swine/Minnesota/63607-26/2008 | | 10/22/08 |  |  |
| 741 | [CY040485](http://www.ncbi.nlm.nih.gov/entrez/viewer.fcgi??db=nucleotide&val=CY040485) | A/Swine/Minnesota/63607-30/2008 | | 10/24/08 |  |  |
| 742 | [CY040486](http://www.ncbi.nlm.nih.gov/entrez/viewer.fcgi??db=nucleotide&val=CY040486) | A/Swine/Minnesota/63607-31/2008 | | 10/28/08 |  |  |
| 743 | [CY040488](http://www.ncbi.nlm.nih.gov/entrez/viewer.fcgi??db=nucleotide&val=CY040488) | A/Swine/Minnesota/63607-35/2008 | | 10/29/08 |  |  |
| 744 | [CY040489](http://www.ncbi.nlm.nih.gov/entrez/viewer.fcgi??db=nucleotide&val=CY040489) | A/Swine/Minnesota/63607-4/2008 | | 10/3/08 |  |  |
| 745 | [CY040490](http://www.ncbi.nlm.nih.gov/entrez/viewer.fcgi??db=nucleotide&val=CY040490) | A/Swine/Minnesota/63607-40/2008 | | 10/31/08 |  |  |
| 746 | [CY040492](http://www.ncbi.nlm.nih.gov/entrez/viewer.fcgi??db=nucleotide&val=CY040492) | A/Swine/Minnesota/63607-7/2008 | | 10/7/08 |  |  |
| 747 | ACS92958 | A/Swine/Minnesota/SG-00239/2007 | |  | Human | ACI89695 |
| 748 | CY081602 | A/Swine/Missouri/00064/2003 | | 4/2/03 |  |  |
| 749 | CY081604 | A/Swine/Missouri/00101/2003 | | 4/4/03 |  |  |
| 750 | CY081605 | A/Swine/Missouri/00102/2003 | | 4/4/03 |  |  |
| 751 | CY081606 | A/Swine/Missouri/00103/2003 | | 4/4/03 | Human |  |
| 752 | CY081618 | A/Swine/Missouri/00123/2003 | | 7/25/03 |  |  |
| 753 | CY081621 | A/Swine/Missouri/00127/2003 | | 7/29/03 |  |  |
| 754 | CY081625 | A/Swine/Missouri/00131/2003 | | 8/12/03 |  |  |
| 755 | CY081626 | A/Swine/Missouri/00132/2003 | | 8/12/03 |  |  |
| 756 | CY081664 | A/Swine/Missouri/00224/2003 | |  |  |  |
| 757 | CY081678 | A/Swine/Missouri/00243/2004 | | 10/14/03 |  |  |
| 758 | CY081690 | A/Swine/Missouri/00263/2004 | | 3/5/04 |  |  |
| 759 | CY081695 | A/Swine/Missouri/00268/2004 | | 3/15/04 |  |  |
| 760 | CY081708 | A/Swine/Missouri/00298/2004 | | 5/6/04 |  |  |
| 761 | CY081733 | A/Swine/Missouri/00348/2004 | | 7/21/04 |  |  |
| 762 | CY081742 | A/Swine/Missouri/00366/2004 | | 9/27/04 |  |  |
| 763 | CY081747 | A/Swine/Missouri/00373/2004 | | 9/27/04 |  |  |
| 764 | CY081752 | A/Swine/Missouri/00379/2004 | | 8/6/04 |  |  |
| 765 | CY081769 | A/Swine/Missouri/00414/2004 | | 9/24/04 |  |  |
| 766 | CY081790 | A/Swine/Missouri/00460/2004 | | 12/29/04 |  |  |
| 767 | CY081804 | A/Swine/Missouri/00494/2005 | | 2/8/05 |  |  |
| 768 | CY081805 | A/Swine/Missouri/00498/2005 | | 2/10/05 |  |  |
| 769 | CY081815 | A/Swine/Missouri/00517/2005 | | 2/24/05 |  |  |
| 770 | CY081840 | A/Swine/Missouri/00564/2005 | | 3/30/05 |  |  |
| 771 | CY081866 | A/Swine/Missouri/00615/2005 | | 5/4/05 |  |  |
| 772 | CY081867 | A/Swine/Missouri/00617/2005 | | 5/11/05 |  |  |
| 773 | CY081868 | A/Swine/Missouri/00618/2005 | | 5/19/05 |  |  |
| 774 | CY081878 | A/Swine/Missouri/00648/2005 | | 6/9/05 |  |  |
| 775 | CY081879 | A/Swine/Missouri/00649/2005 | | 6/10/05 |  |  |
| 776 | CY081884 | A/Swine/Missouri/00663/2005 | | 7/6/05 |  |  |
| 777 | CY081897 | A/Swine/Missouri/00680/2005 | | 7/28/05 |  |  |
| 778 | CY081911 | A/Swine/Missouri/00707/2005 | | 8/11/05 |  |  |
| 779 | CY081924 | A/Swine/Missouri/00727/2005 | | 9/14/05 |  |  |
| 780 | CY081943 | A/Swine/Missouri/00762/2005 | | 10/7/05 |  |  |
| 781 | CY081949 | A/Swine/Missouri/00776/2005 | | 7/15/05 |  |  |
| 782 | CY081950 | A/Swine/Missouri/00777/2005 | | 7/15/05 |  |  |
| 783 | CY081962 | A/Swine/Missouri/00795/2005 | | 9/27/05 |  |  |
| 784 | CY081977 | A/Swine/Missouri/00824/2005 | | 10/18/05 |  |  |
| 785 | CY081979 | A/Swine/Missouri/00826/2005 | | 10/19/05 |  |  |
| 786 | CY081986 | A/Swine/Missouri/00836/2005 | | 10/26/05 |  |  |
| 787 | CY081992 | A/Swine/Missouri/00848/2005 | | 11/8/05 |  |  |
| 788 | CY082000 | A/Swine/Missouri/00861/2005 | | 11/15/05 |  |  |
| 789 | CY082042 | A/Swine/Missouri/00941/2006 | | 9/20/06 |  |  |
| 790 | CY082044 | A/Swine/Missouri/00945/2006 | | 9/21/06 |  |  |
| 791 | CY082050 | A/Swine/Missouri/00957/2006 | | 1/11/06 |  |  |
| 792 | CY082059 | A/Swine/Missouri/00976/2006 | | 2/1/06 |  |  |
| 793 | CY082064 | A/Swine/Missouri/00983/2006 | | 2/3/06 |  |  |
| 794 | CY082065 | A/Swine/Missouri/00984/2006 | | 2/3/06 |  |  |
| 795 | CY082069 | A/Swine/Missouri/00992/2006 | | 2/8/06 |  |  |
| 796 | CY082128 | A/Swine/Missouri/01077/2006 | | 3/31/06 |  |  |
| 797 | CY082135 | A/Swine/Missouri/01090/2006 | | 4/11/06 |  |  |
| 798 | CY082145 | A/Swine/Missouri/01103/2006 | | 4/20/06 |  |  |
| 799 | CY082154 | A/Swine/Missouri/01119/2006 | | 5/2/06 | Human |  |
| 800 | CY082158 | A/Swine/Missouri/01125/2006 | | 5/4/06 |  |  |
| 801 | CY082178 | A/Swine/Missouri/01167/2006 | | 6/21/06 |  |  |
| 802 | CY082179 | A/Swine/Missouri/01168/2006 | | 6/22/06 |  |  |
| 803 | CY082187 | A/Swine/Missouri/01180/2006 | | 6/29/06 |  |  |
| 804 | CY082192 | A/Swine/Missouri/01187/2006 | | 7/14/06 |  |  |
| 805 | CY082194 | A/Swine/Missouri/01190/2006 | | 7/28/06 |  |  |
| 806 | CY082201 | A/Swine/Missouri/01204/2006 | | 8/17/06 |  |  |
| 807 | CY082206 | A/Swine/Missouri/01212/2006 | | 10/5/06 |  |  |
| 808 | CY082227 | A/Swine/Missouri/01246/2006 | | 10/20/06 |  |  |
| 809 | CY082244 | A/Swine/Missouri/01265/2006 | | 10/11/06 |  |  |
| 810 | CY082314 | A/Swine/Missouri/01373/2006 | | 11/30/06 |  |  |
| 811 | CY082360 | A/Swine/Missouri/01461/2007 | | 2/14/07 |  |  |
| 812 | CY082366 | A/Swine/Missouri/01473/2007 | | 2/22/07 |  |  |
| 813 | CY082381 | A/Swine/Missouri/01531/2007 | | 3/23/07 |  |  |
| 814 | CY082388 | A/Swine/Missouri/01553/2007 | | 4/12/07 |  |  |
| 815 | CY082424 | A/Swine/Missouri/01656/2007 | | 6/20/07 |  |  |
| 816 | CY082428 | A/Swine/Missouri/01660/2007 | | 6/28/07 |  |  |
| 817 | CY082467 | A/Swine/Missouri/01750/2007 | | 8/30/07 |  |  |
| 818 | CY082489 | A/Swine/Missouri/01819/2007 | | 10/26/07 |  |  |
| 819 | CY082536 | A/Swine/Missouri/01881/2007 | | 11/30/07 |  |  |
| 820 | CY082576 | A/Swine/Missouri/01945/2007 | | 12/14/07 |  |  |
| 821 | CY082584 | A/Swine/Missouri/01956/2007 | | 12/19/07 |  |  |
| 822 | CY082605 | A/Swine/Missouri/01990/2008 | | 1/3/08 |  |  |
| 823 | CY082614 | A/Swine/Missouri/02004/2008 | | 2/4/08 |  |  |
| 824 | CY082621 | A/Swine/Missouri/02012/2008 | | 1/31/08 |  |  |
| 825 | CY082630 | A/Swine/Missouri/02025/2008 | | 2/13/08 |  |  |
| 826 | CY082636 | A/Swine/Missouri/02036/2008 | | 2/7/08 |  |  |
| 827 | CY082643 | A/Swine/Missouri/02043/2008 | | 2/21/08 |  |  |
| 828 | CY082655 | A/Swine/Missouri/02060/2008 | | 3/5/08 |  |  |
| 829 | CY082684 | A/Swine/Missouri/02099/2008 | | 4/1/08 |  |  |
| 830 | CY082693 | A/Swine/Missouri/02111/2008 | | 4/9/08 |  |  |
| 831 | CY082722 | A/Swine/Missouri/02157/2008 | | 5/8/08 |  |  |
| 832 | CY082760 | A/Swine/Missouri/02224/2008 | | 6/10/08 |  |  |
| 833 | CY082764 | A/Swine/Missouri/02230/2008 | | 7/8/08 |  |  |
| 834 | CY082779 | A/Swine/Missouri/02263/2008 | | 8/14/08 |  |  |
| 835 | CY082788 | A/Swine/Missouri/02273/2008 | | 8/26/08 |  |  |
| 836 | CY082792 | A/Swine/Missouri/02280/2008 | | 8/26/08 | Human |  |
| 837 | CY082796 | A/Swine/Missouri/02289/2008 | | 9/5/08 | Human |  |
| 838 | CY082804 | A/Swine/Missouri/02297/2008 | | 8/29/08 |  |  |
| 839 | CY082806 | A/Swine/Missouri/02299/2008 | | 9/12/08 |  |  |
| 840 | CY082813 | A/Swine/Missouri/02312/2008 | | 9/18/08 |  |  |
| 841 | CY082844 | A/Swine/Missouri/02357/2008 | | 10/9/08 |  |  |
| 842 | CY082857 | A/Swine/Missouri/02379/2008 | | 10/14/08 |  |  |
| 843 | CY082859 | A/Swine/Missouri/02381/2008 | | 10/15/08 |  |  |
| 844 | CY082884 | A/Swine/Missouri/02419/2008 | | 10/23/08 |  |  |
| 845 | CY082903 | A/Swine/Missouri/02446/2008 | | 11/12/08 |  |  |
| 846 | CY082906 | A/Swine/Missouri/02449/2008 | | 11/19/08 |  |  |
| 847 | CY082915 | A/Swine/Missouri/02458/2008 | | 11/20/08 |  |  |
| 848 | CY082940 | A/Swine/Missouri/02495/2008 | | 12/17/08 |  |  |
| 849 | CY082941 | A/Swine/Missouri/02496/2008 | | 12/18/08 |  |  |
| 850 | CY082945 | A/Swine/Missouri/02507/2008 | | 12/16/08 | Human |  |
| 851 | ADK26591 | A/Swine/Missouri/03035/2010 | | 6/18/10 | Human |  |
| 852 | [CY040493](http://www.ncbi.nlm.nih.gov/entrez/viewer.fcgi??db=nucleotide&val=CY040493) | A/Swine/Missouri/63607-16/2008 | | 10/10/08 |  |  |
| 853 | ADF83511 | A/Swine/MN/23506/2009 | | 5/7/09 |  |  |
| 854 | ADD64944 | A/Swine/MN/8761/2010 | | 2/16/10 |  |  |
| 855 | ADD64948 | A/Swine/MN/8762-1/2010 | | 2/16/10 |  |  |
| 856 | ADD64952 | A/Swine/MN/8762-2/2010 | | 2/16/10 |  |  |
| 857 | ADG85229 | A/Swine/MO/15534/2010 | | 3/24/10 |  |  |
| 858 | ADG85237 | A/Swine/MO/17314/2010 | | 3/31/10 |  |  |
| 859 | ACM17265 | A/Swine/NC/00573/2005/H1N1 | |  | Human | ACM17261 |
| 860 | ADG85225 | A/Swine/NC/13598/2010 | | 3/9/10 |  |  |
| 861 | ADG85249 | A/Swine/NC/19646/2010 | | 4/20/10 |  |  |
| 862 | ADB82962 | A/Swine/NC/34543/2009 | | 11/24/09 |  |  |
| 863 | ADB82966 | A/Swine/NC/34752/2009 | | 12/14/09 |  |  |
| 864 | CY081640 | A/Swine/Nebraska/00176/2003 | | 8/15/03 |  |  |
| 865 | CY081642 | A/Swine/Nebraska/00178/2003 | | 8/29/03 |  |  |
| 866 | CY081643 | A/Swine/Nebraska/00184/2003 | | 9/16/03 |  |  |
| 867 | CY081651 | A/Swine/Nebraska/00200/2003 | | 10/23/03 |  |  |
| 868 | CY081665 | A/Swine/Nebraska/00225/2003 | | 12/3/03 |  |  |
| 869 | CY081728 | A/Swine/Nebraska/00343/2004 | | 7/5/04 |  |  |
| 870 | CY081729 | A/Swine/Nebraska/00344/2004 | | 7/5/04 |  |  |
| 871 | CY081730 | A/Swine/Nebraska/00345/2004 | | 7/5/04 |  |  |
| 872 | CY081731 | A/Swine/Nebraska/00346/2004 | | 7/5/04 |  |  |
| 873 | CY081737 | A/Swine/Nebraska/00356/2004 | | 7/30/04 |  |  |
| 874 | CY081738 | A/Swine/Nebraska/00357/2004 | | 7/30/04 |  |  |
| 875 | CY081739 | A/Swine/Nebraska/00358/2004 | | 7/30/04 |  |  |
| 876 | CY081755 | A/Swine/Nebraska/00390/2004 | | 8/18/04 |  |  |
| 877 | CY081768 | A/Swine/Nebraska/00413/2004 | | 9/22/04 |  |  |
| 878 | CY081794 | A/Swine/Nebraska/00467/2005 | | 1/5/05 |  |  |
| 879 | CY081847 | A/Swine/Nebraska/00581/2005 | | 4/29/05 |  |  |
| 880 | CY081856 | A/Swine/Nebraska/00594/2005 | | 4/29/05 |  |  |
| 881 | CY081999 | A/Swine/Nebraska/00860/2005 | | 11/15/05 |  |  |
| 882 | CY082157 | A/Swine/Nebraska/01124/2006 | | 5/4/06 |  |  |
| 883 | CY082176 | A/Swine/Nebraska/01164/2006 | | 6/15/06 |  |  |
| 884 | CY082188 | A/Swine/Nebraska/01182/2006 | | 6/30/06 |  |  |
| 885 | CY082243 | A/Swine/Nebraska/01264/2006 | | 10/9/06 |  |  |
| 886 | CY082353 | A/Swine/Nebraska/01448/2007 | | 1/18/07 |  |  |
| 887 | CY082410 | A/Swine/Nebraska/01616/2007 | | 5/10/07 |  |  |
| 888 | CY082423 | A/Swine/Nebraska/01655/2007 | | 6/18/07 |  |  |
| 889 | CY082475 | A/Swine/Nebraska/01775/2007 | | 9/28/07 |  |  |
| 890 | CY082546 | A/Swine/Nebraska/01894/2007 | | 11/29/07 |  |  |
| 891 | CY082562 | A/Swine/Nebraska/01920/2007 | | 12/12/07 | Human |  |
| 892 | CY082622 | A/Swine/Nebraska/02013/2008 | | 2/4/08 |  |  |
| 893 | CY082637 | A/Swine/Nebraska/02037/2008 | | 2/12/08 | Human |  |
| 894 | CY082757 | A/Swine/Nebraska/02221/2008 | | 6/26/08 | Human |  |
| 895 | CY082758 | A/Swine/Nebraska/02222/2008 | | 6/26/08 | Human |  |
| 896 | CY082785 | A/Swine/Nebraska/02270/2008 | | 8/13/08 |  |  |
| 897 | CY082793 | A/Swine/Nebraska/02281/2008 | | 8/26/08 |  |  |
| 898 | CY082831 | A/Swine/Nebraska/02341/2008 | | 5/14/08 |  |  |
| 899 | CY082963 | A/Swine/Nebraska/02436/2008 | | 11/12/08 | Human |  |
| 900 | CY082913 | A/Swine/Nebraska/02456/2008 | | 3/20/08 |  |  |
| 901 | CY082914 | A/Swine/Nebraska/02457/2008 | | 11/5/08 |  |  |
| 902 | CY081597 | A/Swine/North Carolina/00056/2003 | | 2/13/03 | Human |  |
| 903 | CY081600 | A/Swine/North Carolina/00060/2003 | | 2/26/03 |  |  |
| 904 | CY081601 | A/Swine/North Carolina/00062/2003 | | 3/7/03 |  |  |
| 905 | CY081603 | A/Swine/North Carolina/00100/2003 | | 4/3/03 |  |  |
| 906 | CY081610 | A/Swine/North Carolina/00111/2003 | | 5/23/03 |  |  |
| 907 | CY081611 | A/Swine/North Carolina/00112/2003 | | 6/20/03 |  |  |
| 908 | CY081612 | A/Swine/North Carolina/00113/2003 | | 6/20/03 |  |  |
| 909 | CY081613 | A/Swine/North Carolina/00114/2003 | | 6/20/03 |  |  |
| 910 | CY081615 | A/Swine/North Carolina/00116/2003 | | 7/2/03 |  |  |
| 911 | CY081616 | A/Swine/North Carolina/00121/2003 | | 7/18/03 |  |  |
| 912 | CY081620 | A/Swine/North Carolina/00125/2003 | | 7/29/03 |  |  |
| 913 | CY081628 | A/Swine/North Carolina/00138/2003 | | 8/21/03 |  |  |
| 914 | CY081629 | A/Swine/North Carolina/00143/2003 | | 8/29/03 |  |  |
| 915 | CY081630 | A/Swine/North Carolina/00144/2003 | | 9/4/03 |  |  |
| 916 | CY081631 | A/Swine/North Carolina/00146/2003 | | 9/11/03 |  |  |
| 917 | CY081632 | A/Swine/North Carolina/00151/2003 | | 9/12/03 |  |  |
| 918 | CY081633 | A/Swine/North Carolina/00152/2003 | | 9/12/03 |  |  |
| 919 | CY081635 | A/Swine/North Carolina/00157/2003 | | 10/8/03 |  |  |
| 920 | CY081639 | A/Swine/North Carolina/00175/2003 | | 7/25/03 |  |  |
| 921 | CY081644 | A/Swine/North Carolina/00187/2003 | | 10/3/03 |  |  |
| 922 | CY081654 | A/Swine/North Carolina/00209/2003 | | 11/13/03 |  |  |
| 923 | CY081661 | A/Swine/North Carolina/00219/2003 | | 11/28/03 |  |  |
| 924 | CY081662 | A/Swine/North Carolina/00220/2003 | | 11/28/03 |  |  |
| 925 | CY081663 | A/Swine/North Carolina/00221/2003 | | 11/28/03 |  |  |
| 926 | CY081666 | A/Swine/North Carolina/00227/2003 | | 12/4/03 |  |  |
| 927 | CY081667 | A/Swine/North Carolina/00228/2003 | | 12/10/03 |  |  |
| 928 | CY081672 | A/Swine/North Carolina/00236/2003 | | 12/31/03 |  |  |
| 929 | CY081673 | A/Swine/North Carolina/00237/2004 | | 1/6/04 |  |  |
| 930 | CY081674 | A/Swine/North Carolina/00238/2004 | | 1/6/04 |  |  |
| 931 | CY081686 | A/Swine/North Carolina/00253/2004 | | 2/25/04 |  |  |
| 932 | CY081689 | A/Swine/North Carolina/00261/2004 | | 3/4/04 |  |  |
| 933 | CY081692 | A/Swine/North Carolina/00265/2004 | | 3/16/04 |  |  |
| 934 | CY081694 | A/Swine/North Carolina/00267/2004 | | 3/18/04 |  |  |
| 935 | CY081696 | A/Swine/North Carolina/00270/2004 | | 3/23/04 |  |  |
| 936 | CY081698 | A/Swine/North Carolina/00273/2004 | | 3/31/04 |  |  |
| 937 | CY081699 | A/Swine/North Carolina/00275/2004 | | 3/31/04 |  |  |
| 938 | CY081701 | A/Swine/North Carolina/00282/2004 | | 4/9/04 |  |  |
| 939 | CY081702 | A/Swine/North Carolina/00283/2004 | | 4/15/04 |  |  |
| 940 | CY081704 | A/Swine/North Carolina/00286/2004 | | 4/21/04 |  |  |
| 941 | CY081705 | A/Swine/North Carolina/00290/2004 | | 4/23/04 |  |  |
| 942 | CY081712 | A/Swine/North Carolina/00307/2004 | | 5/21/04 |  |  |
| 943 | CY081715 | A/Swine/North Carolina/00318/2004 | | 6/24/04 |  |  |
| 944 | CY081716 | A/Swine/North Carolina/00319/2004 | | 6/24/04 |  |  |
| 945 | CY081717 | A/Swine/North Carolina/00320/2004 | | 6/24/04 |  |  |
| 946 | CY081718 | A/Swine/North Carolina/00321/2004 | | 6/24/04 |  |  |
| 947 | CY081721 | A/Swine/North Carolina/00329/2003 | | 10/29/03 |  |  |
| 948 | CY081722 | A/Swine/North Carolina/00330/2003 | | 11/28/03 |  |  |
| 949 | CY081723 | A/Swine/North Carolina/00333/2004 | | 2/25/04 |  |  |
| 950 | CY081726 | A/Swine/North Carolina/00338/2004 | | 6/22/04 |  |  |
| 951 | CY081727 | A/Swine/North Carolina/00340/2004 | | 6/30/04 |  |  |
| 952 | CY081732 | A/Swine/North Carolina/00347/2004 | | 7/8/04 |  |  |
| 953 | CY081734 | A/Swine/North Carolina/00349/2004 | | 7/22/04 |  |  |
| 954 | CY081735 | A/Swine/North Carolina/00351/2004 | | 7/23/04 |  |  |
| 955 | CY081740 | A/Swine/North Carolina/00361/2004 | | 8/3/04 |  |  |
| 956 | CY081741 | A/Swine/North Carolina/00362/2004 | | 10/7/04 |  |  |
| 957 | CY081746 | A/Swine/North Carolina/00371/2004 | | 10/7/04 |  |  |
| 958 | CY081748 | A/Swine/North Carolina/00374/2004 | | 7/13/04 |  |  |
| 959 | CY081749 | A/Swine/North Carolina/00375/2004 | | 7/14/04 |  |  |
| 960 | CY081750 | A/Swine/North Carolina/00377/2004 | | 7/30/04 |  |  |
| 961 | CY081751 | A/Swine/North Carolina/00378/2004 | | 8/3/04 |  |  |
| 962 | CY081753 | A/Swine/North Carolina/00382/2004 | | 8/11/04 |  |  |
| 963 | CY081757 | A/Swine/North Carolina/00394/2004 | | 8/25/04 |  |  |
| 964 | CY081758 | A/Swine/North Carolina/00396/2004 | | 8/27/04 |  |  |
| 965 | CY081759 | A/Swine/North Carolina/00397/2004 | | 9/2/04 |  |  |
| 966 | CY081761 | A/Swine/North Carolina/00402/2004 | | 9/9/04 |  |  |
| 967 | CY081767 | A/Swine/North Carolina/00411/2004 | | 9/15/04 |  |  |
| 968 | CY081773 | A/Swine/North Carolina/00428/2004 | | 11/3/04 |  |  |
| 969 | CY081775 | A/Swine/North Carolina/00431/2004 | | 11/11/04 |  |  |
| 970 | CY081779 | A/Swine/North Carolina/00437/2004 | | 12/9/04 |  |  |
| 971 | CY081780 | A/Swine/North Carolina/00438/2004 | | 12/9/04 |  |  |
| 972 | CY081781 | A/Swine/North Carolina/00439/2004 | | 12/9/04 |  |  |
| 973 | CY081785 | A/Swine/North Carolina/00451/2004 | | 12/3/04 |  |  |
| 974 | CY081787 | A/Swine/North Carolina/00453/2004 | | 12/9/04 |  |  |
| 975 | CY081788 | A/Swine/North Carolina/00455/2004 | | 12/15/04 |  |  |
| 976 | CY081791 | A/Swine/North Carolina/00463/2005 | | 1/4/05 |  |  |
| 977 | CY081797 | A/Swine/North Carolina/00478/2005 | | 1/13/05 |  |  |
| 978 | CY081799 | A/Swine/North Carolina/00482/2005 | | 1/18/05 |  |  |
| 979 | CY081800 | A/Swine/North Carolina/00485/2005 | | 1/21/05 |  |  |
| 980 | CY081802 | A/Swine/North Carolina/00489/2005 | | 1/27/05 |  |  |
| 981 | CY081810 | A/Swine/North Carolina/00503/2005 | | 2/16/05 |  |  |
| 982 | CY081811 | A/Swine/North Carolina/00504/2005 | | 2/16/05 |  |  |
| 983 | CY081819 | A/Swine/North Carolina/00529/2005 | | 3/10/05 |  |  |
| 984 | CY081822 | A/Swine/North Carolina/00538/2005 | | 3/10/05 |  |  |
| 985 | CY081833 | A/Swine/North Carolina/00553/2005 | | 3/22/05 |  |  |
| 986 | CY081835 | A/Swine/North Carolina/00558/2005 | | 3/25/05 |  |  |
| 987 | CY081841 | A/Swine/North Carolina/00571/2005 | | 4/22/05 |  |  |
| 988 | CY081842 | A/Swine/North Carolina/00572/2005 | | 4/22/05 |  |  |
| 989 | CY081845 | A/Swine/North Carolina/00577/2004 | | 12/22/04 |  |  |
| 990 | CY081846 | A/Swine/North Carolina/00579/2004 | | 12/22/04 |  |  |
| 991 | CY081848 | A/Swine/North Carolina/00582/2005 | | 5/5/05 |  |  |
| 992 | CY081855 | A/Swine/North Carolina/00591/2005 | | 5/5/05 | Human |  |
| 993 | CY081860 | A/Swine/North Carolina/00601/2005 | | 4/22/05 |  |  |
| 994 | CY081864 | A/Swine/North Carolina/00613/2005 | | 5/25/05 |  |  |
| 995 | CY081865 | A/Swine/North Carolina/00614/2005 | | 5/25/05 |  |  |
| 996 | CY081869 | A/Swine/North Carolina/00619/2005 | | 5/24/05 | Human |  |
| 997 | CY081870 | A/Swine/North Carolina/00621/2005 | | 1/6/05 | Human |  |
| 998 | CY081871 | A/Swine/North Carolina/00622/2005 | | 2/11/05 | Human |  |
| 999 | CY081873 | A/Swine/North Carolina/00632/2005 | | 5/19/05 | Human |  |
| 1000 | CY081874 | A/Swine/North Carolina/00636/2005 | | 5/20/05 | Human |  |
| 1001 | CY081875 | A/Swine/North Carolina/00640/2005 | | 6/3/05 |  |  |
| 1002 | CY081876 | A/Swine/North Carolina/00641/2005 | | 6/3/05 |  |  |
| 1003 | CY081880 | A/Swine/North Carolina/00655/2005 | | 6/22/05 |  |  |
| 1004 | CY081881 | A/Swine/North Carolina/00656/2005 | | 6/29/05 |  |  |
| 1005 | CY081882 | A/Swine/North Carolina/00661/2005 | | 7/1/05 |  |  |
| 1006 | CY081885 | A/Swine/North Carolina/00664/2005 | | 7/8/05 |  |  |
| 1007 | CY081886 | A/Swine/North Carolina/00665/2005 | | 7/8/05 |  |  |
| 1008 | CY081890 | A/Swine/North Carolina/00673/2005 | | 7/26/05 |  |  |
| 1009 | CY081891 | A/Swine/North Carolina/00674/2005 | | 7/26/05 | Human |  |
| 1010 | CY081892 | A/Swine/North Carolina/00675/2005 | | 7/26/05 | Human |  |
| 1011 | CY081893 | A/Swine/North Carolina/00676/2005 | | 7/26/05 | Human |  |
| 1012 | CY081894 | A/Swine/North Carolina/00677/2005 | | 7/26/05 | Human |  |
| 1013 | CY081895 | A/Swine/North Carolina/00678/2005 | | 7/26/05 | Human |  |
| 1014 | CY081896 | A/Swine/North Carolina/00679/2005 | | 7/26/05 | Human |  |
| 1015 | CY081905 | A/Swine/North Carolina/00699/2006 | | 9/14/06 | Human |  |
| 1016 | CY081906 | A/Swine/North Carolina/00700/2006 | | 9/22/06 |  |  |
| 1017 | CY081907 | A/Swine/North Carolina/00701/2006 | | 9/22/06 |  |  |
| 1018 | CY081908 | A/Swine/North Carolina/00702/2006 | |  |  |  |
| 1019 | CY081910 | A/Swine/North Carolina/00706/2005 | | 8/10/05 |  |  |
| 1020 | CY081913 | A/Swine/North Carolina/00713/2005 | | 8/25/05 |  |  |
| 1021 | CY081914 | A/Swine/North Carolina/00714/2005 | | 8/25/05 |  |  |
| 1022 | CY081916 | A/Swine/North Carolina/00716/2005 | | 8/31/05 | Human |  |
| 1023 | CY081917 | A/Swine/North Carolina/00717/2005 | | 8/31/05 |  |  |
| 1024 | CY081919 | A/Swine/North Carolina/00719/2005 | | 9/4/05 | Human |  |
| 1025 | CY081921 | A/Swine/North Carolina/00721/2005 | | 9/7/05 | Human |  |
| 1026 | CY081922 | A/Swine/North Carolina/00724/2005 | | 9/8/05 | Human |  |
| 1027 | CY081923 | A/Swine/North Carolina/00725/2005 | | 9/13/05 |  |  |
| 1028 | CY081927 | A/Swine/North Carolina/00732/2005 | | 9/16/05 |  |  |
| 1029 | CY081928 | A/Swine/North Carolina/00733/2005 | | 9/16/05 |  |  |
| 1030 | CY081929 | A/Swine/North Carolina/00734/2005 | | 9/16/05 |  |  |
| 1031 | CY081932 | A/Swine/North Carolina/00740/2005 | | 9/23/05 |  |  |
| 1032 | CY081933 | A/Swine/North Carolina/00741/2005 | | 9/23/05 |  |  |
| 1033 | CY081934 | A/Swine/North Carolina/00742/2005 | | 9/23/05 | Human |  |
| 1034 | CY081936 | A/Swine/North Carolina/00745/2005 | | 10/10/05 | Human |  |
| 1035 | CY081937 | A/Swine/North Carolina/00746/2005 | | 10/10/05 | Human |  |
| 1036 | CY081939 | A/Swine/North Carolina/00749/2005 | | 10/6/05 |  |  |
| 1037 | CY081940 | A/Swine/North Carolina/00751/2005 | | 10/6/05 | Human |  |
| 1038 | CY081941 | A/Swine/North Carolina/00754/2005 | | 10/6/05 | Human |  |
| 1039 | CY081942 | A/Swine/North Carolina/00759/2005 | | 10/6/05 |  |  |
| 1040 | CY081946 | A/Swine/North Carolina/00771/2005 | | 4/27/05 | Human |  |
| 1041 | CY081961 | A/Swine/North Carolina/00794/2005 | | 9/23/05 | Human |  |
| 1042 | CY081963 | A/Swine/North Carolina/00796/2005 | | 9/27/05 | Human |  |
| 1043 | CY081973 | A/Swine/North Carolina/00817/2005 | | 10/13/05 | Human |  |
| 1044 | CY081980 | A/Swine/North Carolina/00828/2005 | | 10/21/05 | Human |  |
| 1045 | CY081988 | A/Swine/North Carolina/00839/2005 | | 10/27/05 |  |  |
| 1046 | CY081993 | A/Swine/North Carolina/00850/2005 | | 11/8/05 | Human |  |
| 1047 | CY081994 | A/Swine/North Carolina/00852/2005 | | 11/11/05 |  |  |
| 1048 | CY081995 | A/Swine/North Carolina/00853/2005 | | 11/11/05 | Human |  |
| 1049 | CY081997 | A/Swine/North Carolina/00856/2005 | | 11/11/05 | Human |  |
| 1050 | CY082006 | A/Swine/North Carolina/00871/2005 | | 11/22/05 |  |  |
| 1051 | CY082007 | A/Swine/North Carolina/00872/2005 | | 11/23/05 |  |  |
| 1052 | CY082008 | A/Swine/North Carolina/00873/2005 | | 11/23/05 |  |  |
| 1053 | CY082009 | A/Swine/North Carolina/00874/2005 | | 11/23/05 |  |  |
| 1054 | CY082018 | A/Swine/North Carolina/00890/2005 | | 12/8/05 |  |  |
| 1055 | CY082019 | A/Swine/North Carolina/00899/2005 | | 12/8/05 |  |  |
| 1056 | CY082020 | A/Swine/North Carolina/00900/2005 | | 12/8/05 |  |  |
| 1057 | CY082021 | A/Swine/North Carolina/00901/2005 | | 12/8/05 |  |  |
| 1058 | CY082022 | A/Swine/North Carolina/00903/2005 | | 12/8/05 |  |  |
| 1059 | CY082023 | A/Swine/North Carolina/00906/2005 | | 12/8/05 |  |  |
| 1060 | CY082024 | A/Swine/North Carolina/00908/2005 | | 12/8/05 |  |  |
| 1061 | CY082025 | A/Swine/North Carolina/00910/2005 | | 12/8/05 |  |  |
| 1062 | CY082026 | A/Swine/North Carolina/00912/2005 | | 12/8/05 |  |  |
| 1063 | CY082027 | A/Swine/North Carolina/00913/2005 | | 12/8/05 |  |  |
| 1064 | CY082028 | A/Swine/North Carolina/00914/2005 | | 12/8/05 |  |  |
| 1065 | CY082029 | A/Swine/North Carolina/00919/2005 | | 12/8/05 |  |  |
| 1066 | CY082030 | A/Swine/North Carolina/00920/2005 | | 12/8/05 |  |  |
| 1067 | CY082031 | A/Swine/North Carolina/00925/2005 | | 12/13/05 |  |  |
| 1068 | CY082032 | A/Swine/North Carolina/00926/2005 | | 12/13/05 |  |  |
| 1069 | CY082033 | A/Swine/North Carolina/00927/2005 | | 12/13/05 | Human |  |
| 1070 | CY082034 | A/Swine/North Carolina/00928/2005 | | 12/15/05 |  |  |
| 1071 | CY082036 | A/Swine/North Carolina/00932/2005 | | 12/20/05 |  |  |
| 1072 | CY082045 | A/Swine/North Carolina/00947/2006 | | 9/26/06 |  |  |
| 1073 | CY082049 | A/Swine/North Carolina/00955/2006 | | 1/4/06 | Human |  |
| 1074 | CY082051 | A/Swine/North Carolina/00958/2006 | | 1/11/06 | Human |  |
| 1075 | CY082056 | A/Swine/North Carolina/00968/2006 | | 1/20/06 |  |  |
| 1076 | CY082058 | A/Swine/North Carolina/00972/2006 | | 1/27/06 |  |  |
| 1077 | CY082060 | A/Swine/North Carolina/00977/2006 | | 2/1/06 |  |  |
| 1078 | CY082061 | A/Swine/North Carolina/00979/2006 | | 2/2/06 |  |  |
| 1079 | CY082063 | A/Swine/North Carolina/00981/2006 | | 2/3/06 |  |  |
| 1080 | CY082066 | A/Swine/North Carolina/00989/2006 | | 2/7/06 |  |  |
| 1081 | CY082067 | A/Swine/North Carolina/00990/2006 | | 2/7/06 |  |  |
| 1082 | CY082073 | A/Swine/North Carolina/00997/2006 | | 2/10/06 |  |  |
| 1083 | CY082077 | A/Swine/North Carolina/01006/2006 | | 10/12/06 |  |  |
| 1084 | CY082078 | A/Swine/North Carolina/01007/2006 | | 10/12/06 | Human |  |
| 1085 | CY082079 | A/Swine/North Carolina/01008/2006 | | 10/12/06 | Human |  |
| 1086 | CY082080 | A/Swine/North Carolina/01009/2006 | | 10/12/06 | Human |  |
| 1087 | CY082086 | A/Swine/North Carolina/01018/2006 | | 3/1/06 |  |  |
| 1088 | CY082087 | A/Swine/North Carolina/01020/2006 | | 3/1/06 |  |  |
| 1089 | CY082088 | A/Swine/North Carolina/01023/2006 | | 3/1/06 |  |  |
| 1090 | CY082089 | A/Swine/North Carolina/01024/2006 | | 3/1/06 |  |  |
| 1091 | CY082090 | A/Swine/North Carolina/01025/2006 | | 3/1/06 | Human |  |
| 1092 | CY082091 | A/Swine/North Carolina/01027/2006 | | 3/1/06 |  |  |
| 1093 | CY082092 | A/Swine/North Carolina/01028/2006 | | 3/1/06 | Human |  |
| 1094 | CY082093 | A/Swine/North Carolina/01029/2006 | | 3/1/06 | Human |  |
| 1095 | CY082094 | A/Swine/North Carolina/01030/2006 | | 3/1/06 | Human |  |
| 1096 | CY082096 | A/Swine/North Carolina/01034/2006 | | 3/3/06 |  |  |
| 1097 | CY082101 | A/Swine/North Carolina/01039/2006 | | 3/3/06 |  |  |
| 1098 | CY082104 | A/Swine/North Carolina/01044/2006 | | 3/9/06 |  |  |
| 1099 | CY082105 | A/Swine/North Carolina/01045/2006 | | 3/9/06 |  |  |
| 1100 | CY082106 | A/Swine/North Carolina/01046/2006 | | 3/9/06 |  |  |
| 1101 | CY082107 | A/Swine/North Carolina/01047/2006 | | 3/9/06 | Human |  |
| 1102 | CY082108 | A/Swine/North Carolina/01048/2006 | | 3/9/06 | Human |  |
| 1103 | CY082109 | A/Swine/North Carolina/01049/2006 | | 3/9/06 | Human |  |
| 1104 | CY082110 | A/Swine/North Carolina/01050/2006 | | 3/9/06 |  |  |
| 1105 | CY082111 | A/Swine/North Carolina/01055/2006 | | 3/16/06 | Human |  |
| 1106 | CY082112 | A/Swine/North Carolina/01057/2006 | | 3/16/06 | Human |  |
| 1107 | CY082113 | A/Swine/North Carolina/01058/2006 | | 3/16/06 | Human |  |
| 1108 | CY082122 | A/Swine/North Carolina/01070/2006 | | 3/24/06 |  |  |
| 1109 | CY082123 | A/Swine/North Carolina/01071/2006 | | 3/24/06 |  |  |
| 1110 | CY082132 | A/Swine/North Carolina/01084/2006 | | 4/11/06 |  |  |
| 1111 | CY082136 | A/Swine/North Carolina/01092/2006 | | 4/11/06 |  |  |
| 1112 | CY082142 | A/Swine/North Carolina/01100/2006 | | 4/19/06 |  |  |
| 1113 | CY082144 | A/Swine/North Carolina/01102/2006 | | 4/20/06 |  |  |
| 1114 | CY082146 | A/Swine/North Carolina/01107/2006 | | 4/21/06 | Human |  |
| 1115 | CY082148 | A/Swine/North Carolina/01112/2006 | | 4/26/06 |  |  |
| 1116 | CY082150 | A/Swine/North Carolina/01115/2006 | | 5/2/06 |  |  |
| 1117 | CY082151 | A/Swine/North Carolina/01116/2006 | | 5/2/06 |  |  |
| 1118 | CY082161 | A/Swine/North Carolina/01129/2006 | | 5/5/06 | Human |  |
| 1119 | CY082163 | A/Swine/North Carolina/01132/2006 | | 5/5/06 | Human |  |
| 1120 | CY082172 | A/Swine/North Carolina/01152/2006 | | 6/1/06 |  |  |
| 1121 | CY082173 | A/Swine/North Carolina/01154/2006 | | 6/6/06 |  |  |
| 1122 | CY082174 | A/Swine/North Carolina/01155/2006 | | 6/7/06 | Human |  |
| 1123 | CY082175 | A/Swine/North Carolina/01161/2006 | | 6/14/06 | Human |  |
| 1124 | CY082177 | A/Swine/North Carolina/01165/2006 | | 6/15/06 | Human |  |
| 1125 | CY082180 | A/Swine/North Carolina/01169/2006 | | 6/22/06 | Human |  |
| 1126 | CY082181 | A/Swine/North Carolina/01172/2006 | | 6/23/06 | Human |  |
| 1127 | CY082182 | A/Swine/North Carolina/01173/2006 | | 6/23/06 |  |  |
| 1128 | CY082190 | A/Swine/North Carolina/01184/2006 | | 7/10/06 |  |  |
| 1129 | CY082196 | A/Swine/North Carolina/01197/2006 | | 8/11/06 |  |  |
| 1130 | CY082199 | A/Swine/North Carolina/01202/2006 | | 8/16/06 |  |  |
| 1131 | CY082205 | A/Swine/North Carolina/01211/2006 | | 10/4/06 |  |  |
| 1132 | CY082213 | A/Swine/North Carolina/01225/2006 | | 10/17/06 |  |  |
| 1133 | CY082214 | A/Swine/North Carolina/01226/2006 | | 10/17/06 | Human |  |
| 1134 | CY082225 | A/Swine/North Carolina/01242/2006 | | 10/12/06 | Human |  |
| 1135 | CY082228 | A/Swine/North Carolina/01247/2006 | | 10/26/06 |  |  |
| 1136 | CY082229 | A/Swine/North Carolina/01248/2006 | | 10/26/06 |  |  |
| 1137 | CY082230 | A/Swine/North Carolina/01249/2006 | | 10/26/06 |  |  |
| 1138 | CY082231 | A/Swine/North Carolina/01250/2006 | | 10/26/06 | Human |  |
| 1139 | CY082232 | A/Swine/North Carolina/01251/2006 | | 10/26/06 |  |  |
| 1140 | CY082233 | A/Swine/North Carolina/01252/2006 | | 10/26/06 | Human |  |
| 1141 | CY082234 | A/Swine/North Carolina/01253/2006 | | 10/26/06 | Human |  |
| 1142 | CY082235 | A/Swine/North Carolina/01254/2006 | | 10/26/06 |  |  |
| 1143 | CY082236 | A/Swine/North Carolina/01255/2006 | | 10/26/06 |  |  |
| 1144 | CY082237 | A/Swine/North Carolina/01256/2006 | | 10/26/06 | Human |  |
| 1145 | CY082247 | A/Swine/North Carolina/01268/2006 | | 10/17/06 |  |  |
| 1146 | CY082250 | A/Swine/North Carolina/01276/2006 | | 11/2/06 |  |  |
| 1147 | CY082256 | A/Swine/North Carolina/01285/2006 | | 11/3/06 | Human |  |
| 1148 | CY082258 | A/Swine/North Carolina/01287/2006 | | 11/8/06 |  |  |
| 1149 | CY082259 | A/Swine/North Carolina/01289/2006 | | 10/13/06 | Human |  |
| 1150 | CY082261 | A/Swine/North Carolina/01292/2006 | | 10/25/06 |  |  |
| 1151 | CY082263 | A/Swine/North Carolina/01295/2006 | | 10/31/06 |  |  |
| 1152 | CY082269 | A/Swine/North Carolina/01304/2006 | | 11/17/06 |  |  |
| 1153 | CY082277 | A/Swine/North Carolina/01316/2006 | | 11/21/06 | Human |  |
| 1154 | CY082280 | A/Swine/North Carolina/01320/2006 | | 11/14/06 |  |  |
| 1155 | CY082282 | A/Swine/North Carolina/01324/2006 | | 11/29/06 |  |  |
| 1156 | CY082283 | A/Swine/North Carolina/01325/2006 | | 11/29/06 |  |  |
| 1157 | CY082284 | A/Swine/North Carolina/01326/2006 | | 11/29/06 |  |  |
| 1158 | CY082285 | A/Swine/North Carolina/01327/2006 | | 11/29/06 |  |  |
| 1159 | CY082288 | A/Swine/North Carolina/01333/2006 | | 11/22/06 |  |  |
| 1160 | CY082292 | A/Swine/North Carolina/01345/2006 | | 11/29/06 |  |  |
| 1161 | CY082293 | A/Swine/North Carolina/01346/2006 | | 11/29/06 |  |  |
| 1162 | CY082294 | A/Swine/North Carolina/01349/2006 | | 12/8/06 |  |  |
| 1163 | CY082295 | A/Swine/North Carolina/01350/2006 | | 12/8/06 |  |  |
| 1164 | CY082296 | A/Swine/North Carolina/01351/2006 | | 12/8/06 |  |  |
| 1165 | CY082302 | A/Swine/North Carolina/01357/2006 | | 12/12/06 |  |  |
| 1166 | CY082307 | A/Swine/North Carolina/01363/2006 | | 12/7/06 |  |  |
| 1167 | CY082308 | A/Swine/North Carolina/01364/2006 | | 12/7/06 |  |  |
| 1168 | CY082309 | A/Swine/North Carolina/01365/2006 | | 12/7/06 |  |  |
| 1169 | CY082323 | A/Swine/North Carolina/01395/2006 | | 12/29/06 |  |  |
| 1170 | CY082324 | A/Swine/North Carolina/01396/2006 | | 12/29/06 |  |  |
| 1171 | CY082325 | A/Swine/North Carolina/01397/2006 | | 12/29/06 |  |  |
| 1172 | CY082337 | A/Swine/North Carolina/01416/2006 | | 12/29/06 |  |  |
| 1173 | CY082340 | A/Swine/North Carolina/01424/2007 | | 1/16/07 |  |  |
| 1174 | CY082365 | A/Swine/North Carolina/01471/2007 | | 2/27/07 |  |  |
| 1175 | CY082368 | A/Swine/North Carolina/01493/2007 | | 3/8/07 | Human |  |
| 1176 | CY082369 | A/Swine/North Carolina/01507/2006 | | 11/7/06 |  |  |
| 1177 | CY082370 | A/Swine/North Carolina/01512/2007 | | 1/23/07 |  |  |
| 1178 | CY082371 | A/Swine/North Carolina/01513/2007 | | 1/25/07 |  |  |
| 1179 | CY082372 | A/Swine/North Carolina/01514/2007 | | 1/26/07 |  |  |
| 1180 | CY082373 | A/Swine/North Carolina/01515/2007 | | 2/2/07 |  |  |
| 1181 | CY082374 | A/Swine/North Carolina/01517/2007 | | 2/8/07 |  |  |
| 1182 | CY082375 | A/Swine/North Carolina/01518/2007 | | 2/15/07 |  |  |
| 1183 | CY082378 | A/Swine/North Carolina/01523/2007 | | 3/13/07 |  |  |
| 1184 | CY082389 | A/Swine/North Carolina/01559/2007 | | 4/19/07 |  |  |
| 1185 | CY082390 | A/Swine/North Carolina/01562/2007 | | 4/17/07 |  |  |
| 1186 | CY082392 | A/Swine/North Carolina/01565/2006 | | 11/21/06 |  |  |
| 1187 | CY082398 | A/Swine/North Carolina/01580/2007 | | 4/26/07 |  |  |
| 1188 | CY082401 | A/Swine/North Carolina/01591/2007 | | 4/24/07 |  |  |
| 1189 | CY082417 | A/Swine/North Carolina/01636/2007 | | 6/5/07 |  |  |
| 1190 | CY082431 | A/Swine/North Carolina/01675/2007 | | 7/18/07 | Human |  |
| 1191 | CY082432 | A/Swine/North Carolina/01676/2007 | | 7/18/07 |  |  |
| 1192 | CY082433 | A/Swine/North Carolina/01677/2007 | | 7/18/07 |  |  |
| 1193 | CY082434 | A/Swine/North Carolina/01678/2007 | | 7/18/07 |  |  |
| 1194 | CY082439 | A/Swine/North Carolina/01685/2007 | | 7/18/07 |  |  |
| 1195 | CY082440 | A/Swine/North Carolina/01687/2007 | | 7/18/07 |  |  |
| 1196 | CY082450 | A/Swine/North Carolina/01721/2007 | | 8/17/07 |  |  |
| 1197 | CY082458 | A/Swine/North Carolina/01733/2007 | | 8/30/07 |  |  |
| 1198 | CY082459 | A/Swine/North Carolina/01734/2007 | | 8/30/07 |  |  |
| 1199 | CY082465 | A/Swine/North Carolina/01748/2007 | | 8/28/07 |  |  |
| 1200 | CY082474 | A/Swine/North Carolina/01773/2007 | | 9/20/07 |  |  |
| 1201 | CY082481 | A/Swine/North Carolina/01793/2007 | | 10/9/07 |  |  |
| 1202 | CY082486 | A/Swine/North Carolina/01808/2007 | | 10/18/07 |  |  |
| 1203 | CY082491 | A/Swine/North Carolina/01821/2007 | | 10/18/07 |  |  |
| 1204 | CY082513 | A/Swine/North Carolina/01852/2007 | | 11/7/07 |  |  |
| 1205 | CY082514 | A/Swine/North Carolina/01853/2007 | | 11/7/07 |  |  |
| 1206 | CY082516 | A/Swine/North Carolina/01855/2007 | | 11/14/07 |  |  |
| 1207 | CY082517 | A/Swine/North Carolina/01856/2007 | | 11/14/07 |  |  |
| 1208 | CY082524 | A/Swine/North Carolina/01865/2007 | | 11/29/07 |  |  |
| 1209 | CY082525 | A/Swine/North Carolina/01867/2007 | | 11/29/07 |  |  |
| 1210 | CY082526 | A/Swine/North Carolina/01868/2007 | | 11/29/07 |  |  |
| 1211 | CY082527 | A/Swine/North Carolina/01869/2007 | | 11/29/07 |  |  |
| 1212 | CY082532 | A/Swine/North Carolina/01877/2007 | | 11/28/07 |  |  |
| 1213 | CY082535 | A/Swine/North Carolina/01880/2007 | | 11/30/07 |  |  |
| 1214 | CY082555 | A/Swine/North Carolina/01910/2007 | | 12/4/07 |  |  |
| 1215 | CY082564 | A/Swine/North Carolina/01925/2007 | | 2/16/07 |  |  |
| 1216 | CY082565 | A/Swine/North Carolina/01926/2007 | | 4/5/07 |  |  |
| 1217 | CY082566 | A/Swine/North Carolina/01932/2007 | | 9/6/07 |  |  |
| 1218 | CY082567 | A/Swine/North Carolina/01935/2007 | | 10/18/07 |  |  |
| 1219 | CY082568 | A/Swine/North Carolina/01936/2007 | | 10/25/07 |  |  |
| 1220 | CY082569 | A/Swine/North Carolina/01937/2007 | | 11/8/07 |  |  |
| 1221 | CY082571 | A/Swine/North Carolina/01939/2007 | | 12/6/07 |  |  |
| 1222 | CY082577 | A/Swine/North Carolina/01946/2007 | | 12/20/07 |  |  |
| 1223 | CY082578 | A/Swine/North Carolina/01947/2007 | | 12/21/07 |  |  |
| 1224 | CY082580 | A/Swine/North Carolina/01949/2007 | | 7/17/07 |  |  |
| 1225 | CY082582 | A/Swine/North Carolina/01954/2007 | | 12/12/07 |  |  |
| 1226 | CY082585 | A/Swine/North Carolina/01957/2007 | | 12/20/07 |  |  |
| 1227 | CY082590 | A/Swine/North Carolina/01966/2007 | | 12/14/07 |  |  |
| 1228 | CY082593 | A/Swine/North Carolina/01970/2008 | | 1/3/08 |  |  |
| 1229 | CY082594 | A/Swine/North Carolina/01971/2008 | | 1/4/08 |  |  |
| 1230 | CY082595 | A/Swine/North Carolina/01972/2008 | | 1/4/08 |  |  |
| 1231 | CY082607 | A/Swine/North Carolina/01994/2008 | | 1/31/08 |  |  |
| 1232 | CY082608 | A/Swine/North Carolina/01995/2008 | | 1/31/08 |  |  |
| 1233 | CY082609 | A/Swine/North Carolina/01996/2008 | | 1/31/08 |  |  |
| 1234 | CY082615 | A/Swine/North Carolina/02005/2008 | | 2/6/08 |  |  |
| 1235 | CY082617 | A/Swine/North Carolina/02008/2008 | | 2/8/08 | Human |  |
| 1236 | CY082618 | A/Swine/North Carolina/02009/2008 | | 2/8/08 |  |  |
| 1237 | CY082628 | A/Swine/North Carolina/02023/2008 | | 2/12/08 |  |  |
| 1238 | CY082635 | A/Swine/North Carolina/02035/2008 | | 2/7/08 |  |  |
| 1239 | CY082645 | A/Swine/North Carolina/02045/2008 | | 2/22/08 |  |  |
| 1240 | CY082668 | A/Swine/North Carolina/02075/2008 | | 3/18/08 |  |  |
| 1241 | CY082669 | A/Swine/North Carolina/02080/2008 | | 3/19/08 |  |  |
| 1242 | CY082672 | A/Swine/North Carolina/02084/2008 | | 3/7/08 | Human |  |
| 1243 | CY082675 | A/Swine/North Carolina/02088/2008 | | 3/25/08 |  |  |
| 1244 | CY082680 | A/Swine/North Carolina/02095/2008 | | 3/27/08 |  |  |
| 1245 | CY082697 | A/Swine/North Carolina/02117/2008 | | 4/4/08 |  |  |
| 1246 | CY082703 | A/Swine/North Carolina/02123/2008 | | 4/15/08 |  |  |
| 1247 | CY082714 | A/Swine/North Carolina/02141/2008 | | 4/17/08 |  |  |
| 1248 | CY082724 | A/Swine/North Carolina/02161/2008 | | 3/6/08 |  |  |
| 1249 | CY082738 | A/Swine/North Carolina/02187/2008 | | 5/16/08 |  |  |
| 1250 | CY082739 | A/Swine/North Carolina/02188/2008 | | 5/16/08 |  |  |
| 1251 | CY082749 | A/Swine/North Carolina/02203/2008 | | 6/5/08 | Human |  |
| 1252 | CY082762 | A/Swine/North Carolina/02228/2008 | | 6/27/08 |  |  |
| 1253 | CY082772 | A/Swine/North Carolina/02249/2008 | | 7/30/08 | Human |  |
| 1254 | CY082784 | A/Swine/North Carolina/02269/2008 | | 8/12/08 | Human |  |
| 1255 | CY082808 | A/Swine/North Carolina/02303/2008 | | 6/18/08 |  |  |
| 1256 | CY082810 | A/Swine/North Carolina/02308/2008 | | 9/12/08 | Human |  |
| 1257 | CY082828 | A/Swine/North Carolina/02336/2008 | | 10/9/08 | Human |  |
| 1258 | CY082829 | A/Swine/North Carolina/02337/2008 | | 10/9/08 | Human |  |
| 1259 | CY082832 | A/Swine/North Carolina/02342/2008 | | 9/5/08 | Human |  |
| 1260 | CY082843 | A/Swine/North Carolina/02356/2008 | | 9/30/08 |  |  |
| 1261 | CY082871 | A/Swine/North Carolina/02403/2008 | | 9/12/08 | Human |  |
| 1262 | CY082875 | A/Swine/North Carolina/02408/2008 | | 10/28/08 |  |  |
| 1263 | CY082876 | A/Swine/North Carolina/02409/2008 | | 10/28/08 | Human |  |
| 1264 | CY082886 | A/Swine/North Carolina/02421/2008 | | 10/28/08 | Human |  |
| 1265 | CY082899 | A/Swine/North Carolina/02441/2008 | | 11/4/08 |  |  |
| 1266 | CY082901 | A/Swine/North Carolina/02444/2008 | | 11/5/08 |  |  |
| 1267 | CY082909 | A/Swine/North Carolina/02452/2008 | | 11/20/08 |  |  |
| 1268 | CY082919 | A/Swine/North Carolina/02464/2008 | | 12/4/08 | Human |  |
| 1269 | CY082920 | A/Swine/North Carolina/02465/2008 | | 12/4/08 | Human |  |
| 1270 | CY082929 | A/Swine/North Carolina/02477/2008 | | 12/18/08 |  |  |
| 1271 | CY082930 | A/Swine/North Carolina/02478/2008 | | 12/18/08 |  |  |
| 1272 | CY082937 | A/Swine/North Carolina/02488/2008 | | 12/18/08 | Human |  |
| 1273 | CY082938 | A/Swine/North Carolina/02490/2008 | | 12/18/08 |  |  |
| 1274 | CY082947 | A/Swine/North Carolina/02511/2008 | | 12/4/08 | Human |  |
| 1275 | CY082952 | A/Swine/North Carolina/02523/2008 | | 10/31/08 |  |  |
| 1276 | CY082955 | A/Swine/North Carolina/16980-1/2008 | | |  |  |
| 1277 | CY082956 | A/Swine/North Carolina/16980-11/2008 | | |  |  |
| 1278 | CY082958 | A/Swine/North Carolina/16980-4/2008 | | |  |  |
| 1279 | CY082962 | A/Swine/North Carolina/16980-8/2008 | | | Human |  |
| 1280 | [CY040494](http://www.ncbi.nlm.nih.gov/entrez/viewer.fcgi??db=nucleotide&val=CY040494) | A/Swine/North Carolina/225-12/2008 | 11/14/08 | |  |  |
| 1281 | [CY040495](http://www.ncbi.nlm.nih.gov/entrez/viewer.fcgi??db=nucleotide&val=CY040495) | A/Swine/North Carolina/225-13/2008 | 11/14/08 | |  |  |
| 1282 | [CY040496](http://www.ncbi.nlm.nih.gov/entrez/viewer.fcgi??db=nucleotide&val=CY040496) | A/Swine/North Carolina/225-2/2008 | 11/7/08 | |  |  |
| 1283 | [CY040497](http://www.ncbi.nlm.nih.gov/entrez/viewer.fcgi??db=nucleotide&val=CY040497) | A/Swine/North Carolina/225-6/2008 | 11/11/08 | |  |  |
| 1284 | [CY040499](http://www.ncbi.nlm.nih.gov/entrez/viewer.fcgi??db=nucleotide&val=CY040499) | A/Swine/North Carolina/63607-10/2008 | 10/7/08 | |  |  |
| 1285 | [CY040500](http://www.ncbi.nlm.nih.gov/entrez/viewer.fcgi??db=nucleotide&val=CY040500) | A/Swine/North Carolina/63607-14/2008 | 10/9/08 | |  |  |
| 1286 | [CY040501](http://www.ncbi.nlm.nih.gov/entrez/viewer.fcgi??db=nucleotide&val=CY040501) | A/Swine/North Carolina/63607-24/2008 | 10/17/08 | |  |  |
| 1287 | [CY040502](http://www.ncbi.nlm.nih.gov/entrez/viewer.fcgi??db=nucleotide&val=CY040502) | A/Swine/North Carolina/63607-25/2008 | 10/22/08 | |  |  |
| 1288 | [CY040503](http://www.ncbi.nlm.nih.gov/entrez/viewer.fcgi??db=nucleotide&val=CY040503) | A/Swine/North Carolina/63607-27/2008 | 10/23/08 | | Human |  |
| 1289 | [CY040504](http://www.ncbi.nlm.nih.gov/entrez/viewer.fcgi??db=nucleotide&val=CY040504) | A/Swine/North Carolina/63607-28/2008 | 10/23/08 | |  |  |
| 1290 | [CY040505](http://www.ncbi.nlm.nih.gov/entrez/viewer.fcgi??db=nucleotide&val=CY040505) | A/Swine/North Carolina/63607-37/2008 | 10/31/08 | |  |  |
| 1291 | [CY040507](http://www.ncbi.nlm.nih.gov/entrez/viewer.fcgi??db=nucleotide&val=CY040507) | A/Swine/North Carolina/63607-39/2008 | 10/31/08 | |  |  |
| 1292 | [CY040508](http://www.ncbi.nlm.nih.gov/entrez/viewer.fcgi??db=nucleotide&val=CY040508) | A/Swine/North Carolina/63607-8/2008 | 10/7/08 | |  |  |
| 1293 | [CY040509](http://www.ncbi.nlm.nih.gov/entrez/viewer.fcgi??db=nucleotide&val=CY040509) | A/Swine/North Carolina/63607-9/2008 | 10/7/08 | |  |  |
| 1294 | ADB56964 | A/Swine/North/Carolina/02921/2009 |  | |  |  |
| 1295 | ADC80755 | A/Swine/North/Carolina/02926/2009 |  | |  |  |
| 1296 | CY081967 | A/Swine/Ohio/00806/2005 | 10/11/05 | |  |  |
| 1297 | CY082068 | A/Swine/Ohio/00991/2006 | 2/7/06 | | Human |  |
| 1298 | CY082115 | A/Swine/Ohio/01061/2006 | 3/22/06 | |  |  |
| 1299 | CY082124 | A/Swine/Ohio/01072/2006 | 3/24/06 | |  |  |
| 1300 | CY082125 | A/Swine/Ohio/01073/2006 | 3/28/06 | |  |  |
| 1301 | CY082170 | A/Swine/Ohio/01150/2006 | 5/25/06 | |  |  |
| 1302 | CY082211 | A/Swine/Ohio/01221/2006 | 10/10/06 | |  |  |
| 1303 | CY082249 | A/Swine/Ohio/01273/2006 | 10/27/06 | |  |  |
| 1304 | CY082339 | A/Swine/Ohio/01422/2007 | 1/11/07 | |  |  |
| 1305 | CY082556 | A/Swine/Ohio/01911/2007 | 12/4/07 | |  |  |
| 1306 | CY082596 | A/Swine/Ohio/01973/2008 | 1/8/08 | |  |  |
| 1307 | CY082599 | A/Swine/Ohio/01977/2008 | 1/9/08 | |  |  |
| 1308 | CY082613 | A/Swine/Ohio/02001/2008 | 1/29/08 | |  |  |
| 1309 | CY082629 | A/Swine/Ohio/02024/2008 | 2/13/08 | |  |  |
| 1310 | CY082631 | A/Swine/Ohio/02026/2008 | 2/15/08 | |  |  |
| 1311 | CY082638 | A/Swine/Ohio/02038/2008 | 2/13/08 | |  |  |
| 1312 | CY082676 | A/Swine/Ohio/02090/2008 | 3/27/08 | |  |  |
| 1313 | CY082699 | A/Swine/Ohio/02119/2008 | 4/10/08 | |  |  |
| 1314 | CY082744 | A/Swine/Ohio/02195/2008 | 5/30/08 | |  |  |
| 1315 | CY082809 | A/Swine/Ohio/02305/2008 | 8/21/08 | |  |  |
| 1316 | CY082823 | A/Swine/Ohio/02327/2008 | 9/17/08 | |  |  |
| 1317 | CY082860 | A/Swine/Ohio/02382/2008 | 10/16/08 | |  |  |
| 1318 | CY082861 | A/Swine/Ohio/02383/2008 | 10/16/08 | |  |  |
| 1319 | CY082918 | A/Swine/Ohio/02462/2008 | 12/3/08 | |  |  |
| 1320 | CY082950 | A/Swine/Ohio/02521/2008 | 9/25/08 | | Human |  |
| 1321 | CY082951 | A/Swine/Ohio/02522/2008 | 9/25/08 | | Human |  |
| 1322 | ADD21434 | A/Swine/Ohio/02973/2010 | 1/20/10 | |  |  |
| 1323 | CY082959 | A/Swine/Ohio/16980-5/2008 |  | | Human |  |
| 1324 | [CY040510](http://www.ncbi.nlm.nih.gov/entrez/viewer.fcgi??db=nucleotide&val=CY040510) | A/Swine/Ohio/63607-32/2008 | 10/28/08 | |  |  |
| 1325 | CY081624 | A/Swine/Oklahoma/00130/2003 | 8/12/03 | | Human |  |
| 1326 | CY081671 | A/Swine/Oklahoma/00234/2003 | 12/23/03 | | Human |  |
| 1327 | CY081688 | A/Swine/Oklahoma/00259/2004 | 3/3/04 | |  |  |
| 1328 | CY081774 | A/Swine/Oklahoma/00429/2004 | 11/5/04 | |  |  |
| 1329 | CY081812 | A/Swine/Oklahoma/00507/2005 | 3/15/05 | |  |  |
| 1330 | CY081849 | A/Swine/Oklahoma/00583/2005 | 5/4/05 | |  |  |
| 1331 | CY081851 | A/Swine/Oklahoma/00585/2005 | 5/4/05 | |  |  |
| 1332 | CY081859 | A/Swine/Oklahoma/00598/2005 | 1/20/05 | |  |  |
| 1333 | CY081935 | A/Swine/Oklahoma/00743/2005 | 9/23/05 | | Human |  |
| 1334 | CY081953 | A/Swine/Oklahoma/00786/2005 | 9/23/05 | |  |  |
| 1335 | CY081954 | A/Swine/Oklahoma/00787/2005 | 9/23/05 | |  |  |
| 1336 | CY081955 | A/Swine/Oklahoma/00788/2005 | 9/23/05 | | Human |  |
| 1337 | CY081956 | A/Swine/Oklahoma/00789/2005 | 9/23/05 | | Human |  |
| 1338 | CY081957 | A/Swine/Oklahoma/00790/2005 | 9/23/05 | |  |  |
| 1339 | CY081958 | A/Swine/Oklahoma/00791/2005 | 9/23/05 | |  |  |
| 1340 | CY081959 | A/Swine/Oklahoma/00792/2005 | 9/23/05 | |  |  |
| 1341 | CY081960 | A/Swine/Oklahoma/00793/2005 | 9/23/05 | |  |  |
| 1342 | CY081965 | A/Swine/Oklahoma/00801/2005 | 10/4/05 | |  |  |
| 1343 | CY081966 | A/Swine/Oklahoma/00802/2005 | 10/4/05 | |  |  |
| 1344 | CY081971 | A/Swine/Oklahoma/00813/2005 | 10/12/05 | |  |  |
| 1345 | CY081975 | A/Swine/Oklahoma/00820/2005 | 10/14/05 | |  |  |
| 1346 | CY081976 | A/Swine/Oklahoma/00821/2005 | 10/14/05 | | Human |  |
| 1347 | CY081981 | A/Swine/Oklahoma/00829/2005 | 10/21/05 | | Human |  |
| 1348 | CY081983 | A/Swine/Oklahoma/00833/2005 | 10/25/05 | |  |  |
| 1349 | CY081984 | A/Swine/Oklahoma/00834/2005 | 10/25/05 | |  |  |
| 1350 | CY081991 | A/Swine/Oklahoma/00846/2005 | 11/3/05 | |  |  |
| 1351 | CY082001 | A/Swine/Oklahoma/00864/2005 | 11/17/05 | |  |  |
| 1352 | CY082002 | A/Swine/Oklahoma/00865/2005 | 11/17/05 | |  |  |
| 1353 | CY082011 | A/Swine/Oklahoma/00879/2005 | 12/1/05 | |  |  |
| 1354 | CY082015 | A/Swine/Oklahoma/00883/2005 | 12/2/05 | |  |  |
| 1355 | CY082048 | A/Swine/Oklahoma/00954/2006 | 1/4/06 | |  |  |
| 1356 | CY082057 | A/Swine/Oklahoma/00971/2006 | 1/24/06 | |  |  |
| 1357 | ACV42128 | A/Swine/Oklahoma/010226-16/2008 |  | | Human (7) | ACV42124 |
| 1358 | ACV42139 | A/Swine/Oklahoma/010226-17/2008 |  | | Human (7) | ACV42135 |
| 1359 | CY082102 | A/Swine/Oklahoma/01042/2006 | 3/7/06 | |  |  |
| 1360 | CY082103 | A/Swine/Oklahoma/01043/2006 | 3/7/06 | |  |  |
| 1361 | ACV42161 | A/Swine/Oklahoma/010710-8/2008 |  | | Human (7) | ACV42157 |
| 1362 | ACV42150 | A/Swine/Oklahoma/010710-9/2008 |  | | Human (7) | ACV42146 |
| 1363 | CY082129 | A/Swine/Oklahoma/01078/2006 | 4/4/06 | |  |  |
| 1364 | CY082152 | A/Swine/Oklahoma/01117/2006 | 5/2/06 | |  |  |
| 1365 | CY082153 | A/Swine/Oklahoma/01118/2006 | 5/2/06 | | Human |  |
| 1366 | CY082160 | A/Swine/Oklahoma/01128/2006 | 5/5/06 | | Human |  |
| 1367 | ACV42183 | A/Swine/Oklahoma/011289-10/2008 |  | | Human (7) | ACV42179 |
| 1368 | ACV42194 | A/Swine/Oklahoma/011289-8/2008 |  | | Human (7) | ACV42190 |
| 1369 | ACV42172 | A/Swine/Oklahoma/011289-9/2008 |  | | Human (7) | ACV42168 |
| 1370 | CY082168 | A/Swine/Oklahoma/01139/2006 | 5/17/06 | | Human |  |
| 1371 | ACV42216 | A/Swine/Oklahoma/011521-4/2008 |  | | Human (7) | ACV42212 |
| 1372 | ACV42205 | A/Swine/Oklahoma/011521-5/2008 |  | | Human (7) | ACV42201 |
| 1373 | CY082210 | A/Swine/Oklahoma/01220/2006 | 10/10/06 | | Human |  |
| 1374 | CY082226 | A/Swine/Oklahoma/01245/2006 | 10/18/06 | | Human |  |
| 1375 | CY082253 | A/Swine/Oklahoma/01282/2006 | 10/25/06 | | Human |  |
| 1376 | CY082266 | A/Swine/Oklahoma/01301/2006 | 11/15/06 | | Human |  |
| 1377 | CY082321 | A/Swine/Oklahoma/01382/2006 | 12/8/06 | | Human |  |
| 1378 | CY082354 | A/Swine/Oklahoma/01452/2007 | 1/26/07 | | Human |  |
| 1379 | CY082367 | A/Swine/Oklahoma/01477/2007 | 2/20/07 | |  |  |
| 1380 | CY082397 | A/Swine/Oklahoma/01576/2007 | 4/19/07 | | Human |  |
| 1381 | ACV42227 | A/Swine/Oklahoma/016179-8/2008 |  | | Human (7) | ACV42223 |
| 1382 | ACV42238 | A/Swine/Oklahoma/016179-9/2008 |  | | Human (7) | ACV42234 |
| 1383 | CY082422 | A/Swine/Oklahoma/01654/2007 | 6/15/07 | |  |  |
| 1384 | CY082441 | A/Swine/Oklahoma/01688/2007 | 7/19/07 | |  |  |
| 1385 | CY082453 | A/Swine/Oklahoma/01725/2007 | 8/22/07 | |  |  |
| 1386 | CY082463 | A/Swine/Oklahoma/01744/2007 | 8/24/07 | |  |  |
| 1387 | CY082471 | A/Swine/Oklahoma/01767/2007 | 9/12/07 | |  |  |
| 1388 | CY082492 | A/Swine/Oklahoma/01823/2007 | 10/26/07 | |  |  |
| 1389 | CY082494 | A/Swine/Oklahoma/01825/2007 | 10/31/07 | |  |  |
| 1390 | CY082497 | A/Swine/Oklahoma/01828/2007 | 10/26/07 | |  |  |
| 1391 | CY082498 | A/Swine/Oklahoma/01829/2007 | 10/25/07 | |  |  |
| 1392 | CY082575 | A/Swine/Oklahoma/01944/2007 | 12/14/07 | |  |  |
| 1393 | CY082603 | A/Swine/Oklahoma/01984/2008 | 1/24/08 | |  |  |
| 1394 | CY082612 | A/Swine/Oklahoma/02000/2008 | 1/24/08 | |  |  |
| 1395 | CY082642 | A/Swine/Oklahoma/02042/2008 | 2/21/08 | |  |  |
| 1396 | CY082652 | A/Swine/Oklahoma/02056/2008 | 2/29/08 | |  |  |
| 1397 | CY082653 | A/Swine/Oklahoma/02058/2008 | 3/4/08 | |  |  |
| 1398 | CY082657 | A/Swine/Oklahoma/02062/2008 | 3/7/08 | |  |  |
| 1399 | CY082658 | A/Swine/Oklahoma/02063/2008 | 3/11/08 | |  |  |
| 1400 | ACV42249 | A/Swine/Oklahoma/020734-2/2008 |  | | Human (7) | ACV42245 |
| 1401 | ACV42260 | A/Swine/Oklahoma/020734-3/2008 |  | | Human (7) | ACV42256 |
| 1402 | ACV42282 | A/Swine/Oklahoma/020736-1/2008 |  | | Human (7) | ACV42278 |
| 1403 | ACV42271 | A/Swine/Oklahoma/020736-2/2008 |  | | Human (7) | ACV42267 |
| 1404 | CY082671 | A/Swine/Oklahoma/02083/2008 | 3/6/08 | | Human |  |
| 1405 | CY082690 | A/Swine/Oklahoma/02107/2008 | 4/2/08 | | Human |  |
| 1406 | CY082713 | A/Swine/Oklahoma/02140/2008 | 4/2/08 | | Human |  |
| 1407 | CY082725 | A/Swine/Oklahoma/02162/2008 | 4/24/08 | |  |  |
| 1408 | CY082726 | A/Swine/Oklahoma/02163/2008 | 4/24/08 | | Human |  |
| 1409 | CY082729 | A/Swine/Oklahoma/02170/2008 | 4/24/08 | | Human |  |
| 1410 | CY082737 | A/Swine/Oklahoma/02186/2008 | 5/14/08 | | Human |  |
| 1411 | CY082751 | A/Swine/Oklahoma/02211/2008 | 6/11/08 | | Human |  |
| 1412 | CY082755 | A/Swine/Oklahoma/02219/2008 | 6/25/08 | | Human |  |
| 1413 | CY082776 | A/Swine/Oklahoma/02257/2008 | 7/31/08 | | Human |  |
| 1414 | CY082786 | A/Swine/Oklahoma/02271/2008 | 8/14/08 | | Human |  |
| 1415 | CY082864 | A/Swine/Oklahoma/02386/2008 | 10/22/08 | | Human |  |
| 1416 | CY082872 | A/Swine/Oklahoma/02404/2008 | 10/10/08 | | Human |  |
| 1417 | CY082949 | A/Swine/Oklahoma/02520/2008 | 9/16/08 | | Human |  |
| 1418 | ACV42029 | A/Swine/Oklahoma/032726/2008 |  | | Human | ACV42025 |
| 1419 | ACV42293 | A/Swine/Oklahoma/042169/2008 |  | | Human | ACV42289 |
| 1420 | ACV42062 | A/Swine/Oklahoma/053259/2008 |  | | Human | ACV42058 |
| 1421 | [CY040512](http://www.ncbi.nlm.nih.gov/entrez/viewer.fcgi??db=nucleotide&val=CY040512) | A/Swine/Oklahoma/63607-6/2008 | 10/7/08 | | Human |  |
| 1422 | ABB86929 | A/Swine/Ontario/48235/04/H1N2 |  | | Human |  |
| 1423 | ABB86917 | A/Swine/Ontario/52156/03/H1N2 |  | | Human |  |
| 1424 | ABB86899 | A/Swine/Ontario/55383/04/H1N2 |  | | Human |  |
| 1425 | ADD64908 | A/Swine/OR/10-004060/2009 |  | |  |  |
| 1426 | CY082117 | A/Swine/Pennsylvania/01064/2006 | 3/22/06 | |  |  |
| 1427 | CY082468 | A/Swine/Quebec/01758/2007 | 9/6/07 | |  |  |
| 1428 | CY082469 | A/Swine/Quebec/01759/2007 | 9/7/07 | |  |  |
| 1429 | CY082563 | A/Swine/Quebec/01924/2007 | 11/28/07 | |  |  |
| 1430 | CY082917 | A/Swine/Quebec/02461/2008 | 12/3/08 | |  |  |
| 1431 | CY082597 | A/Swine/Saskatchewan/01974/2008 |  | |  |  |
| 1432 | ADK65103 | A/Swine/SD/1/2010 | 4/27/10 | |  |  |
| 1433 | ADB82970 | A/Swine/SD/31813/2009 | 11/20/09 | | Human |  |
| 1434 | CY082335 | A/Swine/South Carolina/01414/2006 | 12/29/06 | | Human |  |
| 1435 | CY081863 | A/Swine/South Dakota/00612/2005 | 5/20/05 | | Human |  |
| 1436 | CY081926 | A/Swine/South Dakota/00731/2005 | 9/14/05 | |  |  |
| 1437 | CY081952 | A/Swine/South Dakota/00783/2005 | 9/21/05 | |  |  |
| 1438 | CY082193 | A/Swine/South Dakota/01189/2006 | 7/26/06 | | Human |  |
| 1439 | CY082444 | A/Swine/South Dakota/01696/2007 | 8/8/07 | |  |  |
| 1440 | CY082445 | A/Swine/South Dakota/01697/2007 | 8/8/07 | |  |  |
| 1441 | CY082647 | A/Swine/South Dakota/02048/2008 | 2/27/08 | |  |  |
| 1442 | CY082753 | A/Swine/South Dakota/02216/2008 | 6/17/08 | |  |  |
| 1443 | CY082827 | A/Swine/South Dakota/02333/2008 | 10/8/08 | |  |  |
| 1444 | CY082866 | A/Swine/South Dakota/02389/2008 | 10/24/08 | |  |  |
| 1445 | CY081691 | A/Swine/Tennessee/00264/2004 | 3/11/04 | | Human |  |
| 1446 | CY081648 | A/Swine/Texas/00195/2003 | 10/15/03 | |  |  |
| 1447 | CY081679 | A/Swine/Texas/00244/2004 | 2/4/04 | |  |  |
| 1448 | CY081693 | A/Swine/Texas/00266/2004 | 3/17/04 | |  |  |
| 1449 | CY081817 | A/Swine/Texas/00525/2005 | 3/8/05 | |  |  |
| 1450 | CY081818 | A/Swine/Texas/00526/2005 | 3/9/05 | |  |  |
| 1451 | CY081839 | A/Swine/Texas/00562/2005 | 3/30/05 | |  |  |
| 1452 | CY081887 | A/Swine/Texas/00669/2005 | 7/14/05 | |  |  |
| 1453 | CY081888 | A/Swine/Texas/00670/2005 | 7/14/05 | |  |  |
| 1454 | CY081972 | A/Swine/Texas/00815/2005 | 10/12/05 | |  |  |
| 1455 | ACV42117 | A/Swine/Texas/008648/2008 |  | | Human | ACV42113 |
| 1456 | CY082003 | A/Swine/Texas/00867/2005 | 11/17/05 | |  |  |
| 1457 | CY082047 | A/Swine/Texas/00952/2006 | 9/28/06 | |  |  |
| 1458 | CY082215 | A/Swine/Texas/01229/2006 | 9/26/06 | |  |  |
| 1459 | CY082216 | A/Swine/Texas/01230/2006 | 9/26/06 | |  |  |
| 1460 | CY082217 | A/Swine/Texas/01231/2006 | 9/26/06 | |  |  |
| 1461 | CY082218 | A/Swine/Texas/01232/2006 | 9/26/06 | |  |  |
| 1462 | CY082219 | A/Swine/Texas/01233/2006 | 9/26/06 | | Human |  |
| 1463 | CY082220 | A/Swine/Texas/01234/2006 | 9/26/06 | | Human |  |
| 1464 | CY082221 | A/Swine/Texas/01235/2006 | 9/26/06 | | Human |  |
| 1465 | CY082222 | A/Swine/Texas/01236/2006 | 9/26/06 | | Human |  |
| 1466 | CY082224 | A/Swine/Texas/01238/2006 | 9/28/06 | | Human |  |
| 1467 | CY082273 | A/Swine/Texas/01308/2006 | 11/2/06 | | Human |  |
| 1468 | CY082299 | A/Swine/Texas/01354/2006 | 12/4/06 | | Human |  |
| 1469 | CY082333 | A/Swine/Texas/01412/2006 | 12/19/06 | | Human |  |
| 1470 | CY082334 | A/Swine/Texas/01413/2006 | 12/20/06 | |  |  |
| 1471 | CY082377 | A/Swine/Texas/01522/2007 | 3/13/07 | |  |  |
| 1472 | CY082425 | A/Swine/Texas/01657/2007 | 6/21/07 | | Human |  |
| 1473 | CY082426 | A/Swine/Texas/01658/2007 | 6/21/07 | | Human |  |
| 1474 | CY082490 | A/Swine/Texas/01820/2007 | 10/18/07 | | Human |  |
| 1475 | CY082493 | A/Swine/Texas/01824/2007 | 10/26/07 | |  |  |
| 1476 | CY082598 | A/Swine/Texas/01976/2008 | 1/9/08 | |  |  |
| 1477 | CY082641 | A/Swine/Texas/02041/2008 | 2/20/08 | |  |  |
| 1478 | CY082777 | A/Swine/Texas/02259/2008 | 8/8/08 | |  |  |
| 1479 | CY082838 | A/Swine/Texas/02351/2008 | 9/26/08 | |  |  |
| 1480 | CY082839 | A/Swine/Texas/02352/2008 | 9/26/08 | | Human | ADM18122 |
| 1481 | CY082840 | A/Swine/Texas/02353/2008 | 9/26/08 | | Human |  |
| 1482 | CY082841 | A/Swine/Texas/02354/2008 | 9/26/08 | |  |  |
| 1483 | CY082842 | A/Swine/Texas/02355/2008 | 9/26/08 | | Human |  |
| 1484 | CY082846 | A/Swine/Texas/02361/2008 | 10/10/08 | | Human |  |
| 1485 | CY082948 | A/Swine/Texas/02512/2008 | 12/26/08 | | Human |  |
| 1486 | ACV42040 | A/Swine/Texas/050593/2008 |  | | Human (7) | ACV42036 |
| 1487 | ACV42051 | A/Swine/Texas/050625/2008 |  | | Human (7) | ACV42047 |
| 1488 | CY082189 | A/Swine/Virginia/01183/2006 | 8/30/06 | | Human |  |
| 1489 | CY082279 | A/Swine/Virginia/01318/2006 | 11/22/06 | | Human |  |
| 1490 | CY082289 | A/Swine/Virginia/01334/2006 | 11/28/06 | |  |  |
| 1491 | CY082291 | A/Swine/Virginia/01343/2006 | 11/17/06 | |  |  |
| 1492 | CY082304 | A/Swine/Virginia/01359/2006 | 11/28/06 | |  |  |
| 1493 | CY082328 | A/Swine/Virginia/01402/2006 | 12/19/06 | |  |  |
| 1494 | CY082331 | A/Swine/Virginia/01408/2007 | 1/9/07 | |  |  |
| 1495 | CY081596 | A/Swine/Wisconsin/00055/2003 | 2/11/03 | |  |  |
| 1496 | CY081627 | A/Swine/Wisconsin/00134/2003 | 8/14/03 | |  |  |
| 1497 | CY081655 | A/Swine/Wisconsin/00210/2003 | 11/17/03 | |  |  |
| 1498 | CY081669 | A/Swine/Wisconsin/00232/2003 | 12/18/03 | |  |  |
| 1499 | CY081754 | A/Swine/Wisconsin/00385/2004 | 8/12/04 | |  |  |
| 1500 | CY081770 | A/Swine/Wisconsin/00416/2004 | 9/29/04 | |  |  |
| 1501 | CY081909 | A/Swine/Wisconsin/00705/2005 | 8/9/05 | |  |  |
| 1502 | CY082130 | A/Swine/Wisconsin/01079/2006 | 4/4/06 | |  |  |
| 1503 | CY082262 | A/Swine/Wisconsin/01294/2006 | 10/27/06 | |  |  |
| 1504 | CY082322 | A/Swine/Wisconsin/01389/2006 | 12/26/06 | |  |  |
| 1505 | CY082472 | A/Swine/Wisconsin/01768/2007 | 9/13/07 | |  |  |
| 1506 | CY082700 | A/Swine/Wisconsin/02120/2008 | 4/11/08 | |  |  |
| 1507 | CY082709 | A/Swine/Wisconsin/02131/2008 | 4/11/08 | |  |  |
| 1508 | CY082741 | A/Swine/Wisconsin/02192/2008 |  | |  |  |
| 1509 | CY082852 | A/Swine/Wisconsin/02370/2008 | 8/22/08 | |  |  |
| 1510 | CY082855 | A/Swine/Wisconsin/02376/2008 | 10/9/08 | | Human |  |
| 1511 | CY082856 | A/Swine/Wisconsin/02377/2008 | 10/9/08 | |  |  |
| 1512 | CY082880 | A/Swine/Wisconsin/02415/2008 | 8/22/08 | |  |  |
| 1513 | CY082888 | A/Swine/Wisconsin/02424/2008 | 11/4/08 | |  |  |
| 1514 | CY082921 | A/Swine/Wisconsin/02467/2008 | 12/4/08 | |  |  |
| 1515 | CY081820 | A/turkey/North Carolina/00533/2005 | 3/10/05 | |  |  |
| 1516 | CY081821 | A/turkey/North Carolina/00536/2005 | 3/10/05 | |  |  |
